# Supplementary material for: Germline-targeting HIV-1 Env vaccination induces VRC01-class antibodies with rare insertions
Source: Cell Rep Med. 2023 Apr 11;4(4):101003. doi: 10.1016/j.xcrm.2023.101003 (PMC10140475; doi:10.1016/j.xcrm.2023.101003)
Supplement: Document S1. Figures S1–S5 [file mmc1.pdf]

Cell Reports Medicine, Volume *4*

Supplemental information

Germline-targeting HIV-1 Env vaccination induces VRC01-class antibodies with rare insertions

Tom G. Caniels, Max Medina-Ramírez, Jinsong Zhang, Anita Sarkar, Sonu Kumar, Alex LaBranche, Ronald Derking, Joel D. Allen, Jonne L. Snitselaar, Joan Capella-Pujol, Iván del Moral Sánchez, Anila Yasmeen, Marilyn Diaz, Yoann Aldon, Tom P.L. Bijl, Sravani Venkatayogi, Joshua S. Martin Beem, Amanda Newman, Chuancang Jiang, Wen-Hsin Lee, Maarten Pater, Judith A. Burger, Mariëlle J. van Breemen, Steven W. de Taeye, Kimmo Rantalainen, Celia LaBranche, Kevin O. Saunders, David Montefiori, Gabriel Ozorowski, Andrew B. Ward, Max Crispin, John P. Moore, Per Johan Klasse, Barton F. Haynes, Ian A. Wilson, Kevin Wiehe, Laurent Verkoczy, and Rogier W. Sanders

Figure S1

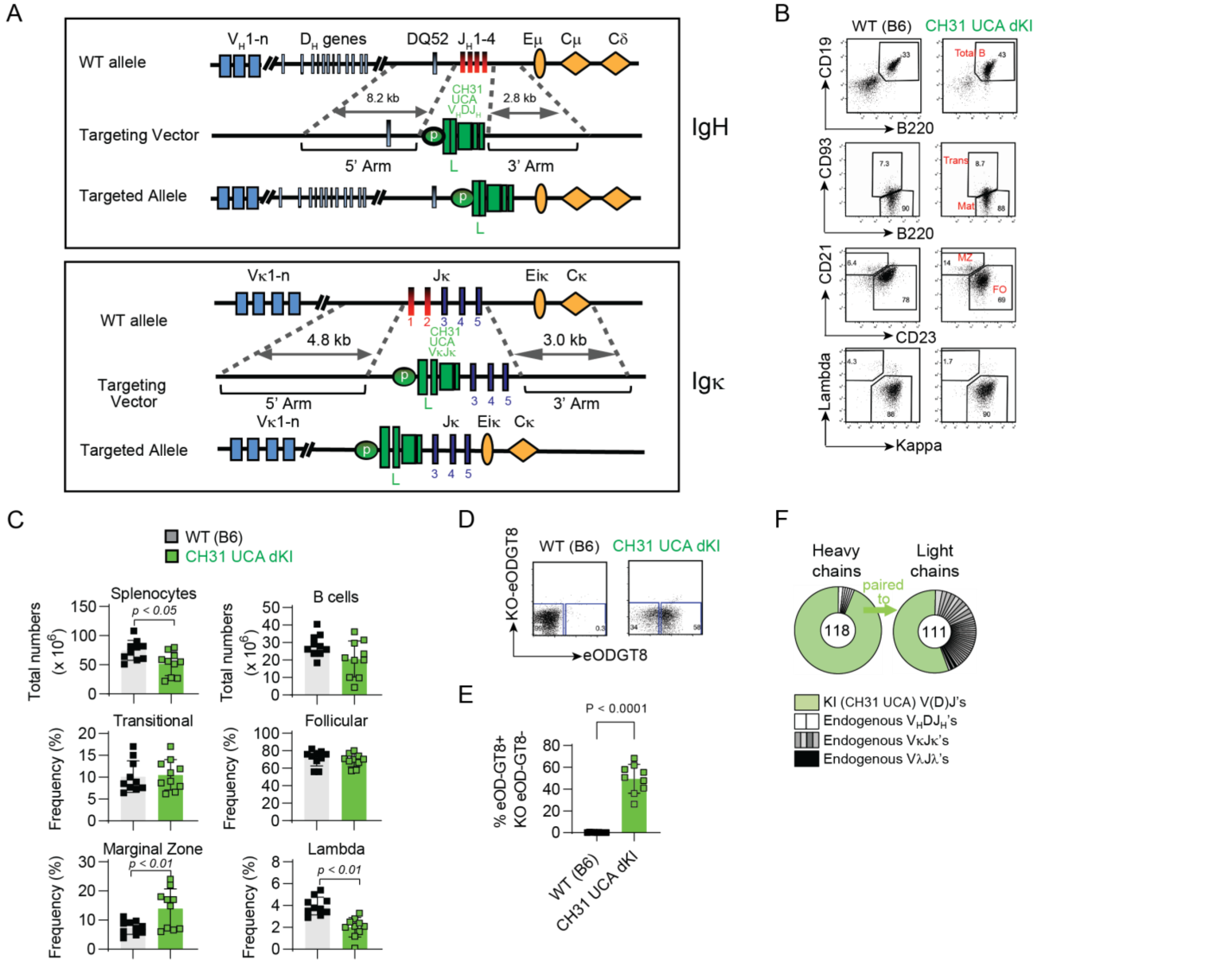

Supplemental Figure 1. Generation and characterization of gl-CH31 KI (V_HDJ_H x VκJκ) mice. Related to Figures 2-6. (A) Schematic depiction of the Ig-directed targeting strategies used to insert the CH31 UCA V_HDJ_H and VκJκ rearrangements into the mouse J_H and Jκ clusters, respectively. In each, top rows represent the wild type Ig loci, middle rows depict the targeting vector DNA donor constructs (illustrating the CH31 expression cassettes between the 5' and 3' homology arms), and bottom rows represent correctly targeted Ig loci, after homologous recombination. The CH31 heavy chain (HC) expression cassette is comprised of the murine H10 V_HJ558 family promoter (p) and split leader (L) in front of the rearranged human CH31 UCA V_HDJ_H coding segment (CH31 UCA V_HDJ_H), while the CH31 light chain (LC) expression cassette contains a VkOx1 promoter (p) and split leader (L) in front of the rearranged CH31 UCA VκJκ coding segment (CH31 UCA VκJκ). (B-C) Developmental B-cell subset analysis in 8-12 wk naïve, fully homozygous ($V_HDJ_H^{+/+}$ x $V\kappa J\kappa^{+/+}$) gl-CH31 KI mice. (B) FACS histograms representative of splenic B-cell development, indicating percentages of total live splenocytes that are $B220^+CD19^+$ (total B-cells; top row), or Transitional (Trans; $B220^{int}$ $CD93^+$) and Mature (Mat; $B220^{hi}$ CD93-) B-cell subsets (2nd row). Total B-cells were also further fractionated into Marginal Zone (MZ; $CD21^{hi}$ $CD23^{lo}$) or Follicular (FO; $CD21^{lo}$ $CD23^{hi}$) B-cell subsets (3rd row), and into kappa+ or lambda+ LC B-cells (bottom row). (C) Graphical representations depicting total numbers of splenocytes (upper left), and, based on gating scheme described in Fig 1B, total numbers of B cells (upper right) or frequencies of Transitional (left middle), Follicular (middle right), Marginal Zone (bottom left), and λLC-expressing B cells (bottom right) in the spleens of wild-type C57BL/6 (WT B6) and CH31 V_HDJ_H KI mice. Individual WT B6 or gl-CH31 KI mice are indicated by closed or open squares, respectively. Standard deviations are denoted by black error bars, and significance values were determined by a two-

tailed Student's t-test, with only significant differences denoted. (D-F) Ig repertoire analysis in heterozygous ($V_HDJ_H^{+/-}$ x $V\kappa J\kappa^{+/-}$) gl-CH31 KI mice. (D) Flow histogram reactivity profile of heterozygous gl-CH31 KI peripheral compartment for CD4bs binding that is prototypical of the CD4 mimic bNAb class. Total peripheral B-cells (singlet, live, $CD19^+$ $B220^+$ splenocytes) from 8-12 week naïve, heterozygous gl-CH31 KI mice were further gated for CD4bs VRC01-class specificity by staining with a WT and mutant (KO) pair of fluorescently labeled eOD-GT8 multimer baits, with 'on-target' binders defined by staining for WT, but not KO eOD-GT8. (E) Graphical representation of on-target CD4bs+ binders in spleens of naïve heterozygous gl-CH31 KI mice (as determined above for Fig. S1D), in relation to baseline levels of binding in age-matched, naïve WT B6 mice. Circles represent individual mice, with group means denoted by black bars. The p value was calculated using a two-tailed student's t-test. (F) HC/LC usage in the peripheral B-cell repertoire of naïve heterozygous gl-CH31 KI mice (n=3). Shown are pie charts of HC/LC rearrangement pairs from individually-sorted B-cells from the total (unselected) splenic B-cell repertoire, with a breakdown of either the knocked-in human Vκ1-33-bearing LC rearrangement (green slices; right pie) or other endogenous (VκJκ or VλJλ rearrangements used; denoted by gray or black slices, respectively), amongst LCs from single cells, paired to the knocked-in human CH31 UCA V_H1-2-bearing HC rearrangement (green slices; left pie). Middle circles represent number of single-cell rearrangements sequenced.

Figure S2

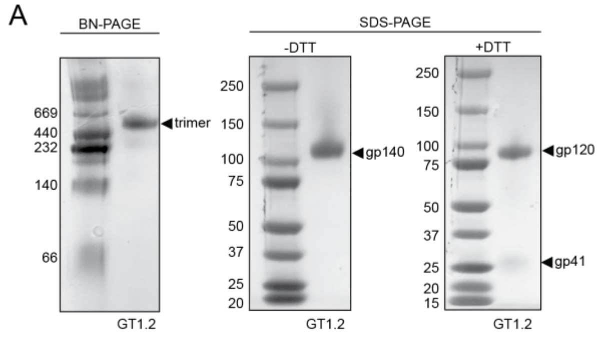

B

GT1.2	N88	N133	N142	N156	N160	N188	N234	N262	N295	N301	N332	N339	N355	N363	N392	N398	N406	N411	N448	N611	N618	N625	N637
M9Glc	0			0	0	0	0	2	0		0	0	0	0				0	0	0	0		0
M9	0			11	13	0	18	77	63		58	37	0	5				0	39	0	0		0
M8	0			35	33	5	45	18	25		28	40	2	37				31	36	0	0		3
M7	2			38	11	7	21	2	7		14	4	4	27				29	12	1	0		12
M6	6	n.d.	n.d.	0	8	6	9	0	2	n.d.	0	2	3	13	n.d.	n.d.	n.d.	16	3	1	0	n.d.	6
M5	11			16	15	14	5	0	2		0	2	17	4				21	4	3	3		10
M4	0			0	4	1	0	0	0		0	1	0	0				0	1	0	0		0
M3	0			0	0	0	0	0	0		0	0	0	0				0	0	0	0		0
FM	0			0	0	0	0	0	0		0	0	0	0				0	0	0	0		0
Hybrid	3			0	1	2	0	0	0		0	0	2	0				0	1	0	0		4
Fhybrid	0			0	0	2	0	0	0		0	0	2	0				0	0	0	0		2
HexNAc(3)(x)	4			0	1	2	1	0	0		0	0	1	0				0	1	0	0		3
HexNAc(3)(F)(x)	1			0	1	8	0	0	0		0	0	13	0				0	1	1	3		7
HexNAc(4)(x)	14			0	1	0	0	0	0		0	0	0	2				0	0	0	0		0
HexNAc(4)(F)(x)	12			0	7	30	0	0	0		0	3	33	3				0	1	3	30		37
HexNAc(5)(x)	15	n.d.	n.d.	0	0	0	0	0	0	n.d.	0	0	0	0	n.d.	n.d.	n.d.	0	0	0	0	n.d.	0
HexNAc(5)(F)(x)	21			0	4	15	0	0	0		0	0	19	0				0	0	14	25		12
HexNAc(6+)(x)	2			0	0	0	0	0	0		0	0	0	0				0	0	1	0		0
HexNAc(6+)(F)(x)	8			0	0	2	0	0	0		0	0	1	0				0	0	3	13		1
Core	0			0	0	2	0	0	0		0	1	2	0				0	1	0	0		0
Unoccupied	1			0	0	3	0	0	0		0	11	0	8				3	0	74	25		2
Oligomannose	20			100	84	33	98	100	99		100	85	27	86				97	95	4	3		32
Hybrid	3			0	1	4	0	0	0		0	0	4	0				0	1	0	0		6
Complex	76			0	15	60	1	0	1		0	4	69	5				0	4	22	71		60
Unoccupied	1	n.d.	n.d.	0	0	3	0	0	0	n.d.	0	11	0	8	n.d.	n.d.	n.d.	3	0	74	25	n.d.	2
Fucose	42			0	13	57	0	0	0		0	3	68	4				0	2	21	71		58
NeuAc	20			0	1	18	0	0	0		0	0	12	0				0	0	1	9		15
Sulfated	0			0	0	0	0	0	0		0	0	0	0				0	0	0	0		0

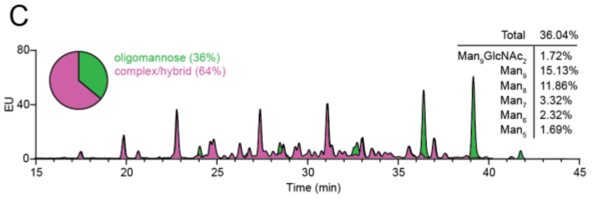

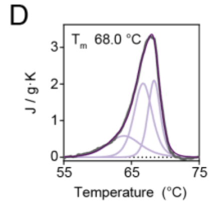

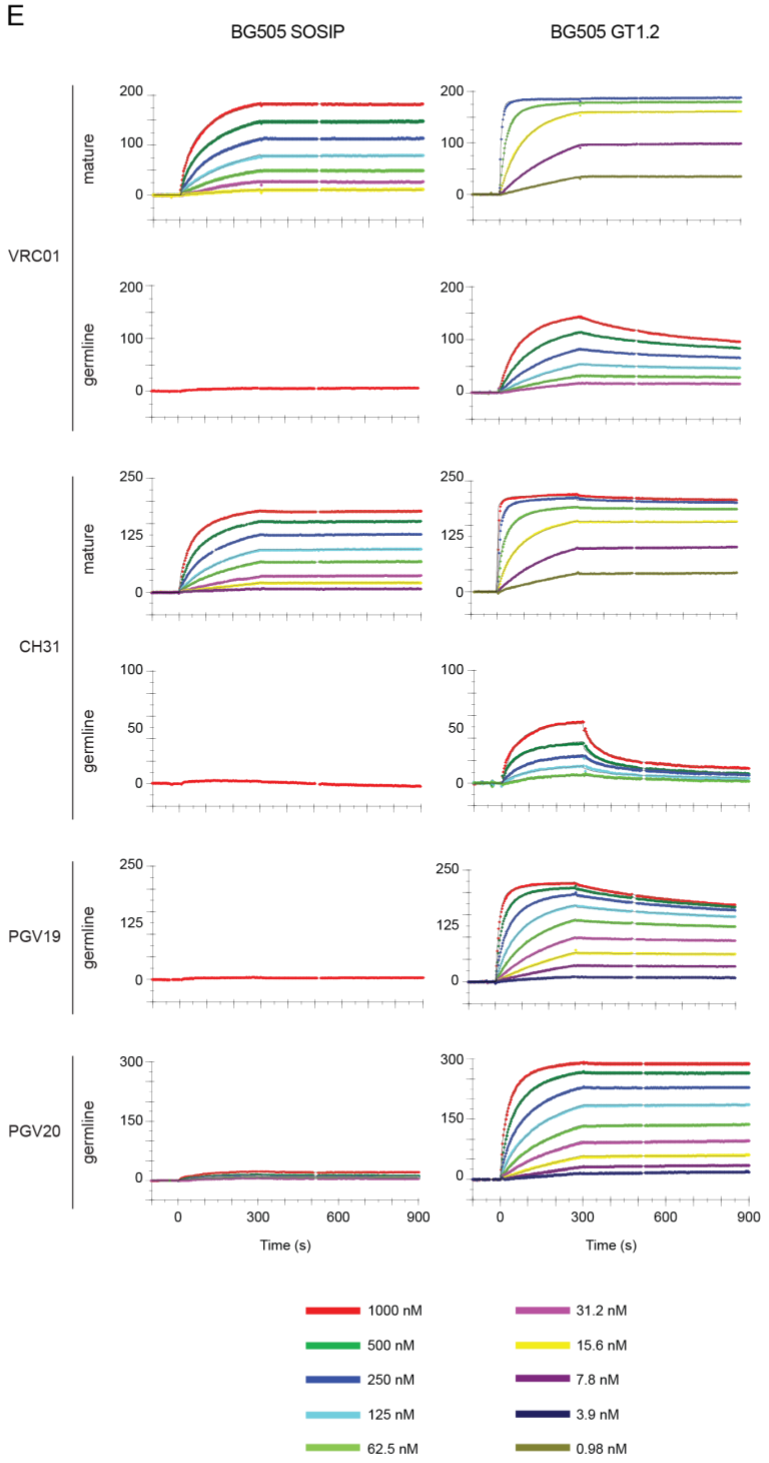

Supplemental Figure 2. Biochemical and antigenic characterization of BG505 GT1.2. Related to Figure 1. (A) Blue-native polyacrylamide gel electrophoresis (BN-PAGE) and sodium dodecyl sulfate PAGE (SDS-PAGE) in non-reducing (-DTT) and reducing (+DTT) conditions. (B) The data sets show the glycoforms found at each PNGS. Data for oligomannose/hybrid-type glycans are shaded in green, fully processed complex type glycans are shaded in magenta, while the absence of a glycan from some PNGS is shaded in grey. Oligomannose-type glycans are categorized according to the number of mannose residues present, hybrids by the presence/absence of fucose and complex-type glycans by the number of processed antenna and the presence/absence of fucose. (C) HILIC-UPLC analysis of BG505 GT1.2. Peaks colored green represent endoH-cleavable glycans (oligomannose/hybrid). The spectrum shows the overall oligomannose (green) and complex/hybrid (magenta) content. (D) Differential scanning calorimetry (DSC) analysis of BG505 GT1.2. The Tm value is indicated. (E) The sensorgrams show the specific binding signal in response units (RU) on the y-axes as a function of time during association and dissociation on the x-axes

(s). The color-coded legend gives the NAb concentrations for the empirical binding curves; the curves fitted with a model for bivalent binding are in black.

Figure S3

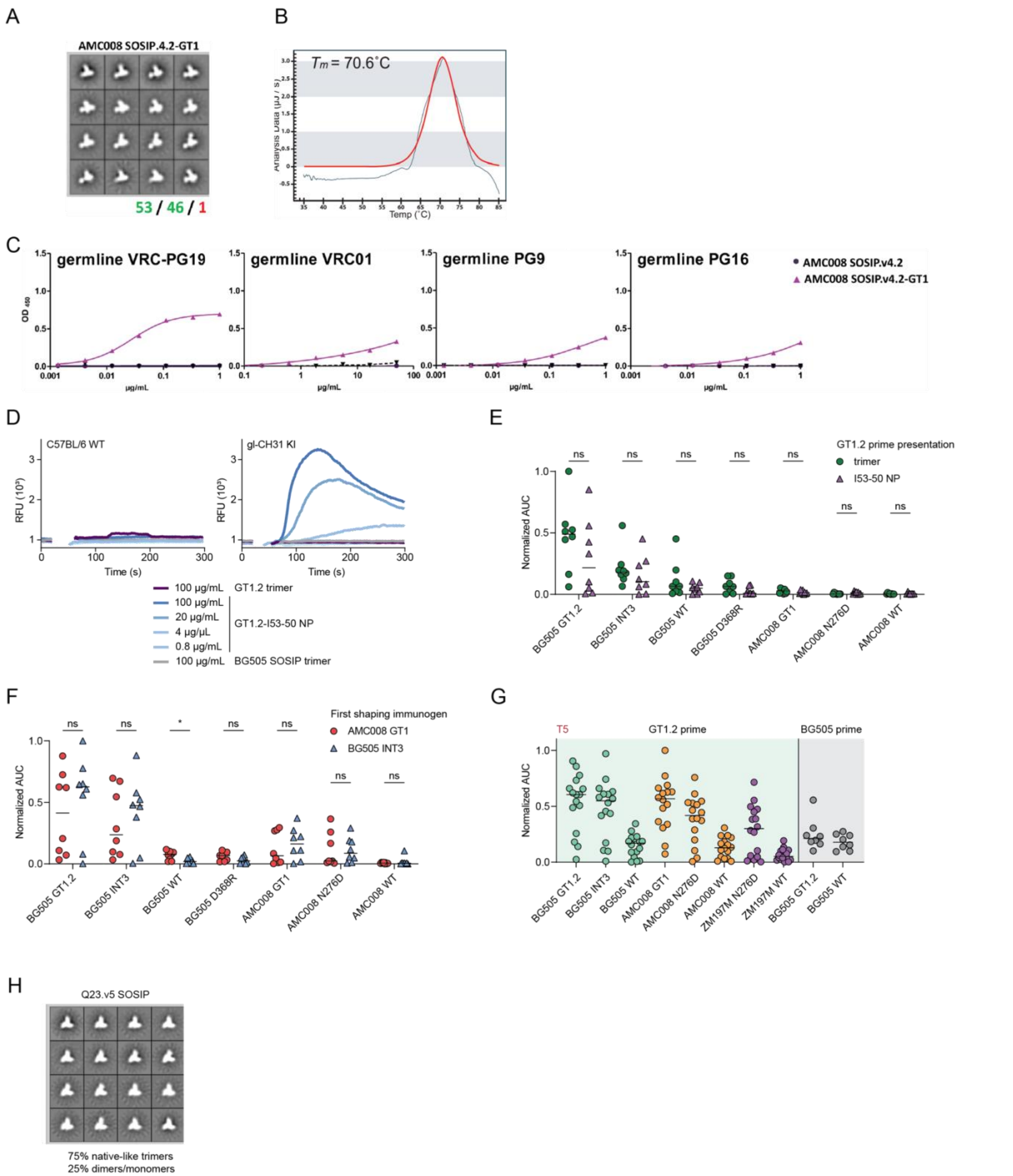

Supplemental Figure 3. Stability and antigenicity of AMC008 GT1, *ex vivo* gl-CH31 B cell activation and serum antibody binding of immunized animals. Related to Figures 1 and 2 (A) The AMC008 SOSIP.v4.2 clade B trimer (de Taeye et al., 2015) is based on an early sequence of an individual participating in the ACS that eventually developed bNAbs. We made the corresponding AMC008 SOSIP.v4.2-GT1 trimers that bear the same mutations as found in BG505 GT1. The resulting trimers are native-like and largely present as closed trimers. The numbers represent the percentage of native-like closed/native-like open/non-native-like trimers. (B) Differential scanning calorimetry (DSC) analysis of AMC008 GT1. The Tm value is indicated (Tm, 70.6°C). (C) ELISA graphs showing AMC008 SOSIP.v4.2 trimers were unable to bind any of the gl-bNAbs tested at the

maximum concentration. However, its corresponding GT1 version bound efficiently to gl-PGV19, gl-PG9 and gl-PG16 and also reacted with gl-VRC01, albeit more weakly. (D) Calcium flux assay as a measure for B cell activation from WT C57BL/6 B cells (left) and gl-CH31 KI B cells with the proteins indicated below. (E) Normalized area-under-the-curve (AUC) values of serum antibody binding to the indicated Env as measured by enzyme-linked immunosorbent assay (ELISA) at time point T2 (Fig. 2D). Each dot represents an individual mouse and the colors represent the priming immunogen received (green, GT1.2 trimer; purple, GT.2-I53-50 nanoparticles). (F) Normalized AUC values of serum antibody binding to the indicated Env at time point T3. Each dot represents an individual mouse and the colors represent the first shaping immunogen received (red, AMC008 GT1; blue, BG505 INT3). (G) Normalized AUC values of serum antibody binding to the indicated Env at time point T5, depicted as in Fig. 2E-G. Each dot represents an individual mouse and the background color represents the priming group (GT1.2 trimer/NP vs. BG505). The shading of the dots indicates the clade of the Env trimer (green, A; orange, B; purple, C).

Figure S4

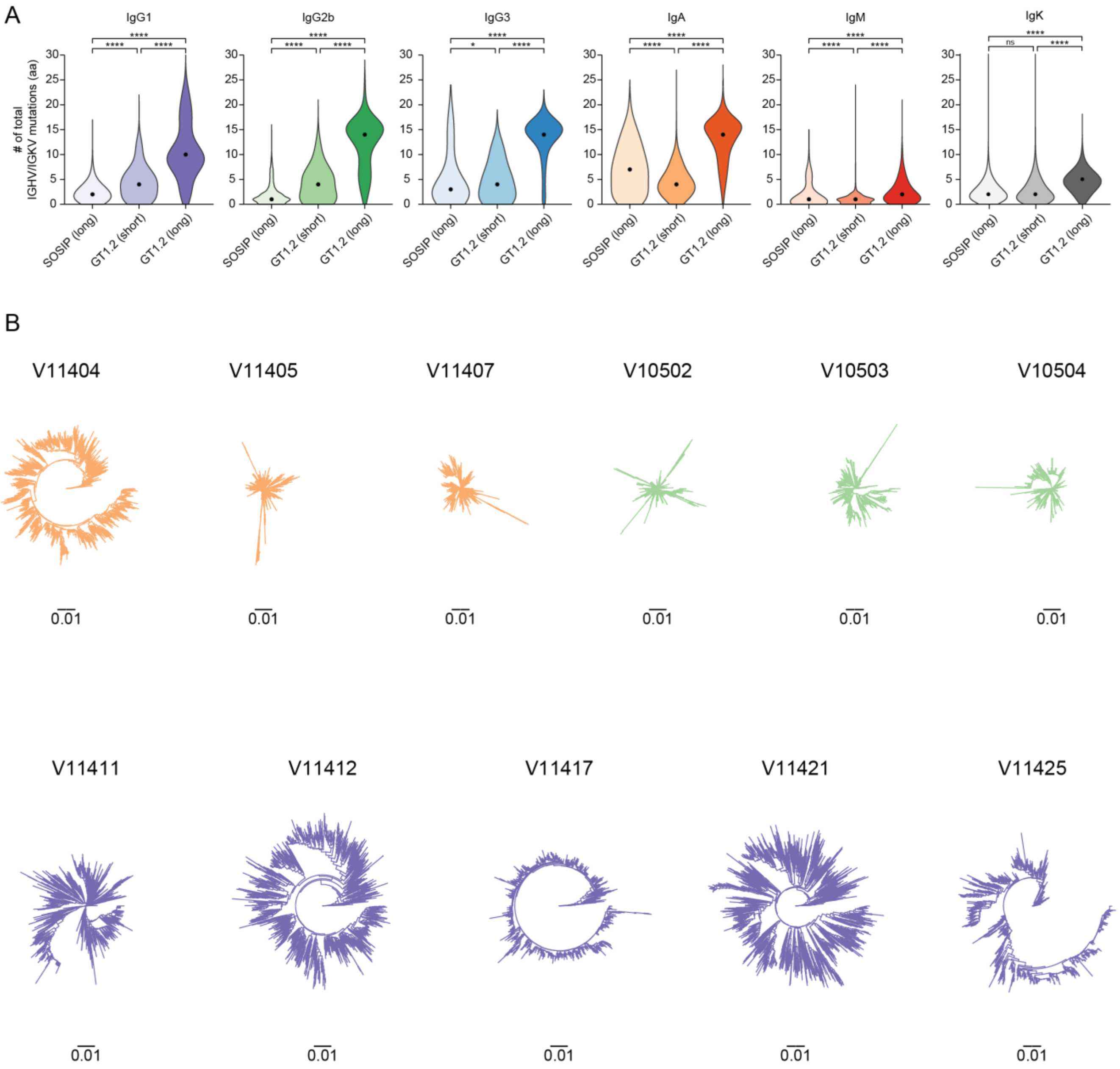

Supplemental Figure 4. Nonsynonymous mutations induced by immunization regimens and phylogenetic reconstructions. Related to Figure 3. (A) Violin plot showing the number of total amino acid substitutions in the IGHV region for each group (x-axes) for each Ig subtype. *, $p < 0.05$; ****, $p < 0.0001$. (B) Clonal diversification of immunized mice (each tree represents one mouse) as shown by circular trees of randomly sampled reads from the repertoires of each immunized mouse in the SOSIP-primed (orange), “GT1.2 (short)” (green) and “GT1.2 (long)” (purple) groups. Scale is set to a phylogenetic distance of 0.01 nucleotide substitutions per site for all trees and the trees are rooted on the gl-CH31 sequence.

Figure S5

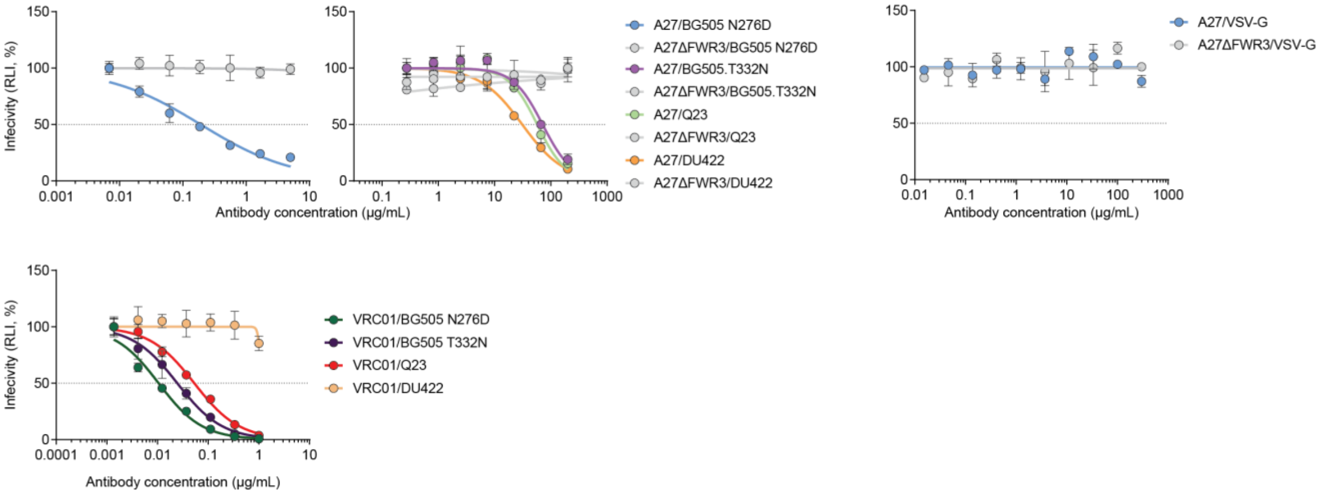

Supplemental Figure 5. A27 and A27ΔFWR3 neutralization. Related to Figures 4 and 5. The dotted line represents the midpoint neutralization titer (IC_{50}). Each dot represents the mean of two independent experiments (n=2) performed in triplicate.

NAb (IgG)		Env trimer	k_{on1} (1/Ms)	k_{off1} (1/s)	K_{D1} (nM)	k_{on2} (1/Ms)	k_{off2} (1/s)	K_{D2} (nM)	S_m
**VRC01**	*mature*	BG505 SOSIP.v4.1 (n=3)	$9.6 \cdot 10^3$ ± 3.5 10^2	< 10^{-5}	< 10	$6.8 \cdot 10^4$ ± $9.8 \cdot 10^3$	$9.2 \cdot 10^{-2}$ ± $1.5 \cdot 10^{-2}$	$1.3 \cdot 10^3$ ± 53	2.2 ± $9.3 \cdot 10^{-2}$
		BG505 SOSIP.v4.1-GT1.2 (n=2)	$2.6 \cdot 10^5$ ± $5.0 \cdot 10^3$	< 10^{-5}	< 0.10	$1.3 \cdot 10^3$ ± 15	$2.1 \cdot 10^{-3}$ ± 0	$1.7 \cdot 10^3$ ± 20	2.1 ± $4.9 \cdot 10^{-3}$
	germline	BG505 SOSIP.v4.1 (n=2)	Minimal binding; <10 RU						
		BG505 SOSIP.v4.1-GT1.2 (n=2)	$3.9 \cdot 10^3$ ± 60	$2.6 \cdot 10^{-3}$ ± $1.1 \cdot 10^{-4}$	$6.7 \cdot 10^2$ ± 37	$3.8 \cdot 10^7$ ± $1.1 \cdot 10^7$	20 ± 5.8	$5.4 \cdot 10^2$ ± 7.3	2.7 ± $8.5 \cdot 10^{-3}$
**CH31**	*mature*	BG505 SOSIP.v4.1 (n=3)	$1.4 \cdot 10^4$ ± $3.1 \cdot 10^2$	< 10^{-5}	< 1.0	$8.6 \cdot 10^4$ ± $3.6 \cdot 10^4$	0.14 ± $6.2 \cdot 10^{-2}$	$1.7 \cdot 10^3$ ± 54	2.2 ± $9.5 \cdot 10^{-2}$
		BG505 SOSIP.v4.1-GT1.2 (n=3)	$3.7 \cdot 10^5$ ± $2.2 \cdot 10^4$	$5.0 \cdot 10^{-5}$ ± $8.2 \cdot 10^{-6}$	0.14 ± $3.2 \cdot 10^{-2}$	$1.7 \cdot 10^4$ ± $2.8 \cdot 10^2$	$2.5 \cdot 10^{-2}$ ± $4.6 \cdot 10^{-3}$	$1.5 \cdot 10^3$ ± $2.7 \cdot 10^2$	2.2 ±$4.3 \cdot 10^{-2}$
	UCA	BG505 SOSIP.v4.1 (n=2)	Minimal binding; <10 RU						
		BG505 SOSIP.v4.1-GT1.2 (n=2)	$3.7 \cdot 10^3$ ± 50	$1.5 \cdot 10^{-2}$ ± $1.5 \cdot 10^{-3}$	$4.0 \cdot 10^3$ ± $3.6 \cdot 10^2$	$5.1 \cdot 10^2$ ± 60	$6.9 \cdot 10^{-4}$ ± $4.0 \cdot 10^{-5}$	$1.4 \cdot 10^3$ ± $2.4 \cdot 10^2$	1.4 ± 0.10
**PGV19**	*germline*	BG505 SOSIP.v4.1 (n=2)	Minimal binding; <10 RU						
		BG505 SOSIP.v4.1-GT1.2 (n=2)	$2.6 \cdot 10^4$ ± $2.5 \cdot 10^2$	$4.9 \cdot 10^{-4}$ ± $5.5 \cdot 10^{-6}$	19 ± 0.39	$2.1 \cdot 10^3$ ± 60	$2.4 \cdot 10^{-3}$ ± $3.0 \cdot 10^{-5}$	$1.1 \cdot 10^3$ ± 48	2.3 ± 0.10
**PGV20**	*germline*	BG505 SOSIP.v4.1 (n=2)	$9.9 \cdot 10^3$ ± $2.0 \cdot 10^3$	$2.3 \cdot 10^{-4}$ ± $4.0 \cdot 10^{-5}$	23 ± 0.57	$6.2 \cdot 10^5$ ± $4.3 \cdot 10^5$	0.32 ± 0.27	$4.1 \cdot 10^2$ ± $1.5 \cdot 10^2$	0.28 ± $3.7 \cdot 10^{-3}$
		BG505 SOSIP.v4.1-GT1.2 (n=3)	$3.4 \cdot 10^4$ ± $6.6 \cdot 10^3$	< 10^{-5}	< 1.0	$3.5 \cdot 10^6$ ± $3.3 \cdot 10^6$	5.7 ± 5.2	$2.2 \cdot 10^3$ ± $2.8 \cdot 10^2$	3.0 ± $3.3 \cdot 10^{-2}$

Table S1. SPR analysis of mature and germline/UCA NAb binding to BG505 SOSIP.v4.1 and GT1.2. Tabulated values are means ± S.E.M of n replicates. The parameters are fitted to the specific sensorgram binding data with a bivalent model; the constants for the initial, monovalent interaction are subscripted 1; for the interaction by the second Fab-arm of the IgG they are subscripted 2.

Data collection	BG505 SOSIP.v4.1-GT1.2 + Fab PGT124 + Fab gl-PGV20
Beamline	APS 23-IDD
Wavelength (Å)	1.0332
Detector	Pilatus
Space group	$P12_11$
Unit cell parameters (Å)	
a, b, c (Å), °	146.0, 157.6, 158.5, β=102.9
Resolution (Å)	49.7-3.8 (3.88-3.82)[a]
Total reflections	63,948
Unique reflections	5699
Redundancy	2.9 (3.0)[a]
Completeness (%)	95.7 (96.9)[a]
$<I/\sigma_i>$	4.3 (0.8)[a]
R_{sym}[b]	0.28 (>1.00)[a]
R_{pim}[c]	0.15 (0.73)[a]
$CC_{1/2}$[d]	0.78 (0.31)[a]
Wilson *B*-value ($Å^2$)	104.9
**Refinement statistics**	
Resolution (Å)	49.7-3.8 (3.9-3.8)[a]
Reflections (work)	63,928
Reflections (test)	3,180
R_{cryst}(%)[e]	27.0
R_{free}(%)[f]	30.1
Average B value ($Å^2$) (Proteins/Glycans)	122/115
**RMSD from ideal geometry**	
Bond length (Å)	0.003
Bond angles (°)	0.54
**Ramachandran statistics (%)**	
Favored	93.24
Allowed	6.08
Outliers	0.68
**PDB ID**	8E1P

[a]Numbers in parentheses are for highest resolution shell
[b]$R_{sym} = \Sigma_{hkl} \Sigma_i | I_{hkl,i} - <I_{hkl}> | / \Sigma_{hkl} \Sigma_i I_{hkl,i}$, where $I_{hkl,i}$ is the scaled intensity of the ith measurement of reflection h, k, l, and $< I_{hkl}>$ is the average intensity for that reflection
[c]$R_{pim} = \Sigma_{hkl} (1/(n-1))1/2 \Sigma_i | I_{hkl,i} - <I_{hkl}> | / \Sigma_{hkl} \Sigma_i I_{hkl,i}$, where n is the redundancy
[d]$CC_{1/2}$ = Pearson Correlation Coefficient between two random half datasets
[e]$R_{cryst} = \Sigma_{hkl} | Fo - Fc | / \Sigma_{hkl} | Fo | \times 100$
[f]R_{free} was calculated as for R_{cryst}, but on a test set comprising 5% of the data excluded from refinement

Table S2. X-ray data collection and refinement statistics. The data has been deposited in the Protein Data Bank under accession number 8E1P.
