# Supplementary material for: Germline-targeting HIV-1 Env vaccination induces VRC01-class antibodies with rare insertions
Source: Cell Rep Med. 2023 Apr 11;4(4):101003. doi: 10.1016/j.xcrm.2023.101003 (PMC10140475; doi:10.1016/j.xcrm.2023.101003)
Supplement: Table S1. SPR analysis of mature and germline/UCA NAb binding to BG505 SOSIP.v4.1 and GT1.2 — Tabulated values are means ± S.E.M of n replicates. The parameters are fitted to the specific sensorgram binding data with a bivalent model; the constants for the initial, monovalent interaction are subscripted 1; for the interaction by the second Fab-arm of the IgG, they are subscripted 2. [file mmc2.docx]

| **NAb (IgG)** | | **Env trimer** | ***k_on1_***  **(1/Ms)** | ***k_off1_***  **(1/s)** | ***K_D1_***  **(nM)** | ***k_on2_***  **(1/Ms)** | ***k_off2_***  **(1/s)** | ***K_D2_***  **(nM)** | ***S_m_*** |
| --- | --- | --- | --- | --- | --- | --- | --- | --- | --- |
| **VRC01** | *mature* | BG505 SOSIP.v4.1  (n=3) | 9.6 **^.^** 10^3^  ± 3.5 10^2^ | < 10^-5^ | < 10 | 6.8 **^.^** 10^4^  ± 9.8 **^.^** 10^3^ | 9.2 **^.^** 10^-2^  ± 1.5 **^.^** 10^-2^ | 1.3 **^.^** 10^3^  ± 53 | 2.2  ± 9.3 **^.^** 10^-2^ |
|  |  | BG505 SOSIP.v4.1-GT1.2  (n=2) | 2.6 **^.^** 10^5^  ± 5.0 **^.^** 10^3^ | < 10^-5^ | < 0.10 | 1.3 **^.^** 10^3^  ± 15 | 2.1 **^.^** 10^-3^  ± 0 | 1.7 **^.^** 10^3^  ± 20 | 2.1  ± 4.9 **^.^** 10^-3^ |
|  | *germline* | BG505 SOSIP.v4.1  (n=2) | Minimal binding; <10 RU | | | | | | |
|  |  | BG505 SOSIP.v4.1-GT1.2  (n=2) | 3.9 **^.^** 10^3^  ± 60 | 2.6 **^.^** 10^-3^  ± 1.1 **^.^** 10^-4^ | 6.7 **^.^** 10^2^  ± 37 | 3.8 **^.^** 10^7^  ± 1.1 **^.^** 10^7^ | 20  ± 5.8 | 5.4 **^.^** 10^2^  ± 7.3 | 2.7  ± 8.5 **^.^** 10^-3^ |
| **CH31** | *mature* | BG505 SOSIP.v4.1  (n=3) | 1.4 **^.^** 10^4^  ± 3.1 **^.^** 10^2^ | < 10^-5^ | < 1.0 | 8.6 **^.^** 10^4^  ± 3.6 **^.^** 10^4^ | 0.14  ± 6.2 **^.^** 10^-2^ | 1.7 **^.^** 10^3^  ± 54 | 2.2  ± 9.5 **^.^** 10^-2^ |
|  |  | BG505 SOSIP.v4.1-GT1.2  (n=3) | 3.7 **^.^** 10^5^  ± 2.2 **^.^** 10^4^ | 5.0 **^.^** 10^-5^  ± 8.2 **^.^** 10^-6^ | 0.14  ± 3.2 **^.^** 10^-2^ | 1.7 **^.^** 10^4^  ± 2.8 **^.^** 10^2^ | 2.5 **^.^** 10^-2^  ± 4.6 **^.^** 10^-3^ | 1.5 **^.^** 10^3^  ± 2.7 **^.^** 10^2^ | 2.2  ±4.3 **^.^** 10^-2^ |
|  | *UCA* | BG505 SOSIP.v4.1  (n=2) | Minimal binding; <10 RU | | | | | | |
|  |  | BG505 SOSIP.v4.1-GT1.2  (n=2) | 3.7 **^.^** 10^3^  ± 50 | 1.5 **^.^** 10^-2^  ± 1.5 **^.^** 10^-3^ | 4.0 **^.^** 10^3^  ± 3.6 **^.^** 10^2^ | 5.1 **^.^** 10^2^  ± 60 | 6.9 **^.^** 10^-4^  ± 4.0 **^.^** 10^-5^ | 1.4 **^.^** 10^3^  ± 2.4 **^.^** 10^2^ | 1.4  ± 0.10 |
| **PGV19** | *germline* | BG505 SOSIP.v4.1  (n=2) | Minimal binding; <10 RU | | | | | | |
|  |  | BG505 SOSIP.v4.1-GT1.2  (n=2) | 2.6 **^.^** 10^4^  ± 2.5 **^.^** 10^2^ | 4.9 **^.^** 10^-4^  ± 5.5 **^.^** 10^-6^ | 19  ± 0.39 | 2.1 **^.^** 10^3^  ± 60 | 2.4 **^.^** 10^-3^  ± 3.0 **^.^** 10^-5^ | 1.1 **^.^** 10^3^  ± 48 | 2.3  ± 0.10 |
| **PGV20** | *germline* | BG505 SOSIP.v4.1  (n=2) | 9.9 **^.^** 10^3^  ± 2.0 **^.^** 10^3^ | 2.3 **^.^** 10^-4^  ± 4.0 **^.^** 10^-5^ | 23  ± 0.57 | 6.2 **^.^** 10^5^  ± 4.3 **^.^** 10^5^ | 0.32  ± 0.27 | 4.1 **^.^** 10^2^  ± 1.5 **^.^** 10^2^ | 0.28  ± 3.7 **^.^** 10^-3^ |
|  |  | BG505 SOSIP.v4.1-GT1.2  (n=3) | 3.4 **^.^** 10^4^  ± 6.6 **^.^** 10^3^ | < 10^-5^ | < 1.0 | 3.5 **^.^** 10^6^  ± 3.3 **^.^** 10^6^ | 5.7  ± 5.2 | 2.2 **^.^** 10^3^  ± 2.8 **^.^** 10^2^ | 3.0  ± 3.3 **^.^** 10^-2^ |

**SPR analysis of mature and germline/UCA NAb binding to BG505 SOSIP.v4.1 and GT1.2 ^a^**

**^a^** Tabulated values are means ± S.E.M of n replicates. The parameters are fitted to the specific sensorgram binding data with a bivalent model; the constants for the initial, monovalent interaction are subscripted 1; for the interaction by the second Fab-arm of the IgG they are subscripted 2.
