# Supplementary material for: Germline-targeting HIV-1 Env vaccination induces VRC01-class antibodies with rare insertions
Source: Cell Rep Med. 2023 Apr 11;4(4):101003. doi: 10.1016/j.xcrm.2023.101003 (PMC10140475; doi:10.1016/j.xcrm.2023.101003)
Supplement: Table S2. X-ray data collection and refinement statistics [file mmc3.docx]

**Table S2. X-ray data collection and refinement statistics.**

**Data collection BG505 SOSIP.v4.1-GT1.2 + Fab PGT124 + Fab gl-PGV20**

| Beamline | APS 23-IDD |
| --- | --- |
| Wavelength (Å) | 1.0332 |
| Detector | Pilatus |
| Space group | P12_1_1 |
| Unit cell parameters (Å)  a, b, c (Å), ° | 146.0, 157.6, 158.5, β=102.9 |
| Resolution (Å) | 49.7-3.8 (3.88-3.82)^a^ |
| Total reflections | 63,948 |
| Unique reflections | 5699 |
| Redundancy | 2.9 (3.0)^a^ |
| Completeness (%) | 95.7 (96.9)^a^ |
| <I/σ_i_> | 4.3 (0.8)^a^ |
| R_sym_^b^ | 0.28 (>1.00)^a^ |
| R_pim_^c^ | 0.15 (0.73)^a^ |
| CC_1/2_^d^ | 0.78 (0.31)^a^ |
| Wilson *B*-value (Å^2^) | 104.9 |

**Refinement statistics**

| Resolution (Å) | 49.7-3.8 (3.9-3.8)^a^ |
| --- | --- |
| Reflections (work) | 63,928 |
| Reflections (test) | 3,180 |
| R_cryst_(%)^e^ | 27.0 |
| R_free_(%)^f^ | 30.1 |
| Average B value (Å^2^) (Proteins/Glycans) | 122/115 |

**RMSD from ideal geometry**

| Bond length (Å) | 0.003 |
| --- | --- |
| Bond angles (°) | 0.54 |

**Ramachandran statistics (%)**

| Favored | 93.24 |
| --- | --- |
| Allowed | 6.08 |
| Outliers | 0.68 |
| **PDB ID** | 8E1P |

^a^Numbers in parentheses are for highest resolution shell

^b^R_sym_ = Σ_hkl_ Σ_i_ | I_hkl,i_ - <I_hkl_> | / Σ_hkl_ Σ_i_ I_hkl,i,_ where I_hkl,i_ is the scaled intensity of the i^th^ measurement of reflection h, k, l, and < I_hkl_> is the average intensity for that reflection

^c^R_pim_ = Σ_hkl_ (1/(n-1))1/2 Σ_i_ | I_hkl,i_ - <I_hkl_> | / Σ_hkl_ Σ_i_ I_hkl,i,_ where n is the redundancy

^d^CC_1/2_ = Pearson Correlation Coefficient between two random half datasets

^e^R_cryst_ = Σ_hkl_ | Fo - Fc | / Σ_hkl_ | Fo | x 100

^f^R_free_ was calculated as for R_cryst_, but on a test set comprising 5% of the data excluded from refinement
