# Supplementary material for: Germline-targeting HIV-1 Env vaccination induces VRC01-class antibodies with rare insertions
Source: Cell Rep Med. 2023 Apr 11;4(4):101003. doi: 10.1016/j.xcrm.2023.101003 (PMC10140475; doi:10.1016/j.xcrm.2023.101003)
Supplement: Document S2. Article plus supplemental information [file mmc6.pdf]

Cell Reports
Medicine

Article

Germline-targeting HIV-1 Env vaccination induces VRC01-class antibodies with rare insertions

Graphical abstract

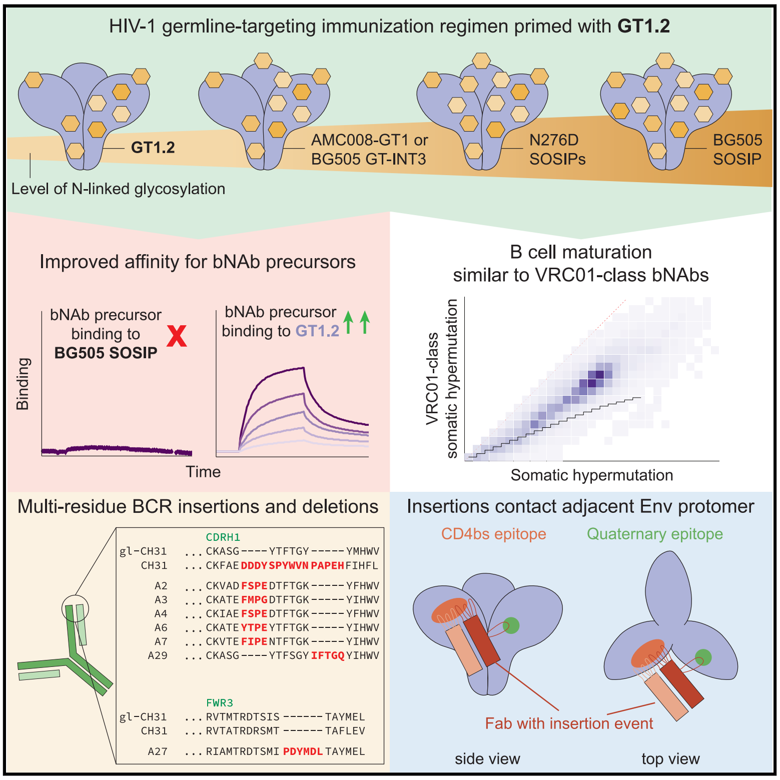

Highlights

- Germline-targeting HIV-1 Env SOSIP GT1.2 activates bNAb precursors in a mouse model
- B cells display VRC01-class maturation and multi-residue insertions and deletions
- Isolated VRC01-class mAbs neutralize multiple N276 glycan-containing pseudoviruses
- Multi-residue insertions are necessary for mAb neutralization

Authors

Tom G. Caniels, Max Medina-Ramírez, Jinsong Zhang, ..., Kevin Wiehe, Laurent Verkoczy, Rogier W. Sanders

Correspondence

laurent.verkoczy@absinstitute.org (L.V.),
r.w.sanders@amsterdamumc.nl (R.W.S.)

In brief

The induction of bNAbs is essential for a protective HIV-1 vaccine, but current vaccines are unable to induce sufficient B cell maturation. Caniels et al. describe an immunization regimen that elicits neutralizing antibodies toward the CD4bs and isolates monoclonal antibodies with rare sequence features that resemble bNAbs.

Caniels et al., 2023, Cell Reports Medicine *4*, 101003
April 18, 2023 © 2023 The Author(s).
https://doi.org/10.1016/j.xcrm.2023.101003

CellPress

Cell Reports Medicine

CellPress
OPEN ACCESS

Article

Germline-targeting HIV-1 Env vaccination induces VRC01-class antibodies with rare insertions

Tom G. Caniels,[1,2] Max Medina-Ramírez,[1,2] Jinsong Zhang,[3] Anita Sarkar,[4] Sonu Kumar,[4] Alex LaBranche,[3] Ronald Derking,[1,2] Joel D. Allen,[5] Jonne L. Snitselaar,[1,2] Joan Capella-Pujol,[1,2] Iván del Moral Sánchez,[1,2] Anila Yasmeen,[6] Marilyn Diaz,[3] Yoann Aldon,[1,2] Tom P.L. Bijl,[1,2] Sravani Venkatayogi,[7] Joshua S. Martin Beem,[7] Amanda Newman,[7] Chuancang Jiang,[6] Wen-Hsin Lee,[4] Maarten Pater,[1,2] Judith A. Burger,[1,2] Mariëlle J. van Breemen,[1,2] Steven W. de Taeye,[1,2] Kimmo Rantalainen,[4] Celia LaBranche,[7] Kevin O. Saunders,[7] David Montefiori,[7,8] Gabriel Ozorowski,[4] Andrew B. Ward,[4] Max Crispin,[5] John P. Moore,[6] Per Johan Klasse,[6] Barton F. Haynes,[8] Ian A. Wilson,[4,9] Kevin Wiehe,[8] Laurent Verkoczy,[3,*] and Rogier W. Sanders[1,2,6,10,*]

[1]Department of Medical Microbiology, Amsterdam UMC, University of Amsterdam, Amsterdam, the Netherlands
[2]Amsterdam Institute for Infection and Immunity, Infectious Diseases, Amsterdam, the Netherlands
[3]Applied Biomedical Science Institute, San Diego, CA, USA
[4]Department of Integrative Structural and Computational Biology, The Scripps Research Institute, La Jolla, CA, USA
[5]School of Biological Sciences, University of Southampton, Southampton, UK
[6]Department of Microbiology and Immunology, Weill Medical College of Cornell University, New York, NY, USA
[7]Department of Surgery, Duke University School of Medicine, Durham, NC, USA
[8]Duke Human Vaccine Institute, Duke University School of Medicine, Durham, NC, USA
[9]The Skaggs Institute for Chemical Biology, The Scripps Research Institute, La Jolla, CA, USA
[10]Lead contact
*Correspondence: laurent.verkoczy@absinstitute.org (L.V.), r.w.sanders@amsterdamumc.nl (R.W.S.)
https://doi.org/10.1016/j.xcrm.2023.101003

SUMMARY

Targeting germline (gl-) precursors of broadly neutralizing antibodies (bNAbs) is acknowledged as an important strategy for HIV-1 vaccines. The VRC01-class of bNAbs is attractive because of its distinct genetic signature. However, VRC01-class bNAbs often require extensive somatic hypermutation, including rare insertions and deletions. We describe a BG505 SOSIP trimer, termed GT1.2, to optimize binding to gl-CH31, the unmutated common precursor of the CH30-34 bNAb lineage that acquired a large CDRH1 insertion. The GT1.2 trimer activates gl-CH31 naive B cells in knock-in mice, and B cell responses could be matured by selected boosting immunogens to generate cross-reactive Ab responses. Next-generation B cell sequencing reveals selection for VRC01-class mutations, including insertions in CDRH1 and FWR3 at positions identical to VRC01-class bNAbs, as well as CDRL1 deletions and/or glycine substitutions to accommodate the N276 glycan. These results provide proof of concept for vaccine-induced affinity maturation of B cell lineages that require rare insertions and deletions.

INTRODUCTION

Almost 40 years after the identification of HIV-1, the need for a vaccine remains as urgent as ever. A vaccine will need to confer protection against a plethora of HIV-1 strains and it is likely that an essential component of such a vaccine is to induce broadly neutralizing antibodies (bNAbs). bNAbs are generated by a subset of HIV-1-infected individuals after multiple years of HIV-1 replication and many have been cloned and characterized.[1,2] bNAbs can treat and prevent infection in non-human primate studies and are currently being evaluated in clinical trials for HIV-1 treatment and prevention.[3–6] However, inducing bNAb responses through vaccination in humans remains a major challenge, at least in part because bNAbs require a lengthy and complex process of co-evolution with the virus.[7–10]

The first critical step in bNAb induction is the activation of naive B cells that have the intrinsic capacity to develop bNAbs (germline [gl]-bNAbs). Such B cells are usually present at low frequencies in the human naive B cell repertoire and have no or low affinity for current HIV-1 vaccine candidates, immediately placing these B cells at a selective disadvantage relative to more abundant and higher affinity B cells recognizing other epitopes.[11–16] However, gl-bNAbs can serve as templates for the design of immunogens that selectively activate these rare naive B cells (reviewed in[17,18]). Such immunogens have indeed been generated and in some cases, such as for N332 supersite-targeting gl-PGT121,[19] have led to the induction of NAbs in knock-in (KI) mouse models with high frequencies of HIV-1 bNAb precursors, providing proof-of-concept for "germline targeting" strategies.[18]

Particularly attractive gl-bNAb precursors are those of the VRC01-class. VRC01-class bNAbs target the conserved CD4 binding site (CD4bs) epitope on the Env trimer and use the IGHV1-2*02 gene segment in combination with a light chain

This is an open access article under the CC BY license (http://creativecommons.org/licenses/by/4.0/).

(LC) bearing a short five amino acid LC complementarity determining region 3 loop.[20,21] Some examples of potently neutralizing VRC01-class bNAbs include VRC01, 3BNC60, CH31, 12A12, PGV20, N49P7, and the broadest HIV-1 bNAb described to date, N6.[20–24] All of these VRC01-class bNAbs were isolated from distinct HIV-1 patients, indicating that humans can reproducibly generate such bNAbs. Indeed, VRC01-class precursors can be found in the vast majority of humans at frequencies that are sufficient and practical for germline targeting, an important prerequisite for a viable vaccine strategy.[25] Finally, because of their superior breadth and potency, VRC01-class bNAbs have been the focus of many atomic-level structural studies revealing the precise paratopes and epitopes of such bNAbs and their gl-bNAb precursors, thereby facilitating structure-based vaccine design.[26,27]

However, VRC01-class bNAbs often require high levels of somatic hypermutation (SHM) to be broad and potent, sometimes approaching 50%.[20,21] Moreover, many VRC01-class bNAbs require rare insertions and/or deletions (indels) for full activity. For example CH31, 3BNC60, and VRC03 contain insertions in the heavy chain (HC) CDRH1 or FWR3, while others, including VRC01, PGV04, and PGV20, require deletions in the LC CDRL1.[28] It has been proposed that highly improbable insertions, such as in the CDRH1 of CH31 or the FWRH3 of 3BNC60, make additional contacts with a neighboring protomer of the Env trimer.[29] In the case of CH31, it only acquired its neutralization breadth after formation of the large (nine-residue) CDRH1 insert, underscoring the impact insertions can have on recognition and neutralization of HIV-1.[28,30] Similarly, CD4bs-targeting bNAbs 1–18 that uses IGHV1-46, the closest related IGHV gene to IGHV1-2, has a six-amino-acid CDRH1 insertion that proved necessary for breadth and potency.[30] Furthermore, VRC01-class bNAbs require small CDRL1 deletions or glycine substitutions, usually in an GXG motif, to accommodate the N276 glycan, a major obstruction to access of the CD4bs.[31,32] Thus, while relatively high levels of SHM can be achieved through vaccination in some circumstances,[4,33] the induction of these rare indels pose a major roadblock to eliciting potent VRC01-class bNAbs by vaccination.

Most Env proteins do not engage VRC01-class bNAb precursors,[11,13] unless specifically modified to do so. The lead vaccine candidates for targeting VRC01-class gl-bNAbs include eOD-GT8 multimerized/60-mer,[34] 426c TM4ΔV1-3,[35] and BG505 SOSIP.v4.1-GT1.1, a derivative of BG505 SOSIP.v4.1-GT1.[36] All three are now in human clinical trials (NCT05414786, NCT05471076, and NCT04224701, respectively). eOD-GT8 primes VRC01-class precursors in various KI mouse models,[34,37–39] as does 426c TM4ΔV1-3.[35] Furthermore, eOD-GT8 can select VRC01-class precursors from the naive B cell repertoire of healthy human donors.[31] eOD-GT8 and 426c TM4ΔV1-3 are based on Env subdomains, either the gp120 outer domain or the gp120 core, respectively. eOD-GT8 and 426c TM4ΔV1-3 have not been reported to be able to select for insertions efficiently, but eOD-GT8 priming did select for CDRL1 deletions.[40,41]

The design of BG505 SOSIP.v4.1-germline-targeting trimer 1 (GT1) using the native-like trimer BG505 SOSIP platform, is rooted in the hypothesis that a native-like trimer might offer advantages over smaller Env fragments by placing the epitope of choice in the natural native-like trimer context. As such, it constrains the approach angles so that they resemble those of the eventual target of bNAbs: the native Env trimer. Therefore, we previously re-engineered the BG505 SOSIP trimer to specifically engage gl-VRC01 as well as V2-apex gl-precursors. However, while the GT1 trimer was able to engage VRC01-class gl-VRC01, gl-PGV19, and gl-NIH45-46 with nanomolar affinity, it was unable to bind to other gl-bNAbs isolated from different human individuals, including gl-3BNC60, gl-12A12, and gl-CH31.[36]

As germline-targeting immunogens are considered to be priming immunogens, it is likely that additional and different immunogens are required to sequentially guide antibody maturation toward neutralization breadth.[16,33,37] Indeed, sequential immunization regimens starting with a germline-targeting immunogen, followed by boosting with shaping and polishing immunogens, including native-like SOSIP trimers, have improved and broadened Env recognition, in one case leading to development of bNAbs in a gl-PGT121 KI mouse model.[19,42] Nevertheless, bNAbs have not yet been consistently induced by vaccination of VRC01-class KI mice with the lead germline-targeting immunogens.[34–39] The major impediment to the development of VRC01-class bNAbs in these models is thought to be the N276 glycan that hinders access to the CD4bs.[37,39] VRC01-class precursors could be initiated and matured into Abs that could neutralize viruses from which the N276 glycan was absent, but not wild-type viruses.[33,35,37,39] However, more recently, select VRC01-class recombinant NAbs have been isolated from KI mice that neutralize N276 glycan-bearing viruses with up to ~50% breadth, albeit with low potency.[41]

Here, we sought to evaluate whether a modified version of the germline targeting GT1 trimer, GT1.2, specifically engineered to expand VRC01-class precursor recognition, could prime a VRC01-class antibody response in a novel gl-CH31 KI mouse model and whether this initial response could be broadened and matured by subsequent immunization with shaping and polishing immunogens. We report that GT1.2 priming followed by these shaping and polishing immunogens resulted in CD4bs-specific serum activity and neutralization of VRC01-signature viruses. This immunization regimen also reproducibly selected for high levels of improbable VRC01-class mutations, including extremely rare multi-residue insertions similar or identical to those observed in VRC01-class bNAbs. Moreover, the vaccination regimen also selected for multi-residue deletions or glycine substitutions in the CDRL1.

RESULTS

Establishment of a gl-CH31 KI mouse model

Although VRC01-class precursor KI mouse models exist, including for gl-VRC01 and gl-3BNC60, they exhibit vastly different B cell phenotypes, despite expressing identical IGHV1-2 gene segments. While gl-3BNC60 KI mice would allow for the study of FWR3 insertions as present in 3BNC60, these models display multiple negative B cell selection controls including peripheral deletion and apoptosis, anergy, and extensive LC editing and swapping, cumulatively indicating significant *in vivo* autoreactivity and profoundly abrogated affinity

maturation.[35] Therefore, we generated CH31 unmutated common ancestor (UCA) double KI mice (i.e., dKI; $V_HDJ_H^{+/+}/VJ^{+/+}$, hereafter referred to as gl-CH31 KI mice) (Figure S1A), whose naive B cells have enforced the IgH/L locus-specific expression of the inferred UCA rearrangements of the CH31-CH34 bNAb lineage with methods that we previously used to engineer other bNAb-lineage UCA-rearranged KI models.[43–45] As opposed to the gl-3BNC60 model and similar to the gl-VRC01 models, gl-CH31 KI mice had largely unperturbed overall B cell development relative to wild-type (WT) C57BL6 controls, including similar developmental subsets (Figure S1B) and comparable surface IgM and IgD BCR densities (Figure S1C) with only a modest decrease in total B cell cellularity (Figure S1D). In contrast with the *in vivo* tolerizing B cell controls observed in gl-3BN60 KI mice, these findings of largely normal B cell development in gl-CH31 KI mice reinforces the notion that CDR3 specificity is crucial in controlling the developmental the fates of VRC01-class B cell precursors.[46] Importantly, this model allows for the study of the ability of vaccine regimens to recapitulate the acquisition of large HC insertions in VRC01-class bNAb lineages.

Finally, to incorporate additional B cell repertoire diversity to this model, we crossed gl-CH31 KI mice with WT C57BL/6 mice, resulting in heterozygous ($V_HDJ_H^{+/-}/V_\kappa J_\kappa^{+/-}$) gl-CH31 KI mice. Because of both this feature (which provides an alternate, unrearranged murine LCκ allele) and the manner in which we have knocked in the CH31 V_κ1-33/Jκ2 rearrangement (Figure S1A), numerous opportunities exist for other LC rearrangements. Indeed, ~45% of endogenous LCs are paired with the gl-CH31 HC (Figure S1F), consistent with the number of non-CD4bs cells we detect by flow cytometry (Figure S1E). In summary, the heterozygous gl-CH31 mouse model is thus suitable to assess the priming potential of a VRC01-class germline-targeting immunogen for its ability to successfully activate gl-CH31$^+$ B cells *in vivo*.

GT1.2 engages gl-CH31 while retaining binding to other gl-bNAbs

To assess the activation of gl-CH31 B cells *in vivo* and to study the selection of indels typical of VRC01-class bNAbs, we designed a trimer that could engage gl-CH31, as the parental BG505 SOSIP GT1 does not engage gl-CH31.[36] Accordingly, we introduced an N279D substitution that establishes an additional contact between GT1 and VRC01-class gl-bNAbs and named the resulting trimer GT1.2 (Figure 1A). The glycan profile of GT1.2 is similar to that of GT1 and is characterized by complex glycans at the apex and trimer base, which may be caused by deletion of glycans leading to localized enhancement of glycan processing (Figures 1A, S2B, and S2C).[36,47] The resulting trimer was well formed and remained in a closed, native-like prefusion conformation comparable with GT1 and other native-like HIV-1 trimers, as assessed by negative stain electron microscopy and gel electrophoresis (Figures 1B and S2A).[36,48] Moreover, its thermal denaturation was also highly comparable with that of GT1 and BG505 SOSIP.664 as evaluated by differential scanning calorimetry, with a melting temperature of 68.0°C (Figure S2D).[36,49]

To assess whether the N279D substitution conferred binding to gl-CH31, we tested GT1.2 binding to different germline precursors and mature bNAbs in a surface plasmon resonance assay (Figure 1C). Whereas no dissociate constant (K_D) could be derived from the weak GT1 binding to gl-CH31, GT1.2 was able to bind gl-CH31 with slightly higher affinity than eOD-GT8 (K_D GT1.2, 4 μM; K_D eOD-GT8, 11.9 μM) (Figures 1C and S2E, Table S1, [34]). Moreover, the introduction of N279D preserved binding of GT1.2 to other gl-bNAbs such as gl-VRC01 (K_D of 1200 nM) and gl-PGV19 (K_D of 94 nM), thus effectively expanding the range of VRC01-class precursors that can be engaged with a single substitution (Figures 1C and S2E, Table S1). This bivalent modeling has been validated[50,51] and used for analyzing the interaction of germline-reverted and mature antibodies with germline-adapted and -unadapted Env trimers.[13,36]

To confirm that the N279D substitution indeed allowed engagement of the conserved W100b, we determined the structure of GT1.2 in complex with VRC01-class precursor gl-PGV20 and PGT124 at 3.8 Å resolution (Figures 1D–1F, Table S2). The structure revealed an additional potential hydrogen bond between $N279D_{GT1.2}$ and $W100b_{gl\text{-}PGV20}$ as hypothesized, reinforcing the GT1.2/gl-PGV20 contacts, and, by inference, gl-CH31 contacts (Figure 1E). Moreover, the overall structure of GT1.2 is highly similar to BG505 SOSIP.664 and GT1, with C_{alpha} root-mean-square deviation values of 0.6 Å (GT1.2 vs. BG505 SOSIP.664) and 0.4 Å (GT1.2 vs. GT1) (Figure 1F). Thus, with the N279D substitution in GT1.2, the range of VRC01-class bNAb precursors that can engage GT1.2 is expanded through an additional conserved W100b contact.

GT1.2 priming induces broadly reactive CD4bs-specific antibody responses

Next, we evaluated GT1.2 as a priming immunogen in the gl-CH31 KI mice described above. In a first experiment, five gl-CH31 KI mice received 25 μg GT1.2 trimer formulated in 60 μg poly I:C adjuvant at weeks 0 and 4 followed by boosting with a fully glycosylated BG505 SOSIP trimer at weeks 7, 13. and 18 (Figure 2A). Serum antibody responses against GT1.2, BG505 SOSIP, a CD4bs knockout (KO) BG505 SOSIP (BG505 D368R), and a candidate shaping immunogen AMC008 GT1 were measured by ELISA[36,52,53] (Figure S3A–S3C). One GT1.2 immunization led to detectable GT1.2-specific responses in all five mice and the additional immunizations strengthened these responses. However, the sera were only weakly reactive to BG505 SOSIP and equally reactive with the BG505 SOSIP D368R mutant that knocks down VRC01-class Ab binding, indicating that antibodies induced by this regimen did not strongly recognize fully glycosylated trimers, and that those that did were not specific for the CD4bs (Figure 2B, left and right). Furthermore, only one mouse displayed neutralization of 426c.TM4, a virus used to gauge VRC01-class neutralization signatures in serum (Figure 2C). The 426c.TM1 virus, which lacks only the N276 glycan, was not neutralized by any of the sera. We concluded that this simple prime-boost regimen using GT1.2 and BG505 SOSIP trimers was insufficient to strongly activate and mature VRC01-class responses in gl-CH31 mice. One implication is that efficient maturation may require shaping immunogens between the priming and polishing stages.

Therefore, we improved the study regimen by selecting affinity-intermediate shaping immunogens in real time, based on the serological reactivity of gl-CH31 KI mice primed with GT1.2

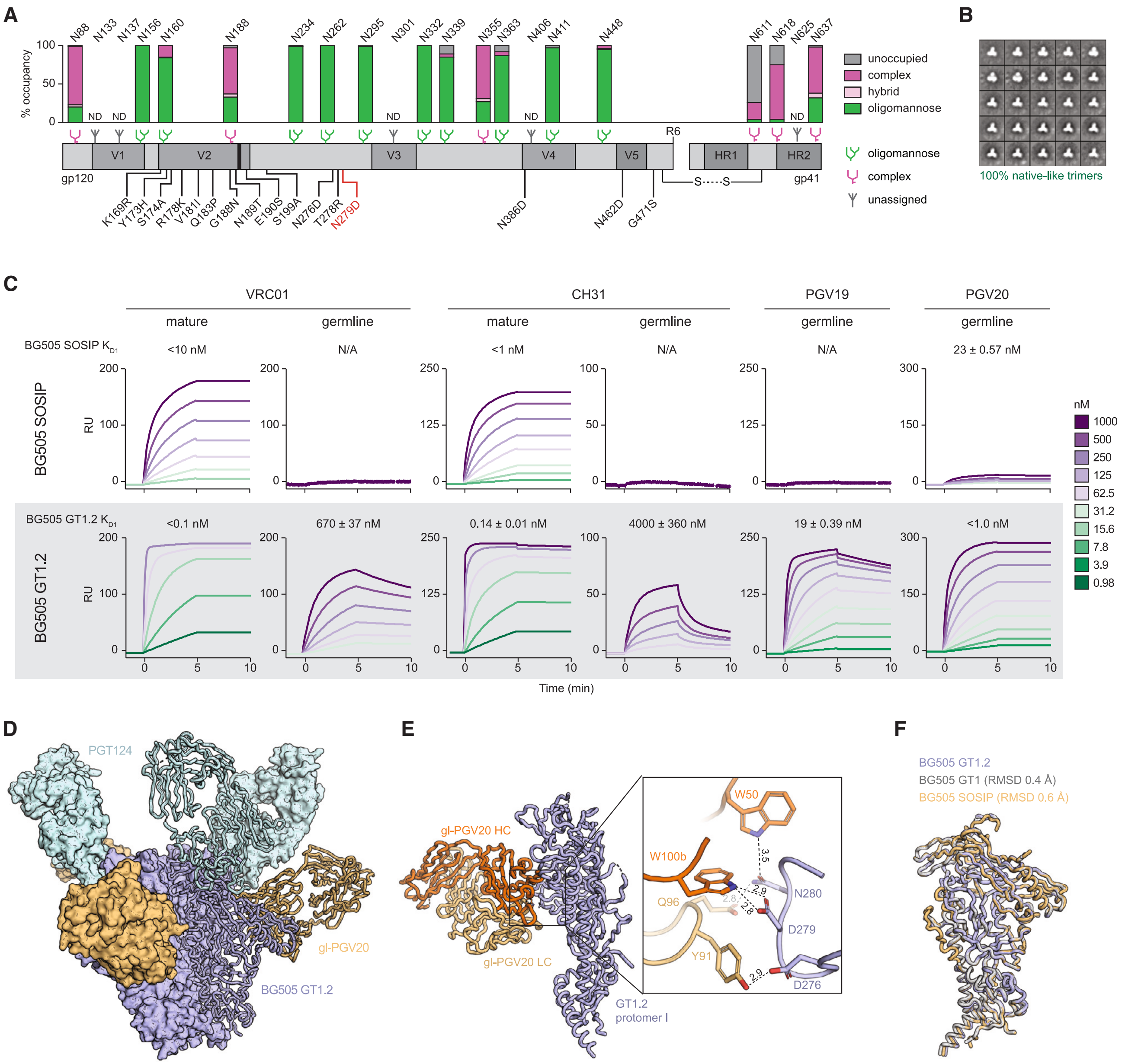

Figure 1. Design and antigenicity of BG505 SOSIP germline trimer 1.2 (GT1.2)

(A) Schematic linear representation of BG505 SOSIP.v4.1 GT1.2 with glycan occupancy data. All amino acid mutations compared with BG505 SOSIP.664 also present in BG505 SOSIPv4.1 GT1 are indicated in black, whereas the N279D substitution defines GT1.2. The glycan icons on top of the linear GT1.2 sequence represent the predominant type of glycan observed at that specific potential N-glycosylation site. ND, not determined.

(B) Negative-stain electron micrograph of soluble GT1.2 trimers.

(C) Surface plasmon resonance sensorgrams showing the specific binding signal in response units (Rus) on the y axes as a function of time during association and dissociation on the x axes for each of the antibody concentrations used (0.98 nM–1,000 nM).

(D) Crystal structure of BG505 SOSIP.v4.1-GT1.2 (blue) trimer in complex with gl-PGV20 (orange) and PGT124 (cyan) Fabs at 3.8 Å resolution.

(E) Side view of the crystal structure of the gl-PGV20 Fab bound to of GT1.2 (blue). (Right) Close-up view of the W100b hydrogen bond interactions of gl-PGV20 with N279 and N280 on GT1.2.

(F) Superimposition of GT1.2 (blue), BG505 SOSIP.664 (orange) (PDB: 5CEZ), and GT1 (gray) (PDB: 5W6D).

(Figure 2D). We also assessed improving the functional affinity of GT1.2 by enhancing avidity through the use of two-component I53-50 protein nanoparticles (NPs) that can display twenty SOSIP trimers.[48,55] We generated GT1.2 I53-50 NPs, which efficiently activated gl-CH31 KI B cells *ex vivo* (Figure S3D), and thus were included in a follow-up study (Figure 2D).

CellPress
OPEN ACCESS

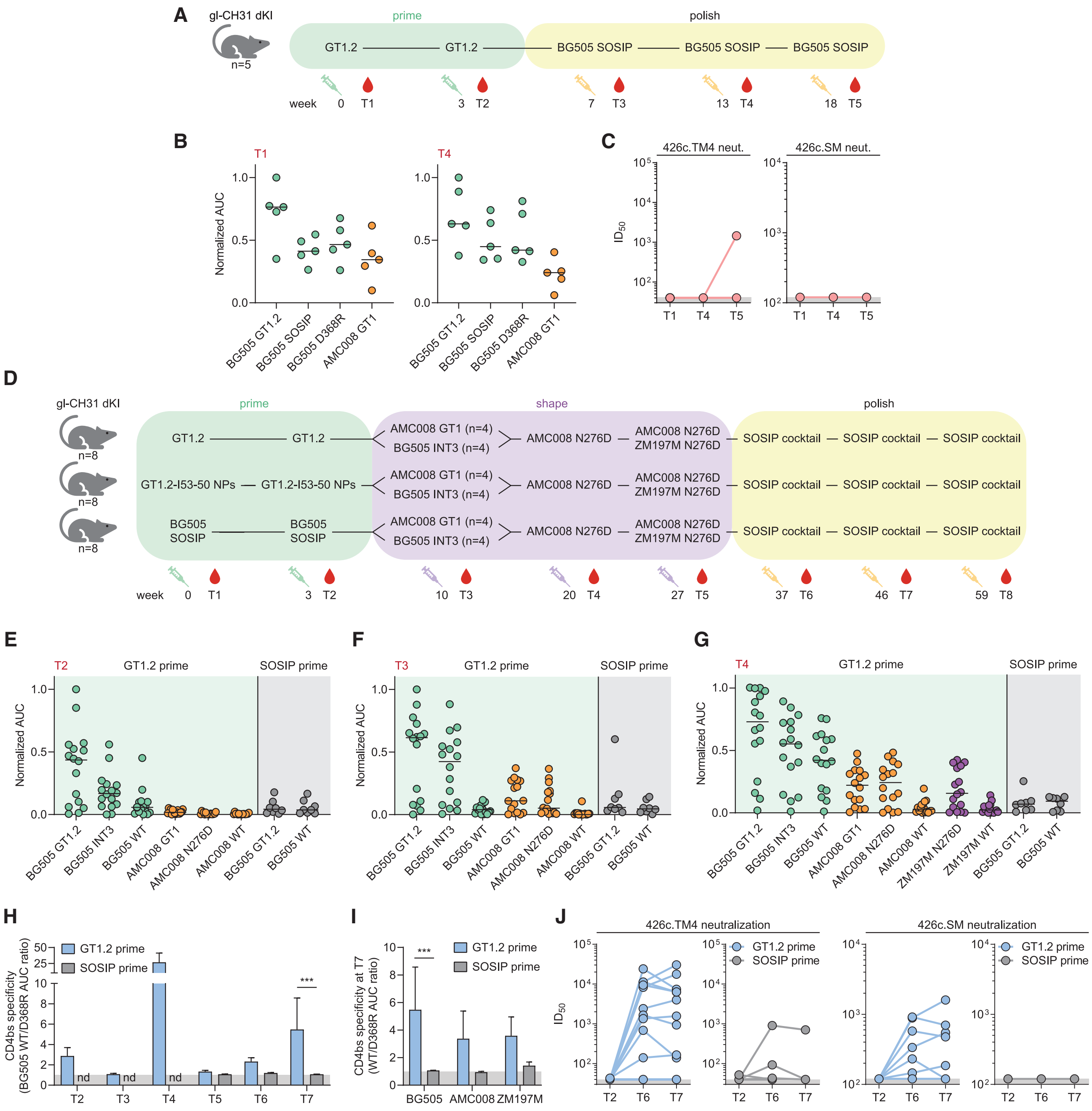

Figure 2. GT1.2 primes CD4bs-directed VRC01-class serum responses in a gl-CH31 KI mouse model
(A) Schematic of the simple prime-polish immunization regimen. The immunizations are indicated by syringes.
(B) Normalized area under the curve (AUC) values of serum antibody binding to the indicated Env as measured by ELISA, for time point T1 (left) and T4 (right).
(C) Midpoint titers (ID_{50}) of serum against VRC01-class signature viruses as described previously (LaBranche et al.[54]) for each time point (T1, T4, and T5).
(D) Schematic of the complex sequential immunization regimen.
(E–G) Normalized AUC values of serum antibody binding to the indicated Env as measured by ELISA, for time point T2 (E), T3 (F), or T4 (G). The background color represents the priming group (GT1.2 trimer/NP vs. BG505).
(H) CD4bs specificity of mice primed with GT1.2 (blue, n = 16) or BG505 (gray, n = 8) for each time point, represented by the BG505/BG505 D368R AUC ratio as measured by ELISA.
(I) CD4bs specificity of mice primed with GT1.2 (blue, n = 16) or BG505 (gray, n = 8) at T7 for each of BG505 (clade A), AMC008 (clade B), or ZM197M (clade C) Envs as in (H).
(J) Midpoint neutralization titers (ID_{50}) of serum from mice primed with GT1.2 (blue, n = 16) or BG505 (gray, n = 8) against VRC01-class signature viruses as in (C) for each time point (T2/T6/T7). Each dot represents an individual mouse.

We primed a new cohort of gl-CH31 KI mice at weeks 0 and 3 with either 25 μg GT1.2 trimer, equimolar amounts of GT1.2-I53-50 NP, or 25 μg BG505 SOSIP trimer as a control (n = 8 per group). BG505 SOSIP is not optimized for germline-targeting and thus should mainly induce off-target, non-CD4bs Ab responses. To compare these priming immunogens, serological reactivity was measured at week 5 by ELISA. The GT1.2 and GT1.2 NP immunized animals developed strong binding antibody responses against GT1.2, although there was considerable variation between animals (Figures 2E and S3E). In contrast, BG505 SOSIP did not induce a strong GT1.2 or BG505 SOSIP response in these mice. We did not observe significant serological differences between the GT1.2 trimer and GT1.2 NP groups, possibly because the avidity advantage of NP presentation does not offer a benefit in the context of high precursor frequency[56,57] (Figure S3E). Therefore, these groups were combined in the analyses below.

GT1.2 priming also induced high binding levels to candidate-shaping immunogen BG505 SOSIP-INT3. INT3 contains only three modifications compared to BG505 SOSIP: N276D, T278R, and a seven-amino-acid deletion in the V2 region, all of which are also present in GT1.2. We also detected weak binding to AMC008 GT1 (Figure 2E). Based on the results, we evenly divided each group into two subgroups and boosted the animals with either BG505 INT3 or AMC008 GT1 with the aim of broadening the CD4bs-directed response and bridging the affinity gap between glycan-deficient GT1.2 and glycan-rich native Envs. This boost indeed strengthened and broadened the serological reactivity; the sera became reactive with AMC008 GT1 and also with AMC008 SOSIP trimers lacking only the N276 glycan (Figure 2F). However, no significant differences were observed between the two different shaping strategies (Figure S3F).

To further boost the breadth of the serum response, all animals across groups were immunized with the AMC008 N276D trimer. This additional boost led to the development of reactivity with a clade C trimer that lacked the N276 glycan, ZM197M N276D, indicative of a further broadening of the response (Figure 2G).[58,59] The sera were now also strongly reactive with unmodified BG505 SOSIP trimers, whereas BG505 SOSIP-primed animals did not develop such binding, suggesting that the shaping immunogens had specifically boosted GT1.2-primed CD4bs-directed antibodies (Figure 2G). We then proceeded to boost all animals with a bivalent cocktail of AMC008 N276D and ZM197M N276D trimers (Figure S3G) before polishing by immunizing thrice with a cocktail of unmodified, native-like trimers from isolates BG505 and Q23 (both clade A) (Figure S3), AMC008 (clade B), and ZM197M and DU422 (both clade C) (Figure 2D and Table S3).[59]

To verify that the serum response was at least partly CD4bs directed, we compared binding to BG505 SOSIP and BG505 SOSIP D368R at each timepoint. Throughout most of this elaborate immunization schedule, the ratio of BG505/BG505 D368R binding was greater than one, indicating the presence of a CD4bs-directed response (Figure 2H). In contrast, BG505 SOSIP-primed animals did not exhibit CD4bs specificity at any stage tested, and the ratio of SOSIP/SOSIP D368R binding was significantly lower after seven immunizations ($p = 0.002$) (Figure 2H). We observed the same trend with D368R trimers of AMC008 and ZM197M, indicating that a broadly reactive CD4bs-directed serum response was generated in the GT1.2-primed animals, but not in the BG505 SOSIP-primed ones (Figure 2I). Finally, a VRC01-class serum neutralization signature was detected in the majority (10/16) of GT1.2-primed animals as measured using the 426c.TM4 virus, whereas only one BG505 SOSIP-primed animal exhibited sustained neutralization of this indicator virus (Figure 2J, left). Moreover, 7 of the 10 GT1.2-primed animals showed 426c.TM4 neutralization, although none of the BG505 SOSIP-primed animals neutralized the 426c.SM virus only lacking the N276 glycan (Figure 2J, right). Taken together, priming with GT1.2, but not unmodified BG505 SOSIP, followed by shaping and polishing, induced a CD4bs-specific serum response in gl-CH31 KI mice.

GT1.2 priming followed by shaping and polishing selects for rare VRC01-class sequence features

The VRC01-class of bNAbs is characterized by high SHM and many of its members also display indels rarely observed in other viral infections.[28] We examined whether the CD4bs-directed serum response in our experiment had any of these features by performing Illumina next-generation sequencing on CD4bs-specific B cells from splenocytes sorted by using eOD-GT8 with negative selection with an eOD-GT8 CD4bs KO mutant from spleens recovered after study completion. eOD-GT8 was chosen as it has a high affinity for gl-VRC01-class bNAbs.[34] We analyzed three groups of immunized mice described previously: mice receiving the short prime-boosting regimen in our first experiment (from here on designated "GT1.2 [short]"), or the longer regimen comprising eight immunizations, primed with either GT1.2 ("GT1.2 [long]"), or BG505 SOSIP ("SOSIP [long]") (Figure 3A). For each of the groups, at least three mice were included. We observed that GT1.2 priming induced significantly more nonsynonymous mutations (≤ 30 amino acids) compared with BG505 SOSIP across Ig subtypes and in IGKV regions (Figure 3B, left, and Figure S4A). However, the median mutation frequency per mouse was significantly higher for GT1.2 (long) than for GT1.2 (short) ($p = 0.0013$), and also higher than for BG505 SOSIP priming (Figure 3B, right). These findings are also reflected in higher sequence diversity for the GT1.2 (long) regimen in 1,000 randomly selected sequences for each mouse (Figure S4B). The mean number of improbable mutations (defined as those having <2% probability in the absence of selection) defined by ARMADiLLO[60] for each mouse was also significantly higher in GT1.2-primed mice with an extended immunization regimen compared to a simple prime-boost regimen (Figure 3C).

We then assessed whether the SHM was on-track and corresponded with mutations found in VRC01-class bNAbs VRC01, CH31, PGV04, PGV20, 3BNC60, and 12A12 (termed VRC01-class mutations). In SOSIP-primed mice, the majority of the sequences recovered had low numbers of mutations and did not exhibit positive selection of VRC01-class mutations. Similar results were obtained with the GT1.2 (short) regimen-administered group (Figure 3D, left and middle). In contrast, sequences recovered from the GT1.2 (long) regimen-administered mice were not only highly mutated but

CellPress
OPEN ACCESS

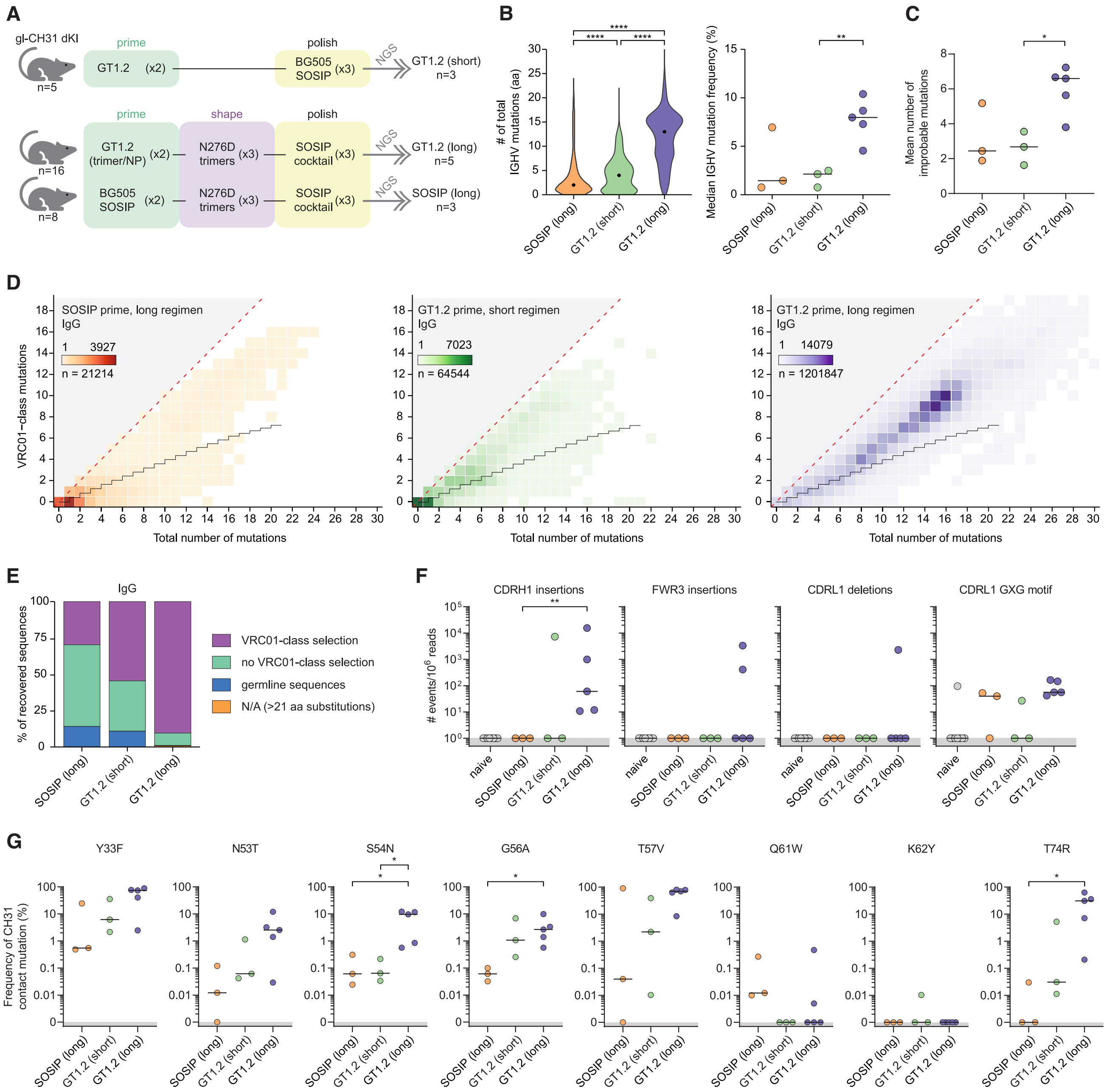

Figure 3. GT1.2 priming but not BG505 priming selects for rare VRC01-class sequence features, including multi-residue insertions and deletions

(A) Simplified representation of the immunization regimen used in Figures 2A–2D.

(B) Violin plot showing the number of total amino acid substitutions in the IGHV region for each group (left) and dot plot showing the median IGHV mutation frequency (%) for each mouse in separate groups (right).

(C) Dot plot showing the mean number of improbable mutations per IGHV region per mouse defined as a <2% probability in the absence of selection.

(D) Total and VRC01-class amino acid mutations in the IGHV1-2 region for recovered IgG sequences for each of the groups as outlined in (A). The staggered black line shows the expected level of VRC01-class mutations as expected to be introduced by random SHM in IGHV1-2 (Briney et al.[37]).

(E) Stacked bar graph showing the frequency of sequences per group selecting for VRC01-class mutations. N/A, not applicable; defined by the limits of the model in Briney et al.[37]

(F) Dot plots showing the frequency of highly infrequent mutational events in recovered sequences. Each dot represents an individual mouse.

(G) Dot plots showing the frequency of substitutions at known CH31 contact residues. Each dot represents an individual mouse. *, $p < 0.05$; **, $p < 0.01$; ****, $p < 0.001$.

revealed a strong selection for VRC01-class mutations, with some sequences harboring up to 19 substitutions found in VRC01-class bNAbs (Figure 3D, right). The accumulation of these high numbers of nonsynonymous VRC01-class mutations is strongly suggestive of sequential cycles of recall of previously expanded B cell clones rather than *de novo* recruitment of naive B cells. Specific selection of VRC01-class mutations was observed in only 29% of BCR sequences from BG505-primed mice, 54% in the group receiving the short GT1.2 regimen, and >90% in that receiving the GT1.2 (long) regimen group (Figure 3E). GT1.2 priming was able to select for the minimal number of VRC01-class mutations necessary for broad and potent neutralization, as that number is 11 and 14 in minimally mutated (min)VRC01 and min12A21, respectively.[61] However, the exact combination of these mutations in minVRC01 and min12A21 are not found in the monoclonal antibodies (mAbs) described here.

Next, we investigated the presence of improbable indel events. We did not observe any CDRH1 insertions in naive mice nor in SOSIP-primed mice (Figure 3F). However, GT1.2-primed mice did show rare CDRH1 insertions, with splenic B cell V(D)J rearrangement sequences from all five mice comprising the GT1.2 (long) group having such insertions ranging in size from four to six amino acids (Figure 3F). Moreover, some GT1.2-primed mice also showed insertions in the FWR3, a region of low mutability because of high frequency of activation-induced cytidine deaminase cold spots.[60] Furthermore, CDRL1 glycine substitutions that follow the VRC01-class GXG motif and that are also thought to drive accommodation of the N276 glycan, were observed in mice in all groups (Figure 3F). We assume these substitutions were induced and/or selected as a result of repeated immunization with N276-containing SOSIPs, not by the GT1.2 priming immunogen, which lacks the N276 glycan. Glycine substitutions were common in the groups that received an eight-immunization regimen but rare in the GT1.2 (short) group. Furthermore, in one mouse in the eight-immunization regimen primed with GT1.2, we observed the second known mechanism of N276 glycan accommodation: a two-amino-acid deletion in the CDRL1 (Figure 3F). We note that mature CH31 itself does not have such a deletion, but has a GXG motif, which may pertain to a preference of the IGHV1-2/IGKV1-33 pairing to resort to GXG motifs as is also observed in IGKV1-33-bearing VRC01-class bNAbs N6 and 12A12.[21,22]

In addition to overall on-track VRC01-class mutations and rare indel events, GT1.2 (long) mice more frequently had specific mutations shared with CH31 that are known to contact the trimer than the other groups (Figure 3G). A number of these mutations, including G56A and T57V, have been implicated previously as key residues in VRC01-class maturation.[20,41] While not all observed frequencies are statistically significantly different, a clear trend is visible where GT1.2 (long) selects for contact mutations more consistently and at up to 1,000-fold higher median frequencies with the exception of Q61W that might rely on N276-glycan exposure (Figure 3G). Thus, an immunization regimen consisting of GT1.2 priming, boosting with shaping immunogens lacking the N276 glycan and polishing with a cocktail of natively glycosylated Envs selects for on-track VRC01-class mutational patterns, including highly infrequent multi-residue indel events and contact mutations shared with CH31 and other VRC01-class bNAbs.

GT1.2 primes antibodies with VRC01-class neutralizing potential

To assess whether these mutational signatures lead to broad binding and possibly neutralization, we sorted single CD4bs-specific eOD-GT8^{+}/eOD-GT8 KO^{-} B cells from five immunized mice in the GT1.2-primed groups that were used in the next-generation sequencing (NGS) analysis (Figure 3). In total, we acquired unique HC/LC paired sequences for 405 B cells, of which 57 were selected and expressed as mAbs based on interesting sequence features and a representative range in SHM, resulting in mAbs A1–A57 (Table S4). Five of 57 mAbs did not show IgG expression and were not analyzed further. The 52 mAbs had 5–17 VRC01-class mutations, consistent with the B cells analyzed by NGS (Figure 3D), and in the range of minVRC01 (12), min12A21 (17), and BG24, a mature VRC01-class bNAb with relatively low SHM (22)[62] (Figure 4A). Apart from accumulating VRC01-class mutations, specific key residues in the HC important for VRC01-class breadth and potency are present in this subset of mAbs,[41] with an average of four of nine key mutations per mAb (Figure 4B). In this representative panel of selected mAbs, some VRC01-class contact residues, such as T57V and Y33 F/V/L/I, were selected in the vast majority of mAbs, which is consistent with the B cells analyzed by NGS (Figure 3G). In contrast, amino acid substitutions at other sites such as V37 were rarely observed (Figure 4B). Seven clonally unrelated mAbs had multi-residue insertions, either in the CDRH1 region or in the FWR3 region (Figure 4C), and 17 of 52 selected mAbs (33%) had a glycine substitution in the CDRL1.

All but two mAbs that expressed efficiently during transient transfection of HEK293F cells showed binding to GT1.2 in a CD4bs-dependent fashion as illustrated by the absence of binding to GT1.2 with two knock out mutations for VRC01-class bNAbs, D279A and D368R (Figure 4D). Most mAbs bound strongly (median effective concentration [EC_{50}] between 0.01 and 0.1 μg/mL) to shaping immunogen AMC008 GT1 and trimers lacking the N276 glycan. However, when the N276 glycan was present, many (31/52) of these mAbs lost the ability to bind ($EC_{50} > 50$ μg/mL), showing that these mAbs had not advanced toward the accommodation of the N276 glycan. However, a proportion (21/52) was able to recognize one or more fully glycosylated native-like trimers.

We tested the ability of these mAbs that bound at least one fully glycosylated trimer to neutralize the autologous viruses. Of these 21 mAbs, 17 neutralized GT1.2 at a half-maximal inhibitory concentration (IC_{50}) below 0.004 μg/mL (Figure 4E, left) and 12 mAbs neutralized BG505 N276D with an IC_{50} of <1 μg/mL. Moreover, a number of mAbs neutralized fully glycosylated native viruses and some neutralized up to five autologous viruses from clades A, B and C, although we note that this neutralization was weak in all cases (10 μg/mL < IC_{50} < 200 μg/mL) (Figure 4E, right). We confirmed that these mAbs target the CD4bs as they potently neutralize the VRC01-class signature viruses 426c.TM1 (N276D/N460D/N463D) and 426c.SM (N276D) but not their CD4bs KO counterparts (N279K) (Figure 4F). Some mAbs showed sporadic neutralization of heterologous viruses

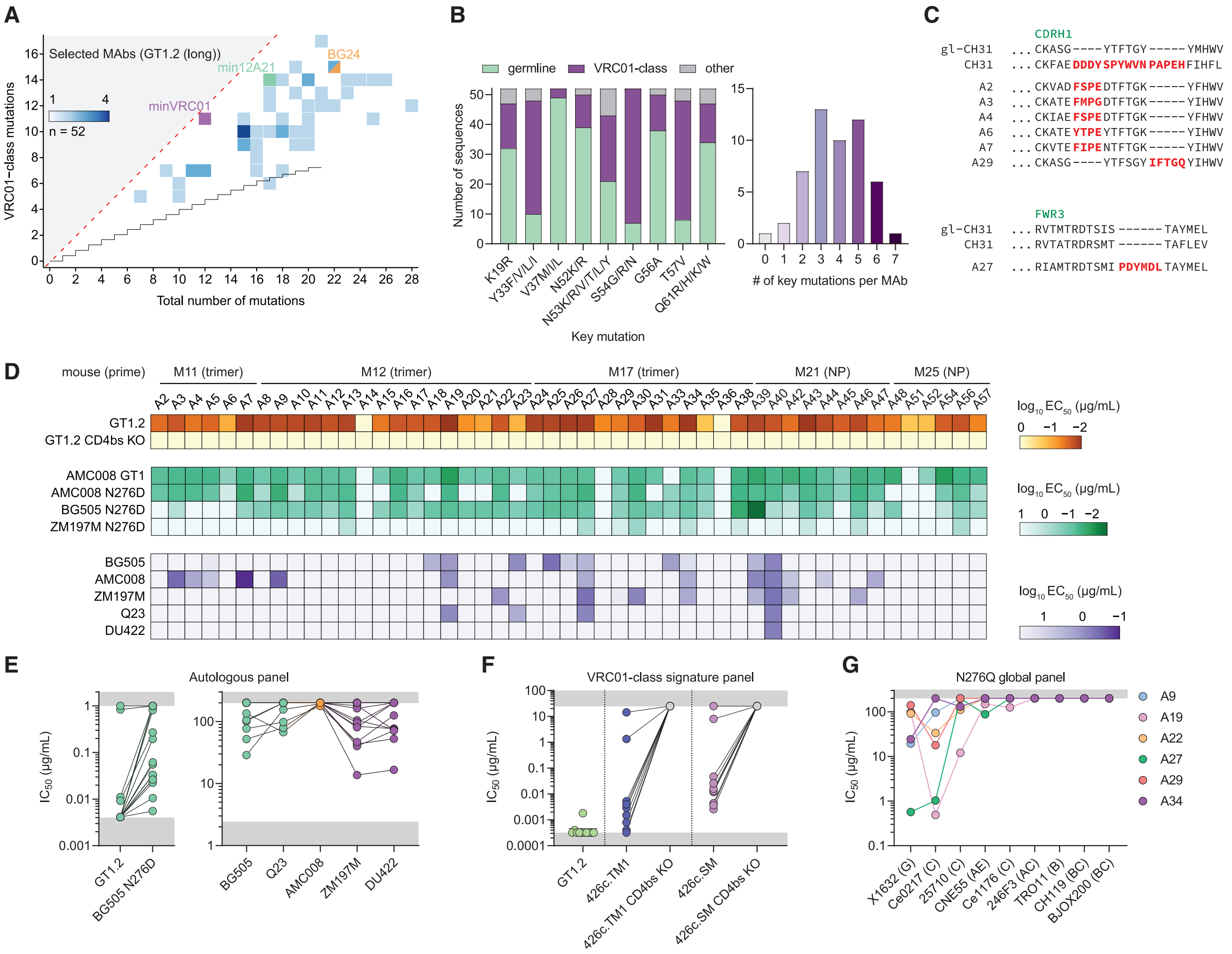

Figure 4. GT1.2-primed VRC01-class mAbs display diverse binding and neutralization capacities

(A) Total and VRC01-class amino acid mutations in the IGHV1-2 region as in Figure 3D for the 52 selected mAbs, including minVRC01 (purple), min12A21 (green), and BG24 (orange).

(B) Stacked bar graph (left) showing whether the residue at the positions indicated on the x axis are germline (green) has mutated into a key VRC01-class residue (purple) or another residue (gray). (Right) Distribution of the number of key mutations in each individual mAb.

(C) Amino acid alignment showing multi-residue insertions (red) in the CDRH1 (top) or in the FWR3 (bottom) of selected mAbs.

(D) Midpoint binding titers (EC_{50}) of each of the selected mAbs. The color corresponds to the starting concentration used (orange, 1 µg/mL; green, 10 µg/mL; purple, 50 µg/mL).

(E) Midpoint neutralization titers (IC_{50}) of a subset of selected mAbs. Each dot corresponds with an individual mAb.

(F) Midpoint neutralization titers (IC_{50}) of a subset of selected mAbs against VRC01-class signature viruses.

(G) Midpoint neutralization titers (IC_{50}) of a subset of selected mAbs against an N276Q global panel as indicated on the x axis.

from the nine-virus global panel when the N276 glycan was removed (N276Q), although no neutralization was observed for the parental viruses (Figure 4G). Thus, although some mAbs have evolved single or double glycines in their CDRL1, the N276 glycan remained a major hurdle in the neutralization of heterologous viruses. The genetic and functional properties of these mAbs collectively suggest that they have progressed substantially on the path from VRC01-class germline precursors to VRC01-class bNAbs and might require few additional mutations to overcome the N276-glycan barrier and acquire neutralization breadth and potency.

Rare indels induced by GT1.2 priming play a major role in antibody binding and neutralization

Although the functional consequences of the two amino acid CDRL1 deletion signature found in many VRC01-class bNAbs are well understood,[32,62] the large CDRH1 and FWR3 insertions have not been the subject of extensive in-depth study. It has been proposed that the CDRH1 insertions of CH31 and 1–18 and the FWR3 insertion of VRC03 and 3BNC60, are involved in establishing contacts with the adjacent Env protomer.[29,30] As far as we know, the mAbs described here are the first reported mAbs with CDRH1 or FWR3 insertions after vaccination.

We tested binding and neutralization of mAbs A7, containing a four amino acid CDRH1 insertion; A23 with a two amino acid deletion in CDRL1; and A27 with a six amino acid insertion in FWR3. Binding to GT1.2 was not negatively impacted by reversion of the respective indel event, with the binding of A23 with full-length CDRL1 in fact being slightly higher (Figure 5A, left), suggesting that the indels were not selected during the initial GT1.2 priming. However, removal of the indel event led to a 2.5-fold and 22-fold decrease in binding to fully glycosylated BG505 SOSIP for A23 and A27, respectively (Figure 5A, middle). However, while A27 shows a similar 15-fold reduction to background levels in binding to AMC008, A7 was not impacted by the removal of its four amino acid insertion (Figure 5A, right). Bio-layer interferometry experiments with BG505 SOSIP confirmed our ELISA findings: A23 showed a modest reduction in binding when the two amino acids in CDRL1 were restored, and A27 showed a large reduction in binding when the six amino acid FWR3 insertion was removed (Figure 5B). These data were further corroborated by neutralization experiments. The neutralization capacity of A27 was severely impacted when the FWR3 insertion was removed, resulting in a failure to neutralize BG505 N276D at IC_{50} of <5 μg/mL and showing a reduction in neutralization potency against native viruses of up to 10-fold (Figures 5C and S5).

Performing a nearest-neighbor search with the A27 HC sequence across all ~250,000 recovered NGS reads from the repertoire of mouse V11417 revealed that the FWR3 insertion in A27 did not arise until significant SHM was achieved, with the closest-related sequence found exhibiting 14 amino acid substitutions, among which are 6 VRC01-class substitutions in the CDRH2 (Figure 5D). Many of these mutations, such as T57V and Q61Y, are contact residues in VRC01-class bNAbs and are highly improbable (Figure 5D). These analyses support the supposition that the insertion was not induced by or selected for by the priming immunogen, but rather during the shaping or polishing phases (Figure 5A).

Finally, to determine whether the A27 FWR3 insertion contributes to antibody-antigen interaction, we used AlphaFold-Multimer to predict the structure of A27.[63] AlphaFold is an artificial intelligence software that attempts to model the structure of a protein based on its amino acid sequence.[64] The AlphaFold-predicted structure of A27 aligns well with experimentally determined structures of VRC01-class bNAbs VRC01, CH31, and 3BNC60, with the variable domains overlapping to a greater extent than the C_H1 and C_L domains of the modeled IgG1 Fab (Figure 5E). Similar to the FWR3 and CDRH1 insertions of 3BNC60 and CH31, respectively, the six inserted residues in the FWR3 of A27 extend to the adjacent gp120 protomer (Figure 5F), possibly allowing hydrogen bonding or salt bridge formation between the $D72_{A27}$ residue and $K207_{gp120}$ on the neighboring protomer as judged from their close proximity. Removal of the inserted residues in A27ΔFWR3 abolishes this interaction, potentially weakening the overall A27-gp120 interactions leading to reduced binding and neutralization. Thus, we show that the incorporation of highly improbable insertions and deletions during vaccination is not simply a byproduct of high SHM, but a selection-driven mechanism to improve engagement of natively glycosylated HIV-1 Env trimers.

DISCUSSION

While germline targeting is a promising vaccination strategy for bNAb induction, it will be challenging to recapitulate how bNAbs naturally develop in some HIV-1-infected people. Specifically, developing VRC01-class NAbs with appropriate breadth and potency requires the initial selection of BCRs with a specific genetic signature, adaptation to glycans surrounding the CD4bs, and high levels of SHM that frequently involve rare indels.[28,30] We show here that gl-CH31 KI mice are an excellent animal model for testing whether vaccination can recapitulate, and perhaps even provide a shortcut for, the complex sequence of events that can occur during chronic infection.

Eliciting VRC01-class bNAbs will likely require carefully designed immunization regimens. A priming immunogen should expand genetically favorable B cell populations, followed by shaping and/or polishing immunogens that gradually expose these B cells to glycan barriers and increasingly diverse CD4bs epitopes. While some designs focus on activating large numbers of B cells through a high-affinity interaction between naive B cell and immunogens, others aim to impose structural constraints early on, expanding lower numbers of B cells, yet imposing a more stringent initial selection. While priming immunogens aim to engage precursor B cells by removing CD4bs-adjacent glycans, boosting immunogens need to gradually impose structural constraints that guide favorable SHM while maintaining the ability to activate primed B cells. We observed no specific CD4bs-directed serum neutralization signature after priming with GT1.2 and boosting with fully glycosylated BG505 SOSIP. Hence, the affinity gap between GT1.2 and a fully glycosylated native Env may be too large, requiring boosting with immunogens lacking one or more glycans to enable GT1.2-primed B cells to continue VRC01-class development.

Although we observed N276 glycan-coping mechanisms present in bNAbs (i.e., CDRL1 deletions and/or glycine substitutions) (Figure 3F), the N276 glycan still poses a major hurdle to the development of NAb breadth, possibly indicating a need for earlier introduction of Env immunogens carrying this glycan, either in native complex form or in shorter Man_5 isoforms.[54] However, the frequent observation of N276 glycan-coping mechanisms in our NGS dataset is encouraging and provides proof of concept that adaptation to the N276 glycan by sequential vaccination is possible.

Recent studies have highlighted the possibility of naturally arising VRC01-class bNAb lineages with relatively low SHM (10%–14% in the HC).[62,65] Other than their relatively low SHM levels, they exhibit canonical VRC01-class signatures, such as IGHV1-2*02 use, a CDRH3 W100b motif and classical N276 glycan-coping strategies. One example is BG24, a VRC01-class bNAb with 13.4% SHM at the nucleotide level that evolved a six amino acid CDRL1 deletion despite relatively low SHM.[62] A second is the PCIN63 lineage with 10%–15% SHM, where a GXG motif emerged to accommodate the N276 glycan.[65] Here, we demonstrate that the SHM levels observed in these VRC01-class bNAbs, as well as the extreme selection pressure coinciding with indel development can be reproducibly achieved through sequential vaccination, but requires priming with germline-targeting immunogen GT1.2. A similar study that used eOD-GT8

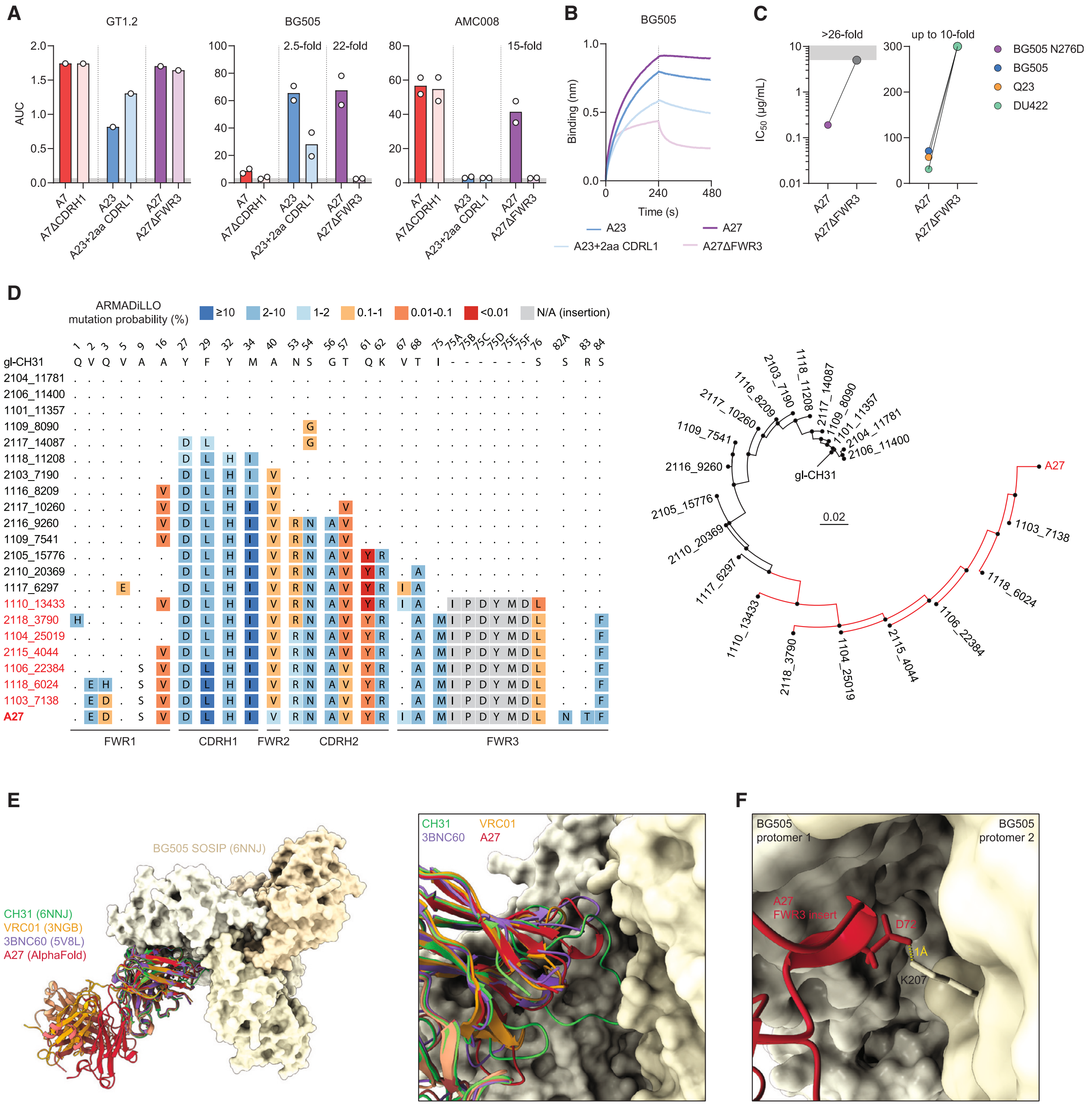

Figure 5. Rare insertions and deletions are important for mAb-Env interactions and might establish quaternary contacts

(A) ELISA binding to Envs expressed as the area under the curve (AUC) for each of the original mAbs and mAbs that had their specific indel removed. Each dot represents an individual experiment.

(B) Bio-layer interferometry (BLI) sensorgrams showing binding of A23 (blue) and A27 (purple) with their indel events removed.

(C) Midpoint neutralization titers (IC_{50}) of A27 and A27ΔFWR3 against the viruses indicated.

(D) Amino acid sequence alignment with ARMADiLLO mutation probabilities (left) and phylogenetic tree (right) of an A27 lineage reconstructed by identifying the shortest path between gl-CH31 and A27 using NGS repertoire reads from the mouse from which A27 was isolated.

(E) Structural representation (top view) of a BG505 SOSIP Env (PDB: 6NNJ) in complex with bNAbs CH31 (PDB: 6NNJ), VRC01 (PDB: 3NGB), 3BNC60 (PDB: 5VBL), and an AlphaFold2-Multimer-modeled structure of A27.

(F) Side view of residue $D72_{A27}$ in the FWR3 insertion extending toward $K207_{gp120}$.

as a priming immunogen showed isolation of NAbs that neutralize heterologous N276 glycan-bearing viruses with up to 54% breadth, albeit at low potency.[41] Although the numbers of immunizations and the animal models used are similar, no B cell clones with multi-residue insertions were isolated after eOD-GT8 priming. Hence, priming, shaping and polishing with native-like SOSIP trimers that impose appropriate steric constraints might be particularly favorable for the selection of such insertions.

We need to understand how and when CDRH1 and FWR3 insertions arise in our gl-CH31 KI immunization model. For instance, given that our existing regimen can reproducibly induce CDRH1 insertions that potentially offer a shortcut compared with those accumulating multiple single residue changes,[29] it will be key to understand if and how they can be acquired through vaccination earlier. We also need to investigate whether the full nine amino acid insertion found in mature CH31 offers advantages over the four amino acid insertion found in this study. Additionally, gaining insight into the molecular mechanisms involved (i.e., V(D)J recombination-related, SHM-associated, both and/or other) may allow for developing approaches to modulate their promotion during vaccination. Considering the disfavoring of indel formation over base substitutions during SHM, these results indicate that strong selection pressure induced through trimer-based sequential immunizations can reproducibly elicit these rare events, which in turn also suggests it drives the same memory B cells to re-enter germinal centers for further rounds of SHM and affinity maturation.

The eight-dose regimen used here would be extremely hard to implement in humans. However, several factors could simplify the design of a practical vaccine regimen. For instance, are two primes and/or three finishing boosts truly required, or might fewer be needed? Additionally, to further increase the chances of translating these approaches in humans, several innovative methods have shown promise in animal models. For example, adoptive transfer experiments with our gl-CH31 model can be used to better mimic low gl-bNAb frequencies, similar to those typically found in human repertoires. Second, osmotic pumps that release small amounts of antigen over time have been shown to result in more robust T follicular helper cell development, germinal center B cells with increased Env affinity and up to 20-fold higher NAb titers in animal models.[66] The simultaneous presentation of different immunogens on a mosaic NP has been shown to increase breadth in the context of SARS-CoV-2 and influenza vaccination.[67–69] This technique may allow multiple germline-targeting phases (i.e., priming-shaping or shaping-polishing) to be triggered by a single immunogen, effectively decreasing the number of immunizations needed to achieve serum breadth. Our current regimen thus holds substantial near-term promise and a strong basis for further iterative pursuit of a truly practical trimer-based strategy, especially given the (in this study) unexplored effects of optimal timing, adjuvanting, and delivery platforms.

Overall, this proof-of-concept study demonstrates that priming with a prefusion-stabilized germline-targeting SOSIP trimer can drive the maturation of VRC01-class Abs, including the selection of multi-residue insertions and the induction of key VRC01-class mutations. We show that these indels are functional, possibly stabilizing the interaction between antigen and antibody, in particular in the context of natively glycosylated trimers. These observations, together with other recent studies showing the elicitation of heterologous NAbs in lower-bar animal models,[33,41] pave the way to develop more feasible, potent, and broad vaccine strategies to elicit anti-HIV-1 bNAbs in humans.

Limitations of this study

Immunization-driven VRC01-class affinity maturation, including functionally important indel formation, represents an important step forward in HIV-1 vaccine research and a conceptually novel avenue in creating universal vaccines to diverse pathogens. However, the gl-CH31 precursor frequency in the relatively low-bar KI mouse model used here is far from the frequency present in a human population.[22,25] Indeed, precursor frequency modulation through adoptive transfer of KI B cells (and/or use of KI mice with fully humanized Ig loci) to achieve near-physiological levels of gl-bNAb precursors will thus prove very informative. Moreover, the total numbers of mice used are suboptimal to draw conclusions from on a population level, even though statistical significance was achieved for many readouts (Figures 2 and 3). Finally, the fact that this first GT1.2 priming vaccine protocol described here through iterative serologic screening does not induce the exact (full-length) nine-amino acid CDRH1 insertion acquired during original human CH30-34 bNAb maturation is also a potential limitation of the study. Nevertheless, it encouraging that the insertions our vaccine protocol produced are in the exact same location in CDRH1 as the one that occurred in CH31 in the patient. Thus, optimization of timing, adjuvanting, and delivery platform modalities, coupled with a deeper basic understanding of vaccine-driven indel formation, should help to improve our current regimen.

STAR★METHODS

Detailed methods are provided in the online version of this paper and include the following:

- KEY RESOURCES TABLE
- RESOURCE AVAILABILITY
  - Lead contact
  - Materials availability
  - Data and code availability
- EXPERIMENTAL MODEL AND SUBJECT DETAILS
  - Mice
  - Cell lines
- METHOD DETAILS
  - Immunizations
  - B cell phenotypic analysis by flow cytometry
  - Env design and characterization
  - Glycopeptide analysis by LC-MS
  - Ultra-high performance liquid chromatography (UPLC) of released glycans
  - Negative-stain electron microscopy
  - Surface plasmon resonance (SPR)
  - Structural analysis
  - Calcium flux analysis

CellPress
OPEN ACCESS

- ○ Immunizations
- ○ Serum enzyme-linked immunosorbent assay (ELISA) assays
- ○ Serum neutralization assays
- ○ Illumina next-generation sequencing (NGS)
- ○ Processing of NGS sequence data
- ○ Sorting of single CD4bs-specific memory B cells
- ○ Isolation of mouse antibody genes by single cell PCR
- ○ Monoclonal antibody (MAb) production and characterization
- QUANTIFICATION AND STATISTICAL ANALYSIS

SUPPLEMENTAL INFORMATION

Supplemental information can be found online at https://doi.org/10.1016/j.xcrm.2023.101003.

ACKNOWLEDGMENTS

We are grateful to the staff of Advanced Photon Source BL 23-IDD for assistance. GM/CA@APS has been funded by the National Cancer Institute (ACB-12002) and the National Institute of General Medical Sciences (AGM-12006, P30GM138396). This research used resources of the Advanced Photon Sciences under contract no. DE-AC02-06CH11357. This work is supported by the Netherlands Organisation for Scientific Research (N.W.O.) Vici grant (R.W.S.); Bill & Melinda Gates Foundation, Collaboration for AIDS Vaccine Discovery (C.A.V.D.) grants INV-002022 (R.W.S.) and OPP1115782/INV-002916 (A.B.W.); Fondation Dormeur, Vaduz (R.W.S.); and grants from the NIAID, Division of AIDS, NIH UM1 grants for the Duke Center for HIV/AIDS Vaccine Immunology-Immunogen Discovery (CHAVI-ID; UM1530AI100645 and Consortia for HIV/AIDS Vaccine Development [CHAVD]) UM1AI144371 to B.F.H. and R01 grant AI087202 (L.K.V.). P.J.K. and J.P.M. are supported by R01 AI036082 and A.B.W., I.A.W., R.W.S., P.J.K., and J.P.M. are supported by a HIVRAD P01 AI110657 grant. M.C. is funded by the International AIDS Vaccine Initiative (IAVI) through grant INV-008352 and the Bill & Melinda Gates Foundation OPP1153692. Funding for the neutralization assays was provided by NIH/NIAID contract #HHSN272201800004C.

AUTHOR CONTRIBUTIONS

Conceptualization: M.M.-R., R.D., B.F.H., L.W., L.K.V., and R.W.S.; investigation: T.G.C., M.M.-R., J.Z., A.S., A.S.K., A.L., R.D., J.A., J.L.S., J.C.P., I.d.M.S., A.Y., M.D., Y.A., T.P.L.B., S.V., J.M.B., A.N., C.J., W.-H.L., M.P., J.A.B., M.J.v.B., and P.J.K.; methodology: T.G.C., M.M.-R., J.Z., A.S., S.K., M.P., M.C., P.J.K., I.A.W., K.W., L.K.V., and R.W.S.; project administration: T.G.C., M.M.-R., K.W., L.K.V., and R.W.S.; supervision: C.L., D.M., G.O., A.B.W., M.C., J.P.M., P.J.K., B.F.H., I.A.W., K.W., L.K.V., and R.W.S.; writing – original draft: T.G.C., L.K.V., and R.W.S.; review and editing: all authors.

DECLARATION OF INTERESTS

Amsterdam UMC has filed a patent application related to germline-targeting HIV-1 Env trimers.

Received: September 15, 2022
Revised: January 23, 2023
Accepted: March 17, 2023
Published: April 11, 2023

REFERENCES

1. Hraber, P., Seaman, M.S., Bailer, R.T., Mascola, J.R., Montefiori, D.C., and Korber, B.T. (2014). Prevalence of broadly neutralizing antibody responses during chronic HIV-1 infection. AIDS *28*, 163–169.
2. Doria-Rose, N.A., Klein, R.M., Daniels, M.G., O'Dell, S., Nason, M., Lapedes, A., Bhattacharya, T., Migueles, S.A., Wyatt, R.T., Korber, B.T., et al. (2010). Breadth of human immunodeficiency virus-specific neutralizing activity in sera: clustering analysis and association with clinical variables. J. Virol. *84*, 1631–1636.
3. Julg, B., Liu, P.-T., Wagh, K., Fischer, W.M., Abbink, P., Mercado, N.B., Whitney, J.B., Nkolola, J.P., McMahan, K., Tartaglia, L.J., et al. (2017). Protection against a mixed SHIV challenge by a broadly neutralizing antibody cocktail. Sci. Transl. Med. *9*, eaao4235. https://doi.org/10.1126/scitranslmed.aao4235.
4. Sok, D., and Burton, D.R. (2018). Recent progress in broadly neutralizing antibodies to HIV. Nat. Immunol. *19*, 1179–1188.
5. Scheid, J.F., Horwitz, J.A., Bar-On, Y., Kreider, E.F., Lu, C.-L., Lorenzi, J.C.C., Feldmann, A., Braunschweig, M., Nogueira, L., Oliveira, T., et al. (2016). HIV-1 antibody 3BNC117 suppresses viral rebound in humans during treatment interruption. Nature *535*, 556–560.
6. Corey, L., Gilbert, P.B., Juraska, M., Montefiori, D.C., Morris, L., Karuna, S.T., Edupuganti, S., Mgodi, N.M., deCamp, A.C., Rudnicki, E., et al. (2021). Two randomized trials of neutralizing antibodies to prevent HIV-1 acquisition. N. Engl. J. Med. *384*, 1003–1014.
7. Barouch, D.H. (2008). Challenges in the development of an HIV-1 vaccine. Nature *455*, 613–619.
8. Bekker, L.-G., Tatoud, R., Dabis, F., Feinberg, M., Kaleebu, P., Marovich, M., Ndung'u, T., Russell, N., Johnson, J., Luba, M., et al. (2020). The complex challenges of HIV vaccine development require renewed and expanded global commitment. Lancet *395*, 384–388.
9. Klasse, P.J., Ozorowski, G., Sanders, R.W., and Moore, J.P. (2020). Env exceptionalism: why are HIV-1 Env glycoproteins atypical immunogens? Cell Host Microbe *27*, 507–518.
10. Liao, H.-X., Lynch, R., Zhou, T., Gao, F., Alam, S.M., Boyd, S.D., Fire, A.Z., Roskin, K.M., Schramm, C.A., Zhang, Z., et al. (2013). Co-evolution of a broadly neutralizing HIV-1 antibody and founder virus. Nature *496*, 469–476.
11. Xiao, X., Chen, W., Feng, Y., Zhu, Z., Prabakaran, P., Wang, Y., Zhang, M.-Y., Longo, N.S., and Dimitrov, D.S. (2009). Germline-like predecessors of broadly neutralizing antibodies lack measurable binding to HIV-1 envelope glycoproteins: implications for evasion of immune responses and design of vaccine immunogens. Biochem. Biophys. Res. Commun. *390*, 404–409.
12. Hoot, S., McGuire, A.T., Cohen, K.W., Strong, R.K., Hangartner, L., Klein, F., Diskin, R., Scheid, J.F., Sather, D.N., Burton, D.R., and Stamatatos, L. (2013). Recombinant HIV envelope proteins fail to engage germline versions of anti-CD4bs bNAbs. PLoS Pathog. *9*, e1003106.
13. Sliepen, K., Medina-Ramírez, M., Yasmeen, A., Moore, J.P., Klasse, P.J., and Sanders, R.W. (2015). Binding of inferred germline precursors of broadly neutralizing HIV-1 antibodies to native-like envelope trimers. Virology *486*, 116–120.
14. McGuire, A.T., Dreyer, A.M., Carbonetti, S., Lippy, A., Glenn, J., Scheid, J.F., Mouquet, H., and Stamatatos, L. (2014). HIV antibodies. Antigen modification regulates competition of broad and narrow neutralizing HIV antibodies. Science *346*, 1380–1383.
15. McGuire, A.T., Glenn, J.A., Lippy, A., and Stamatatos, L. (2014). Diverse recombinant HIV-1 Envs fail to activate B cells expressing the germline B cell receptors of the broadly neutralizing anti-HIV-1 antibodies PG9 and 447-52D. J. Virol. *88*, 2645–2657.
16. Haynes, B.F., Kelsoe, G., Harrison, S.C., and Kepler, T.B. (2012). B-cell-lineage immunogen design in vaccine development with HIV-1 as a case study. Nat. Biotechnol. *30*, 423–433.
17. Medina-Ramírez, M., Sanders, R.W., and Klasse, P.J. (2014). Targeting B-cell germlines and focusing affinity maturation: the next hurdles in HIV-1-vaccine development? Expert Rev. Vaccines *13*, 449–452.
18. Stamatatos, L., Pancera, M., and McGuire, A.T. (2017). Germline-targeting immunogens. Immunol. Rev. *275*, 203–216.

19. Escolano, A., Steichen, J.M., Dosenovic, P., Kulp, D.W., Golijanin, J., Sok, D., Freund, N.T., Gitlin, A.D., Oliveira, T., Araki, T., et al. (2016). Sequential immunization elicits broadly neutralizing anti-HIV-1 antibodies in Ig knockin mice. Cell *166*, 1445–1458.e12.

20. Zhou, T., Georgiev, I., Wu, X., Yang, Z.-Y., Dai, K., Finzi, A., Kwon, Y.D., Scheid, J.F., Shi, W., Xu, L., et al. (2010). Structural basis for broad and potent neutralization of HIV-1 by antibody VRC01. Science *329*, 811–817.

21. Scheid, J.F., Mouquet, H., Ueberheide, B., Diskin, R., Klein, F., Oliveira, T.Y.K., Pietzsch, J., Fenyo, D., Abadir, A., Velinzon, K., et al. (2011). Sequence and structural convergence of broad and potent HIV antibodies that mimic CD4 binding. Science *333*, 1633–1637.

22. Huang, J., Kang, B.H., Ishida, E., Zhou, T., Griesman, T., Sheng, Z., Wu, F., Doria-Rose, N.A., Zhang, B., McKee, K., et al. (2016). Identification of a CD4-binding-site antibody to HIV that evolved near-Pan neutralization breadth. Immunity *45*, 1108–1121.

23. Sajadi, M.M., Dashti, A., Rikhtegaran Tehrani, Z., Tolbert, W.D., Seaman, M.S., Ouyang, X., Gohain, N., Pazgier, M., Kim, D., Cavet, G., et al. (2018). Identification of near-pan-neutralizing antibodies against HIV-1 by deconvolution of plasma humoral responses. Cell *173*, 1783–1795.e14.

24. Bonsignori, M., Montefiori, D.C., Wu, X., Chen, X., Hwang, K.-K., Tsao, C.-Y., Kozink, D.M., Parks, R.J., Tomaras, G.D., Crump, J.A., et al. (2012). Two distinct broadly neutralizing antibody specificities of different clonal lineages in a single HIV-1-infected donor: implications for vaccine design. J. Virol. *86*, 4688–4692.

25. Lee, J.H., Toy, L., Kos, J.T., Safonova, Y., Schief, W.R., Havenar-Daughton, C., Watson, C.T., and Crotty, S. (2021). Vaccine genetics of IGHV1-2 VRC01-class broadly neutralizing antibody precursor naïve human B cells. NPJ Vaccines *6*, 113.

26. Dam, K.-M.A., Barnes, C.O., Gristick, H.B., Schoofs, T., Nussenzweig, M.C., and Bjorkman, P.J. (2022). HIV-1 CD4-binding site germline antibody–Env structures inform vaccine design. Preprint at bioRxiv. https://doi.org/10.1101/2022.03.25.485873.

27. Derking, R., and Sanders, R.W. (2021). Structure-guided envelope trimer design in HIV-1 vaccine development: a narrative review. J. Int. AIDS Soc. *24*, e25797.

28. Kepler, T.B., Liao, H.-X., Alam, S.M., Bhaskarabhatla, R., Zhang, R., Yandava, C., Stewart, S., Anasti, K., Kelsoe, G., Parks, R., et al. (2014). Immunoglobulin gene insertions and deletions in the affinity maturation of HIV-1 broadly reactive neutralizing antibodies. Cell Host Microbe *16*, 304–313.

29. Kepler, T.B., and Wiehe, K. (2017). Genetic and structural analyses of affinity maturation in the humoral response to HIV-1. Immunol. Rev. *275*, 129–144.

30. Schommers, P., Gruell, H., Abernathy, M.E., Tran, M.-K., Dingens, A.S., Gristick, H.B., Barnes, C.O., Schoofs, T., Schlotz, M., Vanshylla, K., et al. (2020). Restriction of HIV-1 escape by a highly broad and potent neutralizing antibody. Cell *180*, 471–489.e22.

31. Jardine, J.G., Kulp, D.W., Havenar-Daughton, C., Sarkar, A., Briney, B., Sok, D., Sesterhenn, F., Ereño-Orbea, J., Kalyuzhniy, O., Deresa, I., et al. (2016). HIV-1 broadly neutralizing antibody precursor B cells revealed by germline-targeting immunogen. Science *351*, 1458–1463.

32. Kong, L., Ju, B., Chen, Y., He, L., Ren, L., Liu, J., Hong, K., Su, B., Wang, Z., Ozorowski, G., et al. (2016). Key gp120 glycans pose roadblocks to the rapid development of VRC01-class antibodies in an HIV-1-Infected Chinese donor. Immunity *44*, 939–950.

33. Lee, J.H., Nakao, C., Appel, M., Le, A., Landais, E., Kalyuzhniy, O., Hu, X., Liguori, A., Mullen, T.-M., Groschel, B., et al. (2022). Highly mutated antibodies capable of neutralizing N276 glycan-deficient HIV after a single immunization with an Env trimer. Cell Rep. *38*, 110485.

34. Jardine, J.G., Ota, T., Sok, D., Pauthner, M., Kulp, D.W., Kalyuzhniy, O., Skog, P.D., Thinnes, T.C., Bhullar, D., Briney, B., et al. (2015). HIV-1 VACCINES. Priming a broadly neutralizing antibody response to HIV-1 using a germline-targeting immunogen. Science *349*, 156–161.

35. McGuire, A.T., Gray, M.D., Dosenovic, P., Gitlin, A.D., Freund, N.T., Petersen, J., Correnti, C., Johnsen, W., Kegel, R., Stuart, A.B., et al. (2016). Specifically modified Env immunogens activate B-cell precursors of broadly neutralizing HIV-1 antibodies in transgenic mice. Nat. Commun. *7*, 10618.

36. Medina-Ramírez, M., Garces, F., Escolano, A., Skog, P., de Taeye, S.W., Del Moral-Sanchez, I., McGuire, A.T., Yasmeen, A., Behrens, A.-J., Ozorowski, G., et al. (2017). Design and crystal structure of a native-like HIV-1 envelope trimer that engages multiple broadly neutralizing antibody precursors in vivo. J. Exp. Med. *214*, 2573–2590.

37. Briney, B., Sok, D., Jardine, J.G., Kulp, D.W., Skog, P., Menis, S., Jacak, R., Kalyuzhniy, O., de Val, N., Sesterhenn, F., et al. (2016). Tailored immunogens Direct affinity maturation toward HIV neutralizing antibodies. Cell *166*, 1459–1470.e11.

38. Dosenovic, P., von Boehmer, L., Escolano, A., Jardine, J., Freund, N.T., Gitlin, A.D., McGuire, A.T., Kulp, D.W., Oliveira, T., Scharf, L., et al. (2015). Immunization for HIV-1 broadly neutralizing antibodies in human Ig knockin mice. Cell *161*, 1505–1515.

39. Tian, M., Cheng, C., Chen, X., Duan, H., Cheng, H.-L., Dao, M., Sheng, Z., Kimble, M., Wang, L., Lin, S., et al. (2016). Induction of HIV neutralizing antibody lineages in mice with diverse precursor repertoires. Cell *166*, 1471–1484.e18.

40. Huang, D., Abbott, R.K., Havenar-Daughton, C., Skog, P.D., Al-Kolla, R., Groschel, B., Blane, T.R., Menis, S., Tran, J.T., Thinnes, T.C., et al. (2020). B cells expressing authentic naive human VRC01-class BCRs can be recruited to germinal centers and affinity mature in multiple independent mouse models. Proc. Natl. Acad. Sci. USA *117*, 22920–22931.

41. Chen, X., Zhou, T., Schmidt, S.D., Duan, H., Cheng, C., Chuang, G.-Y., Gu, Y., Louder, M.K., Lin, B.C., Shen, C.-H., et al. (2021). Vaccination induces maturation in a mouse model of diverse unmutated VRC01-class precursors to HIV-neutralizing antibodies with >50% breadth. Immunity *54*, 324–339.e8.

42. Steichen, J.M., Kulp, D.W., Tokatlian, T., Escolano, A., Dosenovic, P., Stanfield, R.L., McCoy, L.E., Ozorowski, G., Hu, X., Kalyuzhniy, O., et al. (2016). HIV vaccine design to target germline precursors of glycan-dependent broadly neutralizing antibodies. Immunity *45*, 483–496.

43. Zhang, R., Verkoczy, L., Wiehe, K., Munir Alam, S., Nicely, N.I., Santra, S., Bradley, T., Pemble, C.W., Zhang, J., Gao, F., et al. (2016). Initiation of immune tolerance-controlled HIV gp41 neutralizing B cell lineages. Sci. Transl. Med. *8*, 336ra62.

44. Williams, W.B., Zhang, J., Jiang, C., Nicely, N.I., Fera, D., Luo, K., Moody, M.A., Liao, H.-X., Alam, S.M., Kepler, T.B., et al. (2017). Initiation of HIV neutralizing B cell lineages with sequential envelope immunizations. Nat. Commun. *8*, 1732–1820.

45. Saunders, K.O., Wiehe, K., Tian, M., Acharya, P., Bradley, T., Alam, S.M., Go, E.P., Scearce, R., Sutherland, L., Henderson, R., et al. (2019). Targeted selection of HIV-specific antibody mutations by engineering B cell maturation. Science *366*, eaay7199. https://doi.org/10.1126/science.aay7199.

46. Verkoczy, L., Alt, F.W., and Tian, M. (2017). Human Ig knockin mice to study the development and regulation of HIV-1 broadly neutralizing antibodies. Immunol. Rev. *275*, 89–107.

47. Behrens, A.-J., Kumar, A., Medina-Ramirez, M., Cupo, A., Marshall, K., Cruz Portillo, V.M., Harvey, D.J., Ozorowski, G., Zitzmann, N., Wilson, I.A., et al. (2018). Integrity of glycosylation processing of a glycan-depleted trimeric HIV-1 immunogen targeting key B-cell lineages. J. Proteome Res. *17*, 987–999.

48. Brouwer, P.J.M., Antanasijevic, A., Berndsen, Z., Yasmeen, A., Fiala, B., Bijl, T.P.L., Bontjer, I., Bale, J.B., Sheffler, W., Allen, J.D., et al. (2019). Enhancing and shaping the immunogenicity of native-like HIV-1 envelope trimers with a two-component protein nanoparticle. Nat. Commun. *10*, 4272–4317.

49. Sanders, R.W., Derking, R., Cupo, A., Julien, J.-P., Yasmeen, A., de Val, N., Kim, H.J., Blattner, C., de la Peña, A.T., Korzun, J., et al. (2013). A

next-generation cleaved, soluble HIV-1 Env trimer, BG505 SOSIP.664 gp140, expresses multiple epitopes for broadly neutralizing but not non-neutralizing antibodies. PLoS Pathog. *9*, e1003618.

50. Karlsson, R., Mo, J.A., and Holmdahl, R. (1995). Binding of autoreactive mouse anti-type II collagen antibodies derived from the primary and the secondary immune response investigated with the biosensor technique. J. Immunol. Methods *188*, 63–71.

51. Yasmeen, A., Ringe, R., Derking, R., Cupo, A., Julien, J.-P., Burton, D.R., Ward, A.B., Wilson, I.A., Sanders, R.W., Moore, J.P., and Klasse, P.J. (2014). Differential binding of neutralizing and non-neutralizing antibodies to native-like soluble HIV-1 Env trimers, uncleaved Env proteins, and monomeric subunits. Retrovirology *11*, 41.

52. de Taeye, S.W., Ozorowski, G., Torrents de la Peña, A., Guttman, M., Julien, J.-P., van den Kerkhof, T.L.G.M., Burger, J.A., Pritchard, L.K., Pugach, P., Yasmeen, A., et al. (2015). Immunogenicity of stabilized HIV-1 envelope trimers with reduced exposure of non-neutralizing epitopes. Cell *163*, 1702–1715.

53. Cheng, H.D., Grimm, S.K., Gilman, M.S., Gwom, L.C., Sok, D., Sundling, C., Donofrio, G., Karlsson Hedestam, G.B., Bonsignori, M., Haynes, B.F., et al. (2018). Fine epitope signature of antibody neutralization breadth at the HIV-1 envelope CD4-binding site. JCI Insight *3*, e97018. https://doi.org/10.1172/jci.insight.97018.

54. LaBranche, C.C., Henderson, R., Hsu, A., Behrens, S., Chen, X., Zhou, T., Wiehe, K., Saunders, K.O., Alam, S.M., Bonsignori, M., et al. (2019). Neutralization-guided design of HIV-1 envelope trimers with high affinity for the unmutated common ancestor of CH235 lineage CD4bs broadly neutralizing antibodies. PLoS Pathog. *15*, e1008026.

55. Bale, J.B., Gonen, S., Liu, Y., Sheffler, W., Ellis, D., Thomas, C., Cascio, D., Yeates, T.O., Gonen, T., King, N.P., and Baker, D. (2016). Accurate design of megadalton-scale two-component icosahedral protein complexes. Science *353*, 389–394.

56. Abbott, R.K., Lee, J.H., Menis, S., Skog, P., Rossi, M., Ota, T., Kulp, D.W., Bhullar, D., Kalyuzhniy, O., Havenar-Daughton, C., et al. (2018). Precursor frequency and affinity determine B cell competitive fitness in germinal centers, tested with germline-targeting HIV vaccine immunogens. Immunity *48*, 133–146.e6.

57. Dosenovic, P., Kara, E.E., Pettersson, A.-K., McGuire, A.T., Gray, M., Hartweger, H., Thientosapol, E.S., Stamatatos, L., and Nussenzweig, M.C. (2018). Anti-HIV-1 B cell responses are dependent on B cell precursor frequency and antigen-binding affinity. Proc. Natl. Acad. Sci. USA *115*, 4743–4748.

58. Torrents de la Peña, A., Julien, J.-P., de Taeye, S.W., Garces, F., Guttman, M., Ozorowski, G., Pritchard, L.K., Behrens, A.-J., Go, E.P., Burger, J.A., et al. (2017). Improving the immunogenicity of native-like HIV-1 envelope trimers by hyperstabilization. Cell Rep. *20*, 1805–1817.

59. Julien, J.-P., Lee, J.H., Ozorowski, G., Hua, Y., Torrents de la Peña, A., de Taeye, S.W., Nieusma, T., Cupo, A., Yasmeen, A., Golabek, M., et al. (2015). Design and structure of two HIV-1 clade C SOSIP.664 trimers that increase the arsenal of native-like Env immunogens. Proc. Natl. Acad. Sci. USA *112*, 11947–11952.

60. Wiehe, K., Bradley, T., Meyerhoff, R.R., Hart, C., Williams, W.B., Easterhoff, D., Faison, W.J., Kepler, T.B., Saunders, K.O., Alam, S.M., et al. (2018). Functional relevance of improbable antibody mutations for HIV broadly neutralizing antibody development. Cell Host Microbe *23*, 759–765.e6.

61. Jardine, J.G., Sok, D., Julien, J.-P., Briney, B., Sarkar, A., Liang, C.-H., Scherer, E.A., Henry Dunand, C.J., Adachi, Y., Diwanji, D., et al. (2016). Minimally mutated HIV-1 broadly neutralizing antibodies to guide reductionist vaccine design. PLoS Pathog. *12*, e1005815.

62. Barnes, C.O., Schoofs, T., Gnanapragasam, P.N.P., Golijanin, J., Huey-Tubman, K.E., Gruell, H., Schommers, P., Suh-Toma, N., Lee, Y.E., Cetrulo Lorenzi, J.C., et al. (2022). A naturally arising broad and potent CD4-binding site antibody with low somatic mutation. Preprint at bioRxiv. https://doi.org/10.1101/2022.03.16.484662.

63. Evans, R., O'Neill, M., Pritzel, A., Antropova, N., Senior, A., Green, T., Žídek, A., Bates, R., Blackwell, S., Yim, J., et al. (2022). Protein complex prediction with alphafold-multimer. Preprint at bioRxiv. https://doi.org/10.1101/2021.10.04.463034.

64. Jumper, J., Evans, R., Pritzel, A., Green, T., Figurnov, M., Ronneberger, O., Tunyasuvunakool, K., Bates, R., Žídek, A., Potapenko, A., et al. (2021). Highly accurate protein structure prediction with AlphaFold. Nature *596*, 583–589.

65. Umotoy, J., Bagaya, B.S., Joyce, C., Schiffner, T., Menis, S., Saye-Francisco, K.L., Biddle, T., Mohan, S., Vollbrecht, T., Kalyuzhniy, O., et al. (2019). Rapid and focused maturation of a VRC01-class HIV broadly neutralizing antibody lineage involves both binding and accommodation of the N276-glycan. Immunity *51*, 141–154.e6.

66. Cirelli, K.M., Carnathan, D.G., Nogal, B., Martin, J.T., Rodriguez, O.L., Upadhyay, A.A., Enemuo, C.A., Gebru, E.H., Choe, Y., Viviano, F., et al. (2020). Slow delivery immunization enhances HIV neutralizing antibody and germinal center responses via modulation of immunodominance. Cell *180*, 206.

67. Kanekiyo, M., Joyce, M.G., Gillespie, R.A., Gallagher, J.R., Andrews, S.F., Yassine, H.M., Wheatley, A.K., Fisher, B.E., Ambrozak, D.R., Creanga, A., et al. (2019). Mosaic nanoparticle display of diverse influenza virus hemagglutinins elicits broad B cell responses. Nat. Immunol. *20*, 362–372.

68. Cohen, A.A., Gnanapragasam, P.N.P., Lee, Y.E., Hoffman, P.R., Ou, S., Kakutani, L.M., Keeffe, J.R., Wu, H.-J., Howarth, M., West, A.P., et al. (2021). Mosaic nanoparticles elicit cross-reactive immune responses to zoonotic coronaviruses in mice. Science *371*, 735–741.

69. Boyoglu-Barnum, S., Ellis, D., Gillespie, R.A., Hutchinson, G.B., Park, Y.-J., Moin, S.M., Acton, O.J., Ravichandran, R., Murphy, M., Pettie, D., et al. (2021). Quadrivalent influenza nanoparticle vaccines induce broad protection. Nature *592*, 623–628.

70. Verkoczy, L., Diaz, M., Holl, T.M., Ouyang, Y.-B., Bouton-Verville, H., Alam, S.M., Liao, H.-X., Kelsoe, G., and Haynes, B.F. (2010). Autoreactivity in an HIV-1 broadly reactive neutralizing antibody variable region heavy chain induces immunologic tolerance. Proc. Natl. Acad. Sci. USA *107*, 181–186.

71. Verkoczy, L., Chen, Y., Bouton-Verville, H., Zhang, J., Diaz, M., Hutchinson, J., Ouyang, Y.-B., Alam, S.M., Holl, T.M., Hwang, K.-K., et al. (2011). Rescue of HIV-1 broad neutralizing antibody-expressing B cells in 2F5 VH x VL knockin mice reveals multiple tolerance controls. J. Immunol. *187*, 3785–3797.

72. Chen, Y., Zhang, J., Hwang, K.-K., Bouton-Verville, H., Xia, S.-M., Newman, A., Ouyang, Y.-B., Haynes, B.F., and Verkoczy, L. (2013). Common tolerance mechanisms, but distinct cross-reactivities associated with gp41 and lipids, limit production of HIV-1 broad neutralizing antibodies 2F5 and 4E10. J. Immunol. *191*, 1260–1275.

73. Suloway, C., Pulokas, J., Fellmann, D., Cheng, A., Guerra, F., Quispe, J., Stagg, S., Potter, C.S., and Carragher, B. (2005). Automated molecular microscopy: the new Leginon system. J. Struct. Biol. *151*, 41–60.

74. Voss, N.R., Yoshioka, C.K., Radermacher, M., Potter, C.S., and Carragher, B. (2009). DoG Picker and TiltPicker: software tools to facilitate particle selection in single particle electron microscopy. J. Struct. Biol. *166*, 205–213.

75. Ogura, T., Iwasaki, K., and Sato, C. (2003). Topology representing network enables highly accurate classification of protein images taken by cryo electron-microscope without masking. J. Struct. Biol. *143*, 185–200.

76. Elsliger, M.A., Deacon, A.M., Godzik, A., Lesley, S.A., Wooley, J., Wüthrich, K., and Wilson, I.A. (2010). The JCSG high-throughput structural biology pipeline. Acta Crystallogr., Sect. F: Struct. Biol. Cryst. Commun. *66*, 1137–1142.

77. Otwinowski, Z., and Minor, W. (1997). Processing of X-ray diffraction data collected in oscillation mode. Methods Enzymol. *276*, 307–326.

78. Emsley, P., Lohkamp, B., Scott, W.G., and Cowtan, K. (2010). Features and development of Coot. Acta Crystallogr. D Biol. Crystallogr. *66*, 486–501.

79. Adams, P.D., Afonine, P.V., Bunkóczi, G., Chen, V.B., Davis, I.W., Echols, N., Headd, J.J., Hung, L.-W., Kapral, G.J., Grosse-Kunstleve, R.W., et al. (2010). PHENIX: a comprehensive Python-based system for macromolecular structure solution. Acta Crystallogr. D Biol. Crystallogr. *66*, 213–221.

80. Chen, V.B., Arendall, W.B., Headd, J.J., Keedy, D.A., Immormino, R.M., Kapral, G.J., Murray, L.W., Richardson, J.S., and Richardson, D.C. (2010). MolProbity: all-atom structure validation for macromolecular crystallography. Acta Crystallogr. D Biol. Crystallogr. *66*, 12–21.

81. Agirre, J. (2017). Strategies for carbohydrate model building, refinement and validation. Acta Crystallogr. D Struct. Biol. *73*, 171–186.

82. LaBranche, C.C., McGuire, A.T., Gray, M.D., Behrens, S., Kwong, P.D., Chen, X., Zhou, T., Sattentau, Q.J., Peacock, J., Eaton, A., et al. (2018). HIV-1 envelope glycan modifications that permit neutralization by germline-reverted VRC01-class broadly neutralizing antibodies. PLoS Pathog. *14*, e1007431.

83. Montefiori, D.C. (2009). Measuring HIV neutralization in a luciferase reporter gene assay. Methods Mol. Biol. *485*, 395–405.

84. Magoč, T., and Salzberg, S.L. (2011). FLASH: fast length adjustment of short reads to improve genome assemblies. Bioinformatics *27*, 2957–2963.

85. Kepler, T.B., Munshaw, S., Wiehe, K., Zhang, R., Yu, J.-S., Woods, C.W., Denny, T.N., Tomaras, G.D., Alam, S.M., Moody, M.A., et al. (2014). Reconstructing a B-cell clonal lineage. II. Mutation, selection, and affinity maturation. Front. Immunol. *5*, 170.

86. Yaari, G., Vander Heiden, J.A., Uduman, M., Gadala-Maria, D., Gupta, N., Stern, J.N.H., O'Connor, K.C., Hafler, D.A., Laserson, U., Vigneault, F., and Kleinstein, S.H. (2013). Models of somatic hypermutation targeting and substitution based on synonymous mutations from high-throughput immunoglobulin sequencing data. Front. Immunol. *4*, 358.

87. Tiller, T., Busse, C.E., and Wardemann, H. (2009). Cloning and expression of murine Ig genes from single B cells. J. Immunol. Methods *350*, 183–193.

88. Wang, Z., Raifu, M., Howard, M., Smith, L., Hansen, D., Goldsby, R., and Ratner, D. (2000). Universal PCR amplification of mouse immunoglobulin gene variable regions: the design of degenerate primers and an assessment of the effect of DNA polymerase 3' to 5' exonuclease activity. J. Immunol. Methods *233*, 167–177.

89. Brouwer, P.J.M., Caniels, T.G., van der Straten, K., Snitselaar, J.L., Aldon, Y., Bangaru, S., Torres, J.L., Okba, N.M.A., Claireaux, M., Kerster, G., et al. (2020). Potent neutralizing antibodies from COVID-19 patients define multiple targets of vulnerability. Science *369*, 643–650.

90. Bontjer, I., Land, A., Eggink, D., Verkade, E., Tuin, K., Baldwin, C., Pollakis, G., Paxton, W.A., Braakman, I., Berkhout, B., and Sanders, R.W. (2009). Optimization of human immunodeficiency virus type 1 envelope glycoproteins with V1/V2 deleted, using virus evolution. J. Virol. *83*, 368–383.

STAR★METHODS

KEY RESOURCES TABLE

REAGENT or RESOURCE	SOURCE	IDENTIFIER
Antibodies		
Anti-mouse B220-BV711	BDBiosciences	Cat# 563892
Anti-mouse-CD19- APC-R700	BDBiosciences	Cat# 565473
Goat F(ab')2 Anti-Mouse IgM	Southern Biotech	Cat# 1022-01
Anti-IgD BV510	BDBiosciences	Cat# 563110
Anti-IgM PE-Cy7	BDBiosciences	Cat# 552867
Anti-IgG1-FITC	BDBiosciences	Cat# 553443
Anti-IgG2a/2b-FITC	BDBiosciences	Cat# 553399
Anti-IgG3-FITC	BDBiosciences	Cat# 553403
Anti-Fas-BV605	BDBiosciences	Cat#740367
Anti-CD38-PEcy5	eBioscience	Cat# 15-0381-82
Goat Anti-Human IgG (HRP)	SeraCare	Cat# 5220-0277
Bacterial and virus strains		
Chemically competent DH5αEscherichia coli	Thermo Fisher Scientific	Cat#: 12879416
Biological samples		
Mouse sera, immunized	This study	N/A
Chemicals, peptides, and recombinant proteins		
FreeStyle 293F media	Thermo Fisher Scientific	Cat# 12338026
Opti-MEM Reduced Serum Medium	Thermo Fisher Scientific	Cat# 31985070
PEI MAX transfection reagent	Polysciences	Cat# 24765-1
Lipofectamine 2000	Life Technologies	Cat# 11668-019
Dulbecco's Modified Eagle Medium	Life Technologies	Cat# 41966052
Acetonitrile, 80%, 20% Water with 0.1% Formic Acid, Optima LC/MS	Fisher Scientific	Cat# 15431423
Water with 0.1% Formic Acid (v/v), Optima LC/MS Grade	Fisher Scientific	Cat# LS118-212
Acetonitrile	Fisher Scientific	Cat# 10489553
Trifluoroacetic acid	Fisher Scientific	Cat# 10155347
Procainamide hydrochloride	Abcam	Cat# ab120955
H2O18	Sigma-Aldrich	Cat# 329878
Dithiothreitol	Sigma-Aldrich	Cat# 43819
Iodacetamide	Sigma-Aldrich	Cat# I1149
Ammonium formate buffer	Waters	Cat# 186007081
Sodium cyanoborohydride	Sigma-Aldrich	Cat# 156159
DMSO	Sigma-Aldrich	Cat# D2438
Acetic acid	Fisher Scientific	Cat# 10384970
Peptide-N-glycosidase F	New England Biolabs	Cat# P0705S
Endoglycosidase H	New England Biolabs	Cat# P0702S
Mass spectrometry grade trypsin	Promega	Cat# V5280
Sequencing grade chymotrypsin	Promega	Cat# V1061
HBS-EP buffer	Cytiva	Cat# BR100188
Sensor Chips, CM5	Cytiva	Cat# 29149604
Aqueous buffer, 10mM Glycine-HCl pH2.0	Cytiva	Cat# BR100355
Penicillin	Sigma-Aldrich	P3032-10MU

(*Continued on next page*)

Continued		
REAGENT or RESOURCE	SOURCE	IDENTIFIER
Streptomycin	VWR	382-EU-100G
GIBCO DPBS	Life Technologies	Cat# 12559069
Glycyl Glycine 99+%	Fisher Scientific	Cat# 10540771
$MgSO_4$	VWR	Cat# 10034-99-8
TitriPlex III (EDTA)	VWR	Cat# 1.08418.1000
Triton X-100	Fisher Scientific	Cat# BP151500
Tris	Sigma-Aldrich	Cat# 10708976001
HCl	Biosolve	Cat# 084105
Glycine	VWR	Cat# 4500345965
Magnesium Chloride ($MgCl_2$)	VWR	Cat# 4500348228
Sodium Bicarbonate ($NaHCO_3$)	Life Technologies	Cat# 25080094
Sodium Chloride (NaCl)	Sigma-Aldrich	Cat# S7653-1KG
Sodium Acetate (NaAc)	VWR	Cat# 1.06268.1000
Citric Acid Monohydrate	Brunschwig	Cat# 36665.22
3,3′,5,5′-Tetramethylbenzidine (TMB)	Sigma-Aldrich	Cat# T-2885
H_2O_2	Brunschwig	Cat# CP26.1
Sulfuric Acid 95-97%	VWR	Cat# 1.00731.1010
Sodium Dodecyl Sulfate	Sigma	Cat# L5750-1kg
Glycerol	Thermo Fisher Scientific	Cat# 15514-011
Critical commercial assays		
Fluo-4 Direct™ Calcium Assay Kits	Thermo Fisher Scientific	Cat# F10471
MiSeq Reagent Kit v3 (600-cycle)	illumina	MS-102-3003
Deposited data		
BG505 GT1.2 in complex with PGT124 and gl-PGV20	This study	PDB 81EP
Next generation sequencing data	This study	NRA: 32312762-32312784
Experimental models: Cell lines		
HEK293F cells	ThermoFisher Scientific	Cat# R79007
Experimental models: Organisms/strains		
gl-CH31 knock-in mice	Duke University/DHVI & ABS	N/A
Oligonucleotides		
P5-H10-leader:TCGTCGGCAGCGTCAGATGTG TATAAGAGACAGCTGTCAGTAACTGTAGGTGTGT	This paper	N/A
P7-IgM:GTCTCGTGGGCTCGGAGATGTGTATAA GAGACAGCGAGGGGGAAGACATTTGGG	This paper	N/A
P7-IgG1: GTCTCGTGGGCTCGGAGATGTGTATA AGAGACAGAGACAGATGGGGGTGTCGTT	This paper	N/A
P7-IgG2b: GTCTCGTGGGCTCGGAGATGTGTAT AAGAGACAGAGACTGATGGGGGTGTTGTT	This paper	N/A
P7-IgG3: GTCTCGTGGGCTCGGAGATGTGTATA AGAGACAGACAGATGGGGCTGTTGTTGT	This paper	N/A
P7-IgA: GTCTCGTGGGCTCGGAGATGTGTATAA GAGACAGTGGTGGGATTTCTCGCAGAC	This paper	N/A
P5-ox-leader: TCGTCGGCAGCGTCAGATGTGTA TAAGAGACAGTGCTAATCAGTGCCTCAGTCATAA	This paper	N/A
P7-mouse Kappa: GTCTCGTGGGCTCGGAGATG TGTATAAGAGACAGTGGATGGTGGGAAGATGGAT	This paper	N/A
Software and algorithms		
FlowJo	FlowJo	https://www.bdbiosciences.com/en-us/products/software/flowjo-v10-software
Prism v8	Graphpad	https://www.graphpad.com

(*Continued on next page*)

Continued

REAGENT or RESOURCE	SOURCE	IDENTIFIER
Adobe Illustrator 2021	Adobe	https://www.adobe.com
R v.4.1.2	Comprehensive R archive network (CRAN)	https://cran.r-project.org/
RStudio 2022.02.3	RStudio	https://www.rstudio.com/
Other		
Superdex 200 Increase 10/300 SEC column	Cytiva	Cat# 28-9909-44
Vivaspin 20, 100.000 MWCO PES	Sartorius	Cat# VS2042
Steritop-GP Filter Unit 0.22μm	Millipore	Cat# SCGPT05RE
ELISA-plate, half-area, 96W	Greiner Bio One	Cat# 675061

RESOURCE AVAILABILITY

Lead contact

Further information and requests for resources and reagents should be directed to and will be fulfilled by the lead contacts, Dr. Laurent Verkoczy (laurent.verkoczy@absinstitute.org) and Dr. Rogier W. Sanders (r.w.sanders@amsterdamumc.nl).

Materials availability

The MAbs generated in this study will be available under an MTA with Amsterdam UMC.

Data and code availability

This paper does not report original code. NGS data have been deposited at NCBI sequence read archive (SRA) under accession numbers 32312762-32312784 and are publicly available as of the date of publication. The crystal structure reported in this manuscript has been deposited at the Protein Data Bank (PDB) under accession number 8E1P. Any additional information is available from the lead contacts upon request.

EXPERIMENTAL MODEL AND SUBJECT DETAILS

Mice

The heterozygous gl-CH31 KI (heterozygous knock-in; $V_HDJ_H^{+/-}$ x $V_\kappa J_\kappa^{+/-}$) vaccination model was generated on the C57BL/6 CD45.2$^+$ background, based on previously-described Ig locus-directed gene-targeting techniques.[44,70–72] Briefly, gl-CH31 ''HC only'' (i.e. $V_HDJ_H^{+/+}$) KI mice were first generated by knocking in the published V_HDJ_H rearrangement of the inferred gl-CH31, via replacement of the mouse J_H cluster with a gl-CH31 HC expression cassette (containing the promoter and split leader sequences of the J558 V_H family H10, positioned 5′ of the rearranged gl-CH31 V_HDJ_H mini-gene segment sequences), and intra-bred to achieve homozygosity. In parallel, recombinant ES cells bearing the murine LC kappa locus-targeted inferred gl-CH31 V_kJ_k rearrangement sequence were generated by replacing Jκ1 and Jκ2 with the gl-CH31 LC expression cassette (comprised of the VOx1 promoter and split leader located 5′ of the pre-recombined gl-CH31 VκJκ rearrangement), and intra-bred to derive homozygous gl-CH31 ''LC only'' i.e. $V_\kappa J_\kappa^{+/+}$ KI mice. Finally, homozygous gl-CH31 ''HC only'' and 'LC only'' KI strains were repeatedly inter-crossed until a fully homozygous gl-CH31 KI ($V_HDJ_H^{+/-}$ x $V_\kappa J_\kappa^{+/+}$) breeding colony was established, and in order to characterize pre-immune/naïve B-cell development and V(D)J repertoire diversity, relative to age and gender-matched wild type C57BL/6 mice (Figure S1).

To generate heterozygous gl-CH31 vaccine cohorts, fully homozygous gl-CH31 base breeders were crossed to wild type C57BL/6 mice. All gl-CH31 KI animals reported in this manuscript were 8-12 weeks of age (either homozygous ones used for naïve/pre-immune developmental and repertoire characterizations or heterozygous cohorts at the start of all vaccine studies), with equal numbers of males and females distributed across all experimental groups. All mice were housed in Duke University (Division of Laboratory and Animal Resources) facilities or the ABS vivarium, both under pathogen-free environments, 12h light/dark cycles at 20–25°C, in accordance with NIH guidelines. All animal procedures performed were approved by Duke University or ABS Institutional Animal Care and Use Committee (IACUC)-approved protocols.

Cell lines

HEK293F cells (ThermoFisher) were used to produce recombinant proteins and antibodies as described below, as per the manufacturer's instructions.

METHOD DETAILS

Immunizations

For all vaccinations, a minimum of 4 mice per vaccine group were immunized intraperitoneally with either 1X PBS (saline controls) or 25 μg of BG505 SOSIP trimer proteins, formulated in 60 μg of polyinosinic:polycytidylic acid (poly I:C) adjuvant, a toll-like receptor 3 agonist. Blood samples were collected either 7 days prior to immunization (pre-bleed), or 10 days after each immunization, and sera was isolated for downstream evaluation of CD4bs binding specificity by enzyme-linked immunosorbent assay (ELISA) and for virus neutralization potential. Serum samples were heat inactivated for potential complement activity at 56 °C for 30 min. All vaccinated mice were equally matched for gender across all immunization groups, and were 8-12 weeks of age at start of immunizations.

B cell phenotypic analysis by flow cytometry

Flow cytometric analysis of B cell development was performed as previously described. Briefly, single-cell suspensions from spleen and BM of 8-12 week-old naive gl-CH31 and WT B6 mice were generated by mechanical dissociation. After ACK lysis was used to remove red blood cells from the single cell suspensions, a total of 10^7 cells were first stained with LIVE/DEAD staining buffer (LifeTech), spun down, and then stained in FACS buffer (1x PBS pH 7.2, 3% FBS (Hyclone), 0.01% sodium azide) with pre-mixed combinations of fluorochrome-labeled MAbs to various cell surface markers, at titration-pre-determined optimal concentrations. Total B cells were gated as singlet, live $B220^+CD19^+$ lymphocytes. For further sub-fractionation (into various B cell developmental subsets), primary fluorophore-conjugated MAbs (all from BD Biosciences) used included the following: 0.5 μg/mL of anti-B220 BV650 (catalog #563893), anti-CD19 APCR700 (catalog #565473), anti-IgD BV510 (catalog #563110), anti-IgM PE-Cy7 (catalog #552867), anti-CD21 BV421 (catalog #562756), anti-CD23 FITC (catalog #553138), and anti-CD93 PECF594 (catalog #563805). Flow cytometric analysis of B cell reactivities for CH31 bNAb lineage-specific CD4bs specificity was performed using single-cell splenocyte suspensions from naive mice that were stained with fluorochrome-labeled wild-type and mutant (KO) eOD-GT8 60mer baits, as described above.

Env design and characterization

The BG505 SOSIP v4.1-GT1.2 trimer was created by taking the BG505 SOSIP v4.1 GT construct[36] and introducing a single point mutation (N279D) using the QuikChange site-directed mutagenesis kit (Agilent Technologies). Specific epitope knockouts, such as the GT1.2 CD4bs knockout (D368R/N279A) or N276-lacking Envs were created with the same method as described previously. All constructs had a hexahistidine (His) tag and were produced and purified using PGT145 affinity chromatography as previously described.[13,36,49]

Glycopeptide analysis by LC-MS

Glycopeptide analysis was performed by first denaturing aliquots of protein for 1 h in 50 mM Tris/HCl, pH 8.0 containing 6 M of urea and 5 mM of dithiothreitol (DTT). Next, the proteins were reduced and alkylated by adding 20 mM iodoacetamide (IAA) and incubated for 1 h in the dark, followed by incubation with DTT remove any residual IAA. The alkylated Env proteins were buffer-exchanged into 50 mM Tris/HCl, pH 8.0 using Vivaspin columns (3 kDa) and digested separately overnight using trypsin or chymotrypsin e (Mass Spectrometry Grade, Promega) at a ratio of 1:30 (w/w). The next day, the peptides were dried and extracted using C18 Zip-tip (MerckMillipore). The peptides were dried again, re-suspended in 0.1% formic acid and analyzed by nanoLC-ESI MS with an Easy-nLC 1200 (ThermoFisher) system coupled to a Fusion mass spectrometer (ThermoFisher) using higher energy collision-induced dissociation (HCD) fragmentation. Peptides were separated using an EasySpray PepMap RSLC C18 column (75 μm × 75 cm). A trapping column (PepMap 100 C18 3 μm (particle size), 75 μm × 2cm) was used in line with the LC prior to separation with the analytical column. The LC conditions were as follows: 275 minute linear gradient consisting of 0-32% acetonitrile in 0.1% formic acid over 240 minutes followed by 35 minutes of 80% acetonitrile in 0.1% formic acid. The flow rate was set to 200 nL/min. The spray voltage was set to 2.7 kV and the temperature of the heated capillary was set to 40 °C. The ion transfer tube temperature was set to 275 °C. The scan range was 400−1600 m/z. The HCD collision energy was set to 50%, appropriate for fragmentation of glycopeptide ions. Precursor and fragment detection were performed using an Orbitrap at a resolution MS1 = 100,000; MS2 = 30,000. The AGC target for MS1 = 4 x 10^5 and MS2 = 5e4 and injection time: MS1 = 50 ms; S2 = 54 ms.

Glycopeptide fragmentation data were extracted from the raw file Byos (Version 3.5; Protein Metrics Inc.) and evaluated manually for each glycopeptide. Peptides were scored as true positives when the correct b and y fragment ions were observed along with oxonium ions corresponding to the glycan identified. The protein metrics N309 mammalian glycan library was modified to include sulfated glycans. The relative amounts of each glycan at each site as well as unoccupied proportions were determined by comparing the extracted chromatographic areas for different glycotypes with an identical peptide sequence. All charge states for a single glycopeptide were summed. The precursor mass tolerance was set at 4 ppm and 10 ppm for fragments. A 1% false discovery rate (FDR) was applied. Glycans were categorized according to the composition detected. HexNAc(2), Hex(9−5) was classified as M9 to M5. HexNAc(3)Hex(5−6)Neu5Ac)(0-4) was classified as hybrid with HexNAc(3)Hex(5-6)Fuc(1)Neu5Ac(0-4) classified as fhybrid. Complex-type glycans were classified according to the number of processed antennae and fucosylation. Complex glycans are categorized as HexNAc(3)(X), HexNAc(3)(F)(X), HexNAc(4)(X), HexNAc(4)(F)(X), HexNAc(5)(X), HexNAc(5)(F)(X), HexNAc(6+)(X) and HexNAc(6+)(F)(X). Any glycan containing at least one sialic acid was counted as sialylated.

Ultra-high performance liquid chromatography (UPLC) of released glycans

Gel bands corresponding to BG505 GT1.2 were excised and N-linked glycans were released in-gel using PNGaseF (2 μg enzyme in 100 μL H_2O, New England Biolabs) at 37 °C overnight. The released glycans were fluorescently labelled with procainamide using 110 mg/mL procainamide and 60 mg/mL sodium cyanoborohydride in a buffer consisting of 70% DMSO, 30% acetic acid. For each sample, 100 μL of labelling mixture was added. Labelling was performed at 60 °C for 2 h. Excess label and PNGaseF were removed using Spe-ed Amide-2 cartridges (Applied Separations). The labelled glycans were analyzed on a Waters Acquity H-Class UPLC instrument with a Glycan BEH Amide column (2.1 mm × 150 mm, 1.7 μM, Waters). A gradient of two buffers; 50 mM ammonium formate (buffer A) and acetonitrile (buffer B) was used. Gradient conditions were as follows: initial conditions, 0.5 mL/min 22% buffer A, increasing buffer A concentration to 44.1% over 57.75 min. Following this the concentration of buffer A was increased to 100% at 59.25 min and held there until 66.75 min, while the flow rate was dropped to 0.25 mL/min. Excitation wavelength was 310 nm, emission wavelength 370 nm for detection of the procainamide label. Data were processed using Empower 3 software (Waters). The relative abundance of oligomannose-type glycans was measured by digestion with Endoglycosidase H (per sample in 20 μL volume) (New England Biolabs). Digested glycans were cleaned using a 96-well PVDF protein-binding membrane (Millipore).

Negative-stain electron microscopy

GT1.2 was diluted to 0.02 mg/mL in TBS. 3 μL was applied to carbon-coated 400-mesh copper grids, blotted with filter paper and stained with 2% (w/v) uranyl formate for 90 s. Micrographs were collected on a ThermoFisher Tecnai Spirit microscope operating at 120kV with a FEI Eagle CCD (4k) camera (2.06 Å/pixel; 52,000x magnification) using Leginon automated image collection software[73]. Particles were picked using DogPicker[74] and 6,831 particles were included in the final 2D classification using iterative multivariate statistical analysis (MSA)/multireference alignment (MRA, Figure 1B).[75]

Surface plasmon resonance (SPR)

SPR was used for analyzing the binding of MAbs (mature or germline versions) to regular (BG505 SOSIP v4.1) and germline-adapted (BG505 SOSIP v4.1 GT1.2) Env trimers. SPR sensorgrams were recorded on a Biacore 3000 instrument. All binding experiments were conducted at 25 °C with HBS-EP (0.01 M HEPES, 0.15 M NaCl, 3 mM EDTA, 0.005% v/v Surfactant P20, pH 7.4, Cytiva) as running buffer as described.[51] Briefly, anti-his antibody coupled to the surfaces of CM5-sensor chips was used for capturing His-tagged trimers. Trimers were captured in parallel flow cells to an immobilization level, R_L, of 210 RU (mean in response units, S.D. = 5.8 RU). IgG of mature and germline antibodies were diluted in two- or four-fold steps to get discernable stacking of curves starting at 1 μM and injecting lower concentrations until no detectable signal was obtained. In each cycle, IgG-trimer association was monitored for 300 s and then dissociation for 600 s at a flow rate of 50 mL/min. At the end of each cycle, the sensor surface with anti-his antibody was regenerated with 10 mM glycine for 60 s at a flow rate of 30 L/min. Two or three replicates (n) were performed for each MAb-trimer combination. The sensorgrams were analyzed with the BIAevaluation software (Cytiva). Reference-channel and zero-analyte control sensorgram curves were subtracted from the raw data to obtain response curves for specific binding. A bivalent model gave the best fit to the specific binding curves but with variable apparent contributions of the binding by the second Fab arm of the IgG molecule, i.e., the strengthening by two-point binding; the equilibrium constant, K_{D2} varied less than the kinetic constants, k_{on2} and k_{off2}, though, indicating an overall robustness of the modeling; the bivalent modeling gave eminently good fits, whereas a simple Langmuir model was unsatisfactory. The unit of the second component on-rate constant, k_{on2} (1/RUs) was converted to (1/Ms) by the formula, k_{on2} (1/Ms) = ko_{n2} (1/RUs) x M_A (g/mole) x 100,[50] where M_A is molar mass of analyte (1.5 x 10^5 g/mole for IgG) and 100 is a factor taking into account the optically relevant volume of the dextran matrix on the CM5-sensor chip and the signal per mass of protein, per volume.

Structural analysis

Fabs of PGT124 and gl-PGV20 were expressed in HEK293F cells and purified by CaptureSelect CH1-XL affinity chromatography followed by SEC on a Superdex 75 16/600 column. The BG505 SOSIP.v4.1-GT1.2 trimer was expressed in FreeStyle HEK293S cells and extracted from the supernatant using a GNL affinity column, followed by SEC on a Superdex 200 16/600 column. A complex was formed by combining BG505-PGT124-glPGV20 in a 1:2:2 molar ratio, followed by deglycosylation using endoH digestion at 37 °C for 1 h before SEC purification. The SEC-purified complex was screened at both 4 °C and 20 °C using a high-throughput CrystalMation robotic system (Rigaku)[76]. High-quality crystals of Fabs PGT124 and gl-PGV20 bound to the BG505 Env trimer were obtained in 0.2 M ammonium sulfate, 0.1 M Tris pH 8.5, 12% (w/v) PEG 8000 at 4 °C. Data were collected at the Advanced Photon Source (APS) on beamline 23-IDD. The bound Fabs PGT124 and gl-PGV20 to BG505 Env trimer crystals diffracted to 3.80 Å resolution. The data were indexed, integrated, and scaled using HKL2000 in $P2_1$ for the complex[77]. The BG505 SOSIP.v4.1-GT1.2 in complex with Fabs PGT124 and gl-PGV20 was determined by MR using PDB 5W6D for the Env trimer, the PDB 4R26 for PGT124 and PDB 4LSU for Fab gl-PGV20 as the search models. The crystal structure of the Env trimer complex was refined to R_{cryst}/R_{free} of 27.0/30.1 with 95.7% completeness (Table S2). Model building and refinement were carried out with Coot and Phenix, respectively[78,79]. Structure quality was determined by MolProbity[80]. The Kabat numbering scheme was used for Fabs and the BG505 trimer were numbered according to the HXB2 system. Structure validation was performed using the PDB Validation Server (https://validate.wwpdb.org), and Privateer.[81] Data collection and refinement statistics are outlined in Table S2.

Calcium flux analysis

Ex vivo evaluation of calcium signaling by primary splenic B cells was performed using a previously published flow cytometry-based approach.[44] Briefly, splenocytes from 8-12 week-old naive gl-CH31 dKI and WT B6 mice were harvested, and red blood cells (RBC) were lysed by incubation of pre-warmed 2 ml ACK Buffer (Life Technologies). After washing and re-suspension in HBSS, single cell suspensions were directly stained with HBSS + 3% FBS containing 0.5 μg/mL of anti-B220-BV711 (BD Cat# 563892) and APC-R700 anti-CD19 (Cat# 565473, clone 1D3) for 40 min. Cells were then washed and resuspended in HBSS, prior to loading with Fluo-4 via mixing with equal volumes of 2X Fluo-4 Direct™ calcium reagent loading solution (Fluo-4 Direct™ Calcium Assay Kits, ThermoFisher). After sequential 30 min incubations at 37 °C and 30 min at room temperature, cells were washed and incubated with LIVE/DEAD® NIR staining buffer for an additional 30 min. Finally, cells were washed and resuspended in calcium-containing HBSS and incubated at room temperature for 5 min, prior to their activation with either 50 μg/mL anti-IgM F(ab')2 (Southern Biotech) or varying doses of GT1.2-I53-50 NP. Fluo-4 MFI data for total ($B220^+CD19^+$) B cells was acquired on a BD LSR II flow cytometer and analyzed by FlowJo software.

Immunizations

For all vaccinations, a minimum of 4 mice per vaccine group were immunized intraperitoneally with either 1X PBS (saline controls) or 25 μg of BG505 SOSIP trimer proteins, formulated in 60 μg of polyinosinic:polycytidylic acid (poly I:C) adjuvant, a toll-like receptor 3 agonist. Blood samples were collected at either pre-bleed, or 10 days after each immunization to be tested for enzyme-linked immunosorbent assay (ELISA) binding and neutralization against viruses to isolate sera for downstream evaluation of ELISA binding and virus neutralization potential. Serum samples were heat inactivated for potential complement activity at 56 °C for 30 min. All vaccinated mice were equally matched for gender across all immunization groups, were 8-12 weeks of age at start of immunizations, and were housed in animal facilities accredited by the Association for Assessment and Accreditation of Laboratory Animal Care International (AAALAC), in accordance with NIH guidelines. All animal procedures performed were approved by Duke or CMII Institutional Animal Care and Use Committees (IACUC)-approved protocols.

Serum enzyme-linked immunosorbent assay (ELISA) assays

ELISAs performed to analyze the serum antibody response in immunized mice were adapted from.[36] In short, hexahistidine (his)-tagged Envs (2 μg/mL) were captured on Ni-NTA plates (Qiagen) and left overnight at RT. The next day, after the plates were washed with TBS, the serum was diluted in 2% skim milk/TBS supplemented with 20% sheep serum (Biotrading) and incubated for 2 h at 37°C (starting dilution 1:100). Following three washes with TBS, a 1:3000 dilution of HRP-labeled goat anti-human IgG (Jackson ImmunoResearch) in casein was added for 1 h at RT. After washing the plates five times with TBS/0.05% Tween-20, develop solution containing 100 mM sodium acetate, 100 mM citric acid, 0.01% hydrogen peroxide, and 1% 3,3′,5,5′-tetramethylbenzidine (TMB, Sigma-Aldrich) was added. After a set amount of time, the colorimetric reaction was terminated by adding 0.8M sulfuric acid. In Figure 2, values were normalized to the highest AUC obtained per time point in a particular assay.

Serum neutralization assays

Serum neutralization assays were performed as described elsewhere.[36,82,83] Neutralizing antibody activity was measured in 96-well culture plates by using Tat-regulated luciferase (Luc) reporter gene expression to quantify reductions in virus infection in TZM-bl cells. TZM-bl cells were obtained from the NIH AIDS Research and Reference Reagent Program, as contributed by John Kappes and Xiaoyun Wu. Assays were performed with Env-pseudotyped viruses as described previously.[83] Test samples were diluted over a range of 1:20 to 1:43740 in cell culture medium and pre-incubated with virus (~150000 relative light unit equivalents) for 1 h at 37°C before addition of cells, and tested in duplicate. For some viruses, samples were diluted 1:30 to 1:2343750 or 1:300 to 1:23437500 in order to achieve an end-point titer. Following a 48 h incubation, cells were lysed and Luc activity determined using a microtiter plate luminometer and BriteLite Plus Reagent (Perkin Elmer). Neutralization titers are the sample dilution (for serum) or concentration (for monoclonal antibodies) at which relative luminescence units (RLU) were reduced by 50% compared to RLU in virus control wells after subtraction of background RLU in cell control wells. Serum samples were heat-inactivated at 56°C for 30 min prior to assay. 426c.TM4 has four modifications compared to its parental 426c strain: S278R, G471S, N460D and N463D, thus lacking three glycans around the CD4bs (N276/N460/N463). 426c.SM has a single modification: N276D, thus lacking the N276 glycan.

Illumina next-generation sequencing (NGS)

Total RNAs of splenocytes collected from naive and immunized gl-CH31 dKI mice were extracted using the RNeasy Mini Kit (Qiagen) according to the manufacturer's protocol. Reverse transcription was performed using SuperScript IV with random primers, also according to the manufacturer instructions. After cDNA synthesis, knocked-in gl-CH31 V_HDJ_H rearrangements were amplified via 1st Round PCR using P5-H10-leader paired to P7 plus IgM, IgG1, IgG2b, IgG2c IgG3 or mouse IgA reverse primers. Likewise, knocked-in VJ rearrangements were amplified by PCR using P5-ox-leader primers paired to the P7-mouse kappa reverse primer. Phusion Hot Start Flex DNA polymerase (NEB, Cat# M0535) was used as polymerase enzyme. PCR products were gel-purified using QIAquick Gel extraction Kit (Qiagen) and Bar codes and Illumina sequencing tags were added to the purified amplicons by 2nd round PCR using Index Kit barcode-tagging primers (Illumina). The bar-coded PCR amplicons were individually purified with QIAquick Gel Extraction

Kit (Qiagen) and quantitated by qPCR (Kapa Sybr fast qPCR kit, Kapa Biosystems). The purified individually bar-coded amplicons were then pooled together at equal molar DNA. Pooled amplicons were further quantitated by qPCR, diluted at 4 nM, denatured and mixed with the denatured PhiX, and finally, loaded onto Illumina MiSeq kit V3 (2 × 300 base pairs; Illumina) cartridges for deep sequencing on an Illumina NovaSeq 6000 sequencer at the UCSD genomics core. After NGS, two mice in the "SOSIP long" group were determined to have genotypes inconsistent with the intended gl-CH31 dKI (heterozygous double knock-in; $V_HDJ_H^{+/-}$ x $V_\kappa J_\kappa^{+/-}$) genotype and were excluded from the study. Two additional mice were determined to have NGS libraries contaminated with non-gl-CH31 reads and were also excluded from the study.

Processing of NGS sequence data

Mouse HC and LC repertoire reads from NGS sequencing were processed and analyzed using an existing bioinformatics pipeline for analysis of bulk single short-read NGS sequencing of bNAb UCA knock-in mouse repertoires.[45] Briefly, immunized mice repertoire reads were assembled using FLASH,[84] quality filtered using the FASTX toolkit (http://hannonlab.cshl.edu/fastx_toolkit/), deduplicated, and annotated with immunogenetic information using Cloanalyst.[85] Reads that were identified as non-functional (e.g., out-of-frame, missing invariant Ig gene amino acids, presence of stop codons) were excluded from analysis. Frequencies of individual mutations in immunized mouse IgG repertoires were calculated after aligning NGS reads to the gl-CH31 KI sequence using in-house bioinformatics programs. Repertoire-level VRC01-class shared mutation plots were generated using methods described in.[37] Phylogenetic trees of gl-CH31 derived reads representing individual immunize mouse repertoire diversity were generated using 1000 randomly sampled functional heavy chain NGS repertoire reads and were constructed by neighbor-joining using Geneious Prime version 2022.2.1 (https://www.geneious.com) and visualized using the ggtree package in R. Reconstruction of potential maturation pathways of the isolated MAb ("pseudo-lineages") was performed using a method that we developed for searching NGS reads from the repertoire of the mouse that elicited the MAb. Based on maximum parsimony, the goal of the method is to find the shortest path distance from the MAb sequence to the gl-CH31 KI sequence using NGS reads as estimates of ancestral intermediate sequences along a lineage between the gl-CH31 and the MAb. Using the MAb sequence as the query sequence, we performed a nearest-neighbor search of repertoire reads (for MAb A27, n>250,000 reads) to identify the closest NGS read to the MAb that had fewer nucleotide mutations than the MAb. Then using the identified nearest-neighbor read, we repeated this procedure to form a chain of sequences ascending from the MAb to the gl-CH31 KI sequence. At each step, if ties are encountered, sub-lineages are searched recursively to ensure the shortest path between the MAb and gl-CH31 is identified. Due to the limits of maximum parsimony-based methods, the pseudo-lineages generated by this method are only intended to be used as a rough estimate of the lineage evolution of the elicited MAb. Pseudo-lineage trees were visualized with FigTree 1.4.4 (https://github.com/rambaut/figtree). Probabilities of antibody amino acid substitutions were estimated using the ARMADiLLO program.[60] Briefly, given a UCA sequence and the number of mutations observed in the antibody sequence of interest, ARMADiLLO simulates SHM based on a model of AID targeting and base substitution[86] and uses these simulations to estimate the probability of observing an amino acid at a specific position in the absence of antigenic selection. Improbable mutations were defined as amino acid substitutions estimated at <2% probability unless otherwise noted. Repertoire-level ARMADiLLO runs were performed using a customized version of ARMADiLLO using precomputed SHM simulations of gl-CH31 to allow for tractability of analyzing NGS size datasets (>100,000 sequences).

Sorting of single CD4bs-specific memory B cells

10-12 days after final boosts, spleens were removed from gl-CH31 dKI mice, smashed by sterile syringes, and passed through cell strainers. Cells were washed with DPBS and centrifuged at 350g for 5 min. After RBC lysis, cells were incubated with Live/Dead NIR for 30 min. Cells were further washed and stained with 3% FBS-supplemented HBSS buffer containing eOD-GT8-BV421 and knockout eOD-GT8-AF647 labeled tetramers along with premixed combinations of fluorochrome-labeled MAbs. Primary labeled MAb (all from BD Biosciences except those noted) used were: 0.5 mg/ml of BV650 anti-B220 (Cat# 563893, clone RA3-6B2), APC-R700 anti-CD19 (Cat# 565473, clone 1D3), BV510 anti-IgD (Cat#563110, clone 11-26c.2a), FITC anti-IgG1 (Cat# 553443, clone A85-1), anti-IgG2a/2b (Cat# 553399, clone R2-40), anti-IgG3 (Cat# 553403, clone R40-82), PE-Cy7 anti-IgM (Cat#552867, clone R6-60.2), BV605 anti-Fas (CD95, Cat#740367, clone Jo2), PECF594 anti-CD93 (Cat#563805, clone AA4.1) and 0.2 mg/ml of PE anti-mouse T- and B cell Activation Antigen (Cat#561530, clone GL7), PEcy5 anti-CD38 (eBioscience, Cat# 15-0381-82, clone 90) and BV711 anti-CD138 (Cat#563193, clone 281-2). IgG^+ memory B cells were visualized by first gating on singlets, followed by lymphocyte gating. Dead cells were then excluded using LIVE/DEAD NIR stain discrimination, subsequent gating for $B220^+CD19^+$ (total B cells), followed by further sub-gating for $CD38^+IgG^+IgM^-$ (class-switched/activated memory B cells). Finally, $eOD\text{-}GT8^+KO^-IgG^+$ memory single B cells were sorted into 96-well PCR plates containing 17 μL of SuperScript® IV reverse transcriptase buffer and RNaseOUT (LifeTech) using a FACSAria II (BD Biosciences, San Jose, CA). Sorted plates were frozen in a dry ice ethanol bath and stored at -80°C until further processing.

Isolation of mouse antibody genes by single cell PCR

Heavy chain (HC) V_HDJ_H and light chain (LC) $V_\kappa J_\kappa$ rearrangement pairs from single sorted memory B cells were recovered via nested PCR based on previous methods.[44,87,88] Briefly, cDNAs from single wells in sorted 96-well plates were generated by RT synthesis using SuperScript® IV Reverse Transcriptase (ThermoFisher Cat #18090010) and random primers (Random Hexamer, ThermoFisher, Cat# 48190-011) according to manufacturer's instructions, followed by two rounds of PCR amplification using Ig

reverse and forward primer sets. For isolation of knock-in HC rearrangements, forward primers (outer and inner) were used that were specific for the common J558 H10 leader in the knock-in gl-CH31 HC expression cassette. Parallel PCR amplifications using degenerate VH family-specific primer mixtures[88] were also used to detect mouse endogenous HC rearrangements. For all HC rearrangements isolated (KI or WT), reverse primer mixtures of γC1, γC2b, γC2c, γC3, and total γC-specific primers were used to isolate class-switched B cells. Likewise, for isolation of LC rearrangements, forward primers specific to either the common Ox1 leader in the knock-in gl-CH31 LC expression cassette were used, whereas forward degenerate V or V leader, in combination with reverse κC- or λC-specific primers were used to detect endogenous LC kappa or lambda rearrangements, respectively. Cloned PCR products were then gel purified and directly sequenced in both orientations (GeneWiz) and $V_{H/L}$, D_H, and $J_{H/L}$ segment usage was determined by querying amplified sequences to both the original gl-CH31 rearrangements and relative to C57BL/6 Ig germline sequences in the NIH IgBlast tool. gl-CH31 knock-in $V_H DJ_H$ and $V_\kappa J_\kappa$ knock-in rearrangements sequenced in both directions were analyzed for SHM using Lasergene software.

Monoclonal antibody (MAb) production and characterization

MAbs were transfected into HEK293F cells (ThermoFisher) and purified by protein A (ThermoFisher) affinity chromatography as previously described.[89] ELISA characterization was performed by coating 2 μg/mL of Env onto 96-well Ni-NTA plates (Qiagen) and incubating overnight at RT as described above, with the exception that three-fold serial MAb dilutions were made in casein (starting at 1 μg/mL, 10 μg/mL or 100 μg/mL). Neutralization assays were performed as previously described.[49,90] Briefly, neutralization experiments were set up to measure the ability of MAbs to reduce luciferase gene expression in adherent TZM-bl cells. These cells have been modified to include firefly luciferase genes, which are under control of an HIV-1 promotor. Virus was incubated with serially diluted MAbs for 1 h at 37 °C. 400 nM saquinavir and 40 μg/mL DEAE were added to the cells before addition of the MAb/virus mixture. Cells were grown for three days at 37 °C, after which cells were lysed and luciferase activity was measured using Bright-Glo (Promega) substrate on a GloMax Discover machine.

QUANTIFICATION AND STATISTICAL ANALYSIS

Statistical analyses were performed using GraphPad Prism 8 or Rstudio. Bars plotted represent the mean and standard deviation when error bars are present. In some cases where parts of whole or absolute numbers are plotted in a bar chart (e.g., Figures 3E and 4B), no error bars are present. Graphs plotted on a log scale represent the geometric mean and geometric standard deviation. Statistical details of experiments are described in the Method Details or Figure Legends – in Figure 3, unpaired, non-parametric Mann-Whitney tests were used to determine statistical significance. Calculated p values of less than 0.05 were treated as significant differences. For ELISA experiments, area under the curve was determined using the Prism 8 function “Area under the curve”, midpoint titers (EC_{50}s) were calculated using the function “sigmoidal dose-response (variable slope)” and neutralization titers (IC_{50}s) were calculated using the function “log(inhibitor) vs. response (variable slope)”. The number of mice and sequences for each experiment are indicated in the Figures, Figure legends and/or method details.

Cell Reports Medicine, Volume *4*

Supplemental information

Germline-targeting HIV-1 Env vaccination induces

VRC01-class antibodies with rare insertions

Tom G. Caniels, Max Medina-Ramírez, Jinsong Zhang, Anita Sarkar, Sonu Kumar, Alex LaBranche, Ronald Derking, Joel D. Allen, Jonne L. Snitselaar, Joan Capella-Pujol, Iván del Moral Sánchez, Anila Yasmeen, Marilyn Diaz, Yoann Aldon, Tom P.L. Bijl, Sravani Venkatayogi, Joshua S. Martin Beem, Amanda Newman, Chuancang Jiang, Wen-Hsin Lee, Maarten Pater, Judith A. Burger, Mariëlle J. van Breemen, Steven W. de Taeye, Kimmo Rantalainen, Celia LaBranche, Kevin O. Saunders, David Montefiori, Gabriel Ozorowski, Andrew B. Ward, Max Crispin, John P. Moore, Per Johan Klasse, Barton F. Haynes, Ian A. Wilson, Kevin Wiehe, Laurent Verkoczy, and Rogier W. Sanders

Figure S1

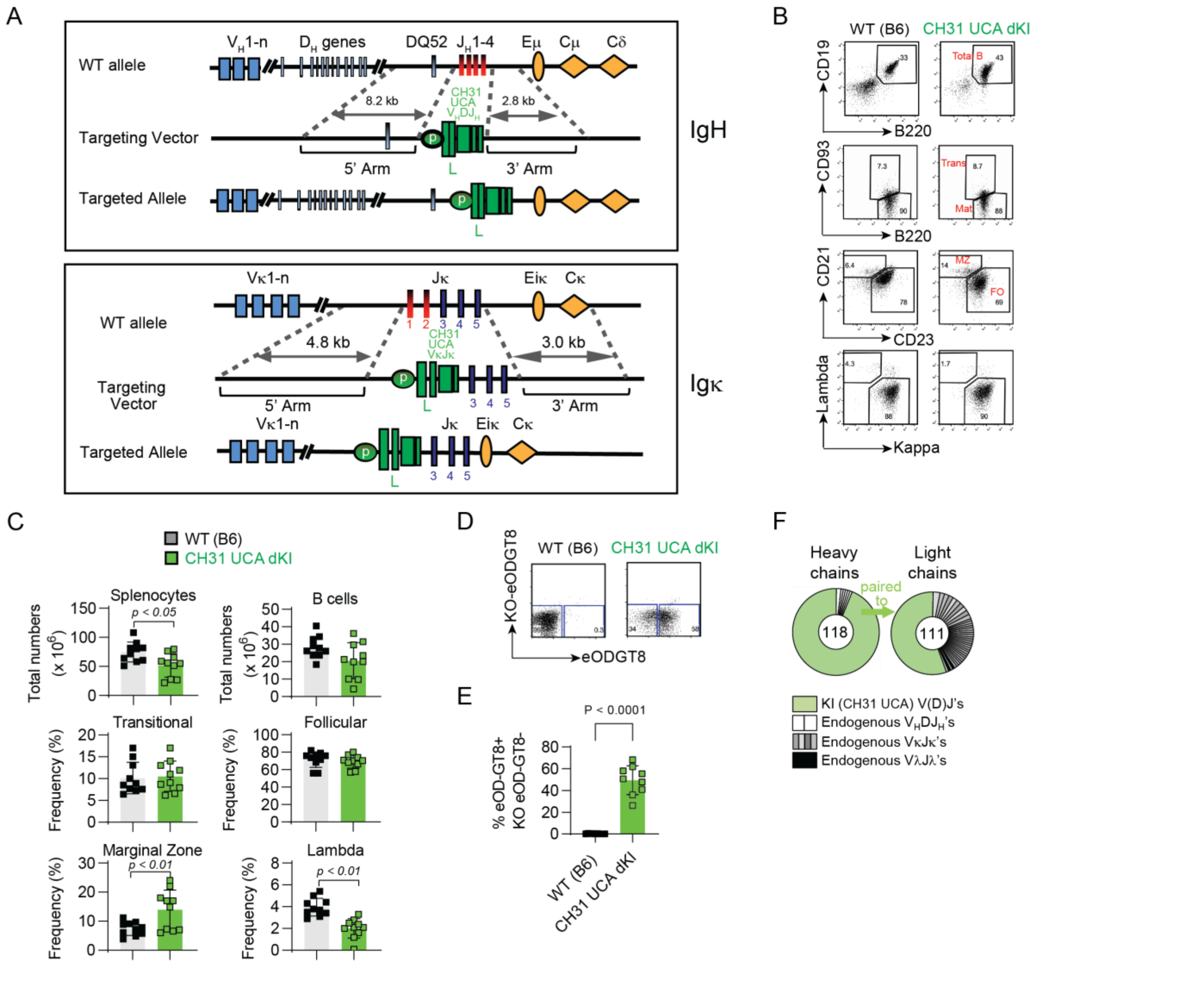

Supplemental Figure 1. Generation and characterization of gl-CH31 KI (V_HDJ_H x VκJκ) mice. Related to Figures 2-6. (A) Schematic depiction of the Ig-directed targeting strategies used to insert the CH31 UCA V_HDJ_H and VκJκ rearrangements into the mouse J_H and Jκ clusters, respectively. In each, top rows represent the wild type Ig loci, middle rows depict the targeting vector DNA donor constructs (illustrating the CH31 expression cassettes between the 5' and 3' homology arms), and bottom rows represent correctly targeted Ig loci, after homologous recombination. The CH31 heavy chain (HC) expression cassette is comprised of the murine H10 V_HJ558 family promoter (p) and split leader (L) in front of the rearranged human CH31 UCA V_HDJ_H coding segment (CH31 UCA V_HDJ_H), while the CH31 light chain (LC) expression cassette contains a VkOx1 promoter (p) and split leader (L) in front of the rearranged CH31 UCA VκJκ coding segment (CH31 UCA VκJκ). (B-C) Developmental B-cell subset analysis in 8-12 wk naïve, fully homozygous ($V_HDJ_H^{+/+}$ x $V\kappa J\kappa^{+/+}$) gl-CH31 KI mice. (B) FACS histograms representative of splenic B-cell development, indicating percentages of total live splenocytes that are $B220^+CD19^+$ (total B-cells; top row), or Transitional (Trans; $B220^{int}$ $CD93^+$) and Mature (Mat; $B220^{hi}$ CD93-) B-cell subsets (2nd row). Total B-cells were also further fractionated into Marginal Zone (MZ; $CD21^{hi}$ $CD23^{lo}$) or Follicular (FO; $CD21^{lo}$ $CD23^{hi}$) B-cell subsets (3rd row), and into kappa+ or lambda+ LC B-cells (bottom row). (C) Graphical representations depicting total numbers of splenocytes (upper left), and, based on gating scheme described in Fig 1B, total numbers of B cells (upper right) or frequencies of Transitional (left middle), Follicular (middle right), Marginal Zone (bottom left), and λLC-expressing B cells (bottom right) in the spleens of wild-type C57BL/6 (WT B6) and CH31 V_HDJ_H KI mice. Individual WT B6 or gl-CH31 KI mice are indicated by closed or open squares, respectively. Standard deviations are denoted by black error bars, and significance values were determined by a two-

tailed Student's t-test, with only significant differences denoted. (D-F) Ig repertoire analysis in heterozygous ($V_HDJ_H^{+/-}$ x $V\kappa J\kappa^{+/-}$) gl-CH31 KI mice. (D) Flow histogram reactivity profile of heterozygous gl-CH31 KI peripheral compartment for CD4bs binding that is prototypical of the CD4 mimic bNAb class. Total peripheral B-cells (singlet, live, $CD19^+$ $B220^+$ splenocytes) from 8-12 week naïve, heterozygous gl-CH31 KI mice were further gated for CD4bs VRC01-class specificity by staining with a WT and mutant (KO) pair of fluorescently labeled eOD-GT8 multimer baits, with 'on-target' binders defined by staining for WT, but not KO eOD-GT8. (E) Graphical representation of on-target CD4bs+ binders in spleens of naïve heterozygous gl-CH31 KI mice (as determined above for Fig. S1D), in relation to baseline levels of binding in age-matched, naïve WT B6 mice. Circles represent individual mice, with group means denoted by black bars. The p value was calculated using a two-tailed student's t-test. (F) HC/LC usage in the peripheral B-cell repertoire of naïve heterozygous gl-CH31 KI mice (n=3). Shown are pie charts of HC/LC rearrangement pairs from individually-sorted B-cells from the total (unselected) splenic B-cell repertoire, with a breakdown of either the knocked-in human Vκ1-33-bearing LC rearrangement (green slices; right pie) or other endogenous (VκJκ or VλJλ rearrangements used; denoted by gray or black slices, respectively), amongst LCs from single cells, paired to the knocked-in human CH31 UCA V_H1-2-bearing HC rearrangement (green slices; left pie). Middle circles represent number of single-cell rearrangements sequenced.

Figure S2

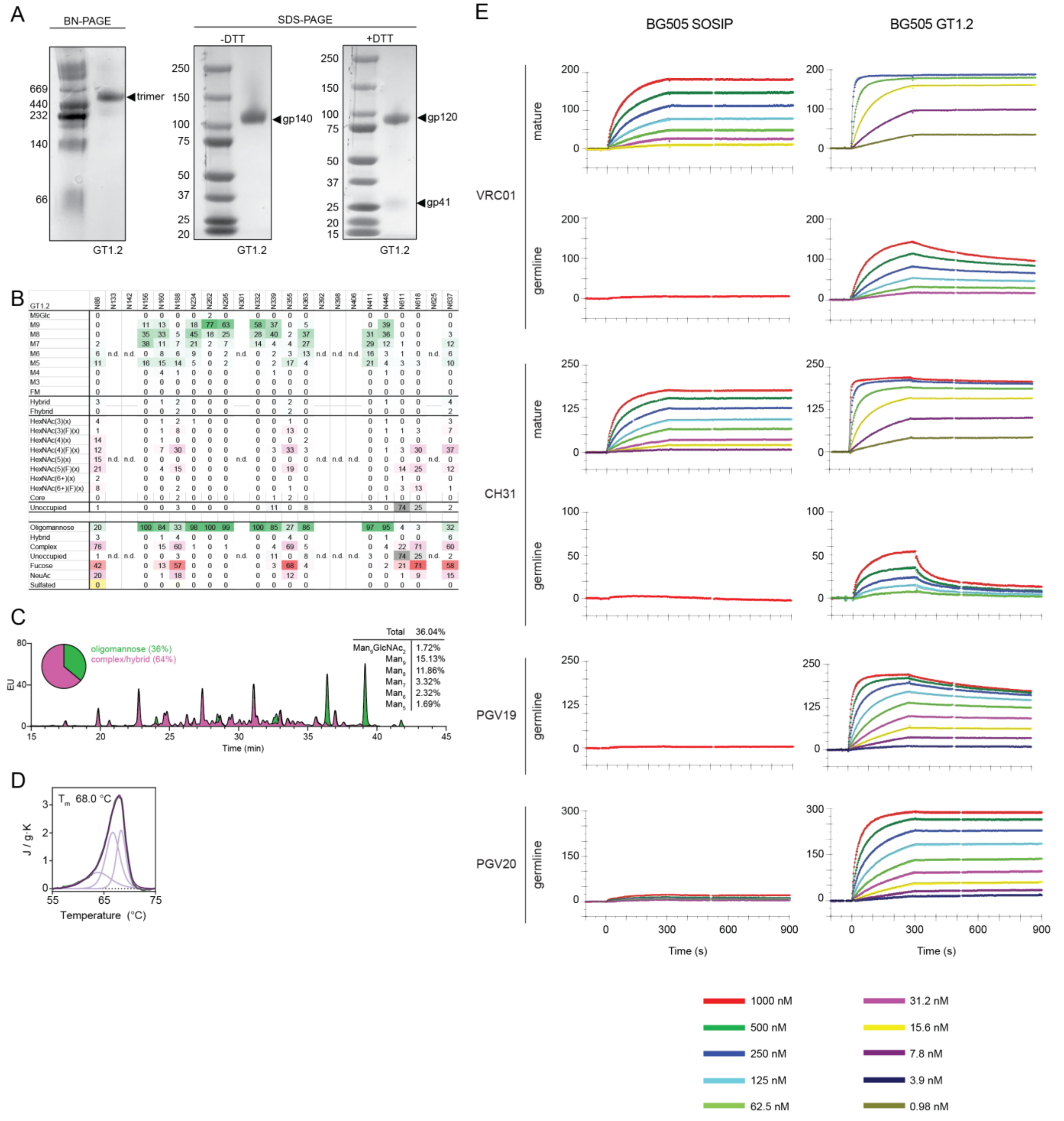

Supplemental Figure 2. Biochemical and antigenic characterization of BG505 GT1.2. Related to Figure 1. (A) Blue-native polyacrylamide gel electrophoresis (BN-PAGE) and sodium dodecyl sulfate PAGE (SDS-PAGE) in non-reducing (-DTT) and reducing (+DTT) conditions. (B) The data sets show the glycoforms found at each PNGS. Data for oligomannose/hybrid-type glycans are shaded in green, fully processed complex type glycans are shaded in magenta, while the absence of a glycan from some PNGS is shaded in grey. Oligomannose-type glycans are categorized according to the number of mannose residues present, hybrids by the presence/absence of fucose and complex-type glycans by the number of processed antenna and the presence/absence of fucose. (C) HILIC-UPLC analysis of BG505 GT1.2. Peaks colored green represent endoH-cleavable glycans (oligomannose/hybrid). The spectrum shows the overall oligomannose (green) and complex/hybrid (magenta) content. (D) Differential scanning calorimetry (DSC) analysis of BG505 GT1.2. The Tm value is indicated. (E) The sensorgrams show the specific binding signal in response units (RU) on the y-axes as a function of time during association and dissociation on the x-axes

(s). The color-coded legend gives the NAb concentrations for the empirical binding curves; the curves fitted with a model for bivalent binding are in black.

Figure S3

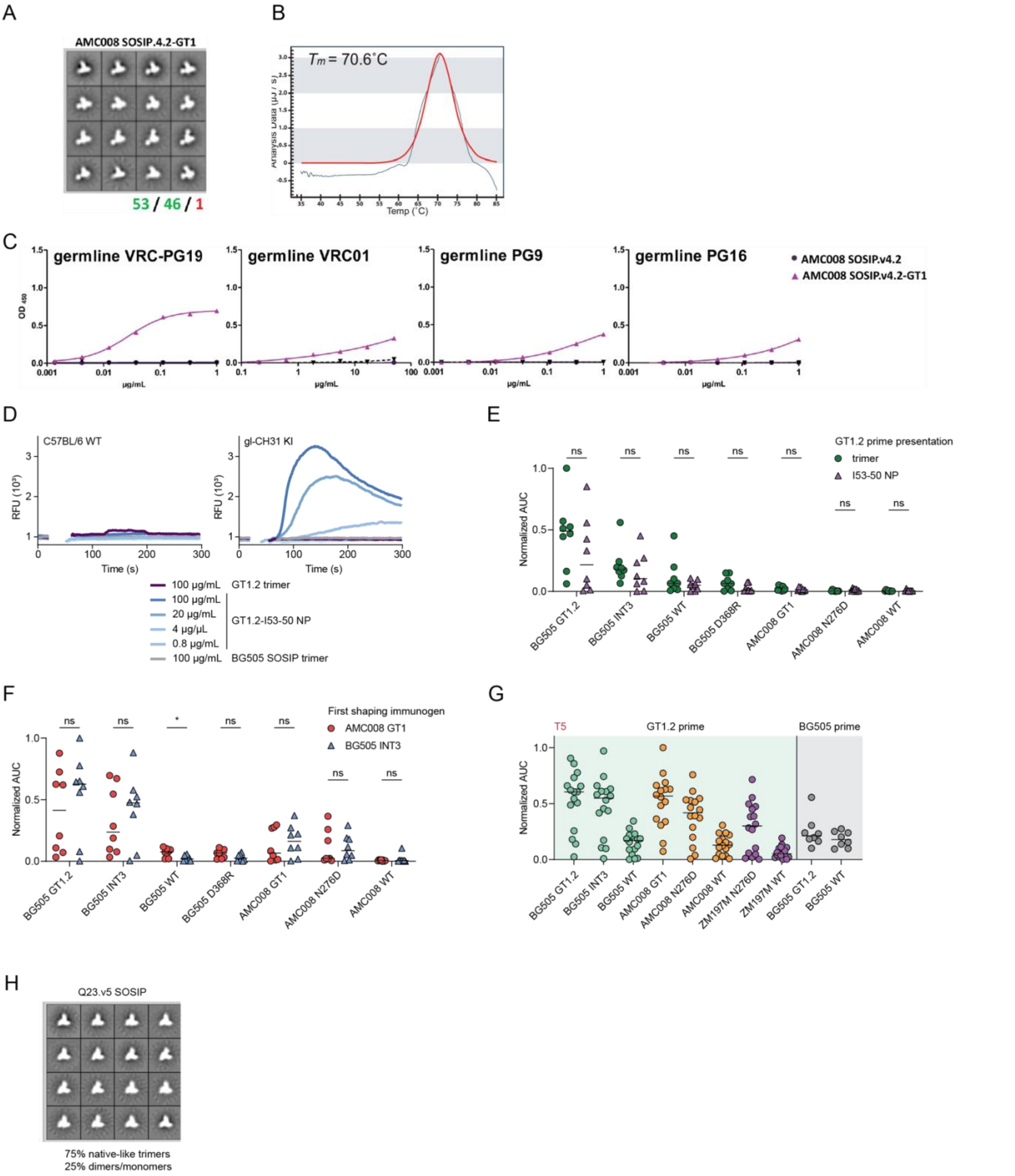

Supplemental Figure 3. Stability and antigenicity of AMC008 GT1, *ex vivo* gl-CH31 B cell activation and serum antibody binding of immunized animals. Related to Figures 1 and 2 (A) The AMC008 SOSIP.v4.2 clade B trimer (de Taeye et al., 2015) is based on an early sequence of an individual participating in the ACS that eventually developed bNAbs. We made the corresponding AMC008 SOSIP.v4.2-GT1 trimers that bear the same mutations as found in BG505 GT1. The resulting trimers are native-like and largely present as closed trimers. The numbers represent the percentage of native-like closed/native-like open/non-native-like trimers. (B) Differential scanning calorimetry (DSC) analysis of AMC008 GT1. The Tm value is indicated (Tm, 70.6°C). (C) ELISA graphs showing AMC008 SOSIP.v4.2 trimers were unable to bind any of the gl-bNAbs tested at the

maximum concentration. However, its corresponding GT1 version bound efficiently to gl-PGV19, gl-PG9 and gl-PG16 and also reacted with gl-VRC01, albeit more weakly. (D) Calcium flux assay as a measure for B cell activation from WT C57BL/6 B cells (left) and gl-CH31 KI B cells with the proteins indicated below. (E) Normalized area-under-the-curve (AUC) values of serum antibody binding to the indicated Env as measured by enzyme-linked immunosorbent assay (ELISA) at time point T2 (Fig. 2D). Each dot represents an individual mouse and the colors represent the priming immunogen received (green, GT1.2 trimer; purple, GT.2-I53-50 nanoparticles). (F) Normalized AUC values of serum antibody binding to the indicated Env at time point T3. Each dot represents an individual mouse and the colors represent the first shaping immunogen received (red, AMC008 GT1; blue, BG505 INT3). (G) Normalized AUC values of serum antibody binding to the indicated Env at time point T5, depicted as in Fig. 2E-G. Each dot represents an individual mouse and the background color represents the priming group (GT1.2 trimer/NP vs. BG505). The shading of the dots indicates the clade of the Env trimer (green, A; orange, B; purple, C).

Figure S4

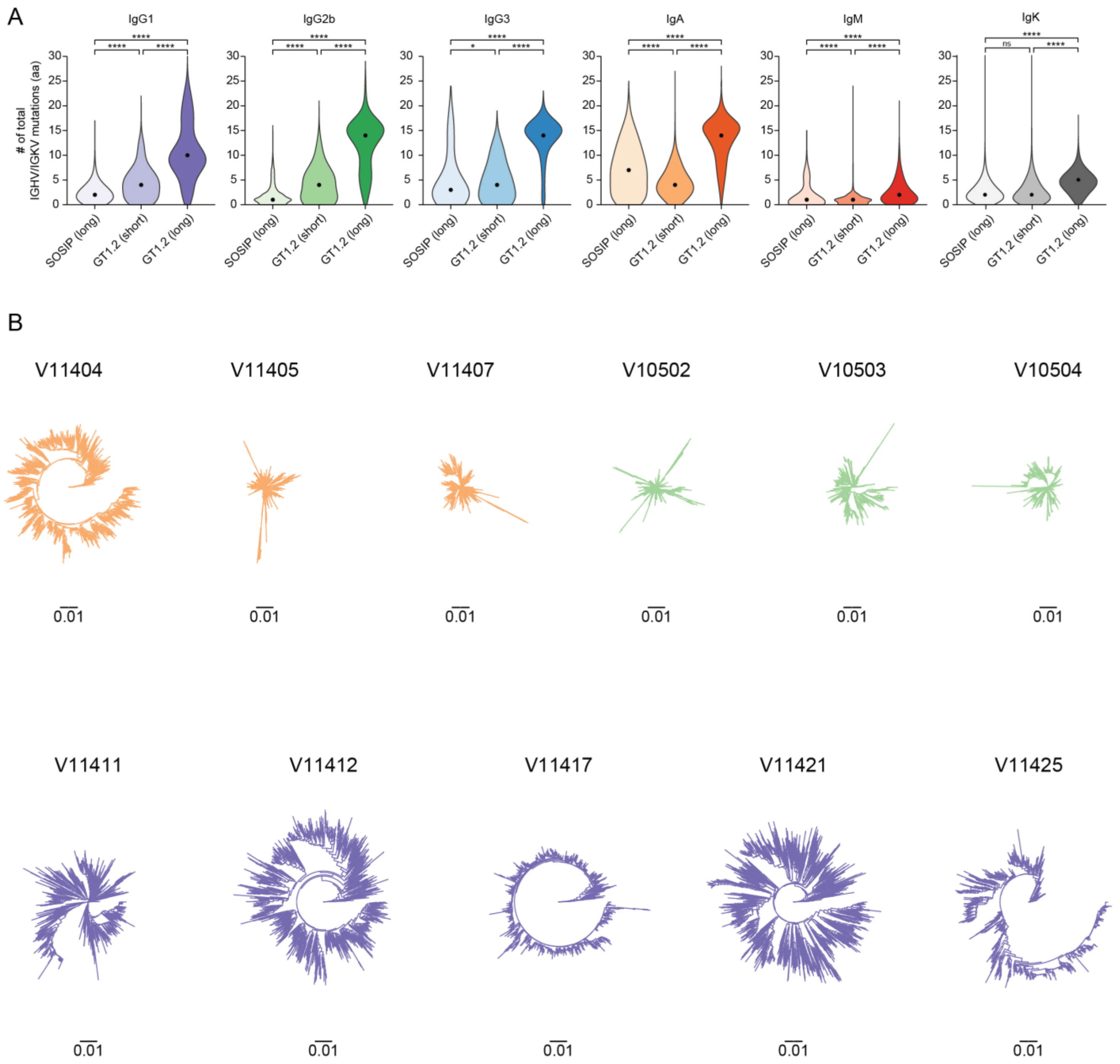

Supplemental Figure 4. Nonsynonymous mutations induced by immunization regimens and phylogenetic reconstructions. Related to Figure 3. (A) Violin plot showing the number of total amino acid substitutions in the IGHV region for each group (x-axes) for each Ig subtype. *, p < 0.05; ****, p < 0.0001. (B) Clonal diversification of immunized mice (each tree represents one mouse) as shown by circular trees of randomly sampled reads from the repertoires of each immunized mouse in the SOSIP-primed (orange), "GT1.2 (short)" (green) and "GT1.2 (long)" (purple) groups. Scale is set to a phylogenetic distance of 0.01 nucleotide substitutions per site for all trees and the trees are rooted on the gl-CH31 sequence.

Figure S5

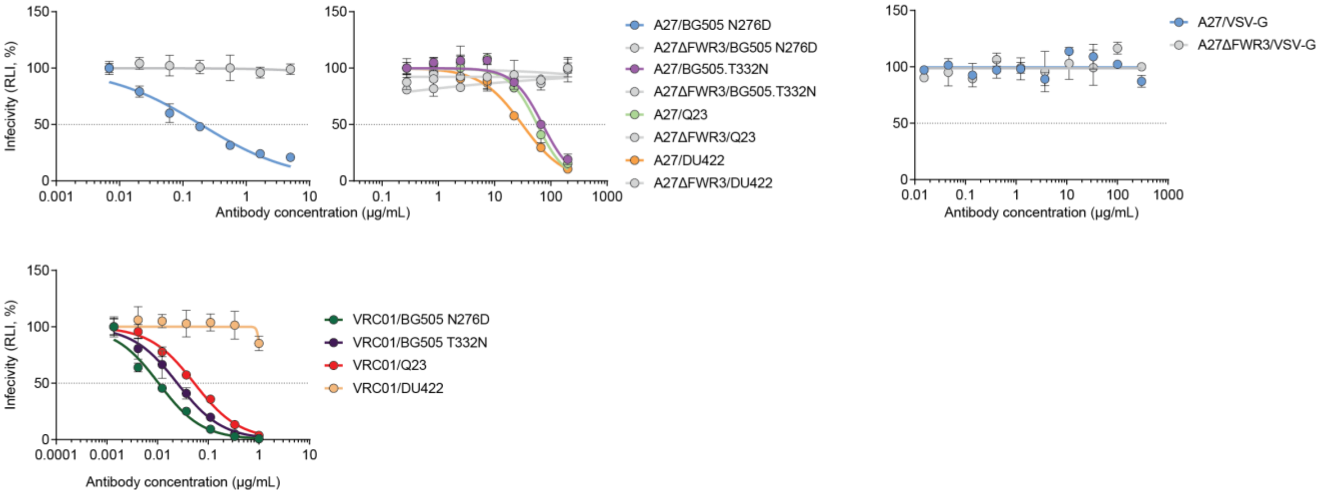

Supplemental Figure 5. A27 and A27ΔFWR3 neutralization. Related to Figures 4 and 5. The dotted line represents the midpoint neutralization titer (IC_{50}). Each dot represents the mean of two independent experiments (n=2) performed in triplicate.

NAb (IgG)		Env trimer	k_{on1} (1/Ms)	k_{off1} (1/s)	K_{D1} (nM)	k_{on2} (1/Ms)	k_{off2} (1/s)	K_{D2} (nM)	S_m
**VRC01**	*mature*	BG505 SOSIP.v4.1 (n=3)	$9.6 \cdot 10^3$ ± 3.5 10^2	$< 10^{-5}$	< 10	$6.8 \cdot 10^4$ ± $9.8 \cdot 10^3$	$9.2 \cdot 10^{-2}$ ± $1.5 \cdot 10^{-2}$	$1.3 \cdot 10^3$ ± 53	2.2 ± $9.3 \cdot 10^{-2}$
		BG505 SOSIP.v4.1-GT1.2 (n=2)	$2.6 \cdot 10^5$ ± $5.0 \cdot 10^3$	$< 10^{-5}$	< 0.10	$1.3 \cdot 10^3$ ± 15	$2.1 \cdot 10^{-3}$ ± 0	$1.7 \cdot 10^3$ ± 20	2.1 ± $4.9 \cdot 10^{-3}$
	germline	BG505 SOSIP.v4.1 (n=2)	Minimal binding; <10 RU						
		BG505 SOSIP.v4.1-GT1.2 (n=2)	$3.9 \cdot 10^3$ ± 60	$2.6 \cdot 10^{-3}$ ± $1.1 \cdot 10^{-4}$	$6.7 \cdot 10^2$ ± 37	$3.8 \cdot 10^7$ ± $1.1 \cdot 10^7$	20 ± 5.8	$5.4 \cdot 10^2$ ± 7.3	2.7 ± $8.5 \cdot 10^{-3}$
**CH31**	*mature*	BG505 SOSIP.v4.1 (n=3)	$1.4 \cdot 10^4$ ± $3.1 \cdot 10^2$	$< 10^{-5}$	< 1.0	$8.6 \cdot 10^4$ ± $3.6 \cdot 10^4$	0.14 ± $6.2 \cdot 10^{-2}$	$1.7 \cdot 10^3$ ± 54	2.2 ± $9.5 \cdot 10^{-2}$
		BG505 SOSIP.v4.1-GT1.2 (n=3)	$3.7 \cdot 10^5$ ± $2.2 \cdot 10^4$	$5.0 \cdot 10^{-5}$ ± $8.2 \cdot 10^{-6}$	0.14 ± $3.2 \cdot 10^{-2}$	$1.7 \cdot 10^4$ ± $2.8 \cdot 10^2$	$2.5 \cdot 10^{-2}$ ± $4.6 \cdot 10^{-3}$	$1.5 \cdot 10^3$ ± $2.7 \cdot 10^2$	2.2 ±$4.3 \cdot 10^{-2}$
	UCA	BG505 SOSIP.v4.1 (n=2)	Minimal binding; <10 RU						
		BG505 SOSIP.v4.1-GT1.2 (n=2)	$3.7 \cdot 10^3$ ± 50	$1.5 \cdot 10^{-2}$ ± $1.5 \cdot 10^{-3}$	$4.0 \cdot 10^3$ ± $3.6 \cdot 10^2$	$5.1 \cdot 10^2$ ± 60	$6.9 \cdot 10^{-4}$ ± $4.0 \cdot 10^{-5}$	$1.4 \cdot 10^3$ ± $2.4 \cdot 10^2$	1.4 ± 0.10
**PGV19**	*germline*	BG505 SOSIP.v4.1 (n=2)	Minimal binding; <10 RU						
		BG505 SOSIP.v4.1-GT1.2 (n=2)	$2.6 \cdot 10^4$ ± $2.5 \cdot 10^2$	$4.9 \cdot 10^{-4}$ ± $5.5 \cdot 10^{-6}$	19 ± 0.39	$2.1 \cdot 10^3$ ± 60	$2.4 \cdot 10^{-3}$ ± $3.0 \cdot 10^{-5}$	$1.1 \cdot 10^3$ ± 48	2.3 ± 0.10
**PGV20**	*germline*	BG505 SOSIP.v4.1 (n=2)	$9.9 \cdot 10^3$ ± $2.0 \cdot 10^3$	$2.3 \cdot 10^{-4}$ ± $4.0 \cdot 10^{-5}$	23 ± 0.57	$6.2 \cdot 10^5$ ± $4.3 \cdot 10^5$	0.32 ± 0.27	$4.1 \cdot 10^2$ ± $1.5 \cdot 10^2$	0.28 ± $3.7 \cdot 10^{-3}$
		BG505 SOSIP.v4.1-GT1.2 (n=3)	$3.4 \cdot 10^4$ ± $6.6 \cdot 10^3$	$< 10^{-5}$	< 1.0	$3.5 \cdot 10^6$ ± $3.3 \cdot 10^6$	5.7 ± 5.2	$2.2 \cdot 10^3$ ± $2.8 \cdot 10^2$	3.0 ± $3.3 \cdot 10^{-2}$

Table S1. SPR analysis of mature and germline/UCA NAb binding to BG505 SOSIP.v4.1 and GT1.2. Tabulated values are means ± S.E.M of n replicates. The parameters are fitted to the specific sensorgram binding data with a bivalent model; the constants for the initial, monovalent interaction are subscripted 1; for the interaction by the second Fab-arm of the IgG they are subscripted 2.

Data collection	BG505 SOSIP.v4.1-GT1.2 + Fab PGT124 + Fab gl-PGV20
Beamline	APS 23-IDD
Wavelength (Å)	1.0332
Detector	Pilatus
Space group	$P12_11$
Unit cell parameters (Å)	
a, b, c (Å), °	146.0, 157.6, 158.5, β=102.9
Resolution (Å)	49.7-3.8 (3.88-3.82)[a]
Total reflections	63,948
Unique reflections	5699
Redundancy	2.9 (3.0)[a]
Completeness (%)	95.7 (96.9)[a]
$<I/\sigma_i>$	4.3 (0.8)[a]
R_{sym}[b]	0.28 (>1.00)[a]
R_{pim}[c]	0.15 (0.73)[a]
$CC_{1/2}$[d]	0.78 (0.31)[a]
Wilson *B*-value (Å^2)	104.9
**Refinement statistics**	
Resolution (Å)	49.7-3.8 (3.9-3.8)[a]
Reflections (work)	63,928
Reflections (test)	3,180
R_{cryst}(%)[e]	27.0
R_{free}(%)[f]	30.1
Average B value (Å^2) (Proteins/Glycans)	122/115
**RMSD from ideal geometry**	
Bond length (Å)	0.003
Bond angles (°)	0.54
**Ramachandran statistics (%)**	
Favored	93.24
Allowed	6.08
Outliers	0.68
**PDB ID**	8E1P

[a]Numbers in parentheses are for highest resolution shell
[b]$R_{sym} = \Sigma_{hkl} \Sigma_i | I_{hkl,i} - <I_{hkl}> | / \Sigma_{hkl} \Sigma_i I_{hkl,i,}$ where $I_{hkl,i}$ is the scaled intensity of the ith measurement of reflection h, k, l, and $< I_{hkl}>$ is the average intensity for that reflection
[c]$R_{pim} = \Sigma_{hkl} (1/(n-1))1/2 \Sigma_i | I_{hkl,i} - <I_{hkl}> | / \Sigma_{hkl} \Sigma_i I_{hkl,i,}$ where n is the redundancy
[d]$CC_{1/2}$ = Pearson Correlation Coefficient between two random half datasets
[e]$R_{cryst} = \Sigma_{hkl} | Fo - Fc | / \Sigma_{hkl} | Fo | \times 100$
[f]R_{free} was calculated as for R_{cryst}, but on a test set comprising 5% of the data excluded from refinement

Table S2. X-ray data collection and refinement statistics. The data has been deposited in the Protein Data Bank under accession number 8E1P.
